# Supplementary material for: Synthesis of Novel Phosphorus-Containing Derivatives of 1,3,4-Trimethylglycoluril via the Birum–Oleksyszyn Reaction
Source: Int J Mol Sci. 2023 Dec 3;24(23):17082. doi: 10.3390/ijms242317082 (PMC10707106; doi:10.3390/ijms242317082)

checkCIF/PLATON report

Structure factors have been supplied for datablock(s) 284A_a

THIS REPORT IS FOR GUIDANCE ONLY. IF USED AS PART OF A REVIEW PROCEDURE FOR PUBLICATION, IT SHOULD NOT REPLACE THE EXPERTISE OF AN EXPERIENCED CRYSTALLOGRAPHIC REFEREE.

No syntax errors found. CIF dictionary Interpreting this report

Datablock: 284A_a

Bond precision:	C-C = 0.0022 A		Wavelength=0.71073
Cell:	a=23.3403(5)	b=10.3182(2)	c=24.6303(5)
	alpha=90	beta=109.626(1)	gamma=90
Temperature:	150 K		

	Calculated	Reported
Volume	5587.1(2)	5587.1(2)
Space group	C 2/c	C 2/c
Hall group	-C 2yc	-C 2yc
Moiety formula	2(C26 H27 N4 O5 P), 3(C2 H3 N)	?
Sum formula	C58 H63 N11 O10 P2	C29 H31.50 N5.50 O5 P
Mr	1136.13	568.07
Dx,g cm-3	1.351	1.351
Z	4	8
Mu (mm-1)	0.148	0.148
F000	2392.0	2392.0
F000’	2393.81	
h,k,lmax	33,14,35	32,14,35
Nref	8900	8204
Tmin,Tmax	0.974,0.994	0.692,0.746
Tmin’	0.957	

Correction method= # Reported T Limits: Tmin=0.692 Tmax=0.746
AbsCorr = ?

Data completeness= 0.922 Theta(max)= 31.000

R(reflections)= 0.0417(6752) wR2(reflections)= 0.1208(8204)

S = 1.039 Npar= 372

The following ALERTS were generated. Each ALERT has the format
test-name_ALERT_alert-type_alert-level.
Click on the hyperlinks for more details of the test.

Alert level C

```
PLAT052_ALERT_1_C Info on Absorption Correction Method   Not Given     Please Do !
PLAT244_ALERT_4_C Low    'Solvent' Ueq as Compared to Neighbors of        C29 Check
PLAT260_ALERT_2_C Large Average Ueq of Residue Including        N7      0.119 Check
PLAT366_ALERT_2_C Short? C(sp?)-C(sp?) Bond  C29      - C30      .       1.11 Ang.
PLAT911_ALERT_3_C Missing FCF Refl Between Thmin & STh/L=    0.600          5 Report
PLAT977_ALERT_2_C Check Negative Difference Density on H30C      .      -0.40 eA-3
```

Alert level G

```
PLAT045_ALERT_1_G Calculated and Reported Z Differ by a Factor ...      0.500 Check
PLAT300_ALERT_4_G Atom Site Occupancy of H30A       Constrained at        0.5 Check
PLAT300_ALERT_4_G Atom Site Occupancy of H30B       Constrained at        0.5 Check
PLAT300_ALERT_4_G Atom Site Occupancy of H30C       Constrained at        0.5 Check
PLAT413_ALERT_2_G Short Inter XH3 .. XHn     H21      ..H30C     .       2.03 Ang.
                                          3/2-x,-1/2+y,1/2-z  =      4_645 Check
PLAT883_ALERT_1_G No Info/Value for _atom_sites_solution_primary .     Please Do !
PLAT899_ALERT_4_G SHELXL2018 is Deprecated and Succeeded by SHELXL     2019/3 Note
PLAT910_ALERT_3_G Missing # of FCF Reflection(s) Below Theta(Min).          3 Note
PLAT912_ALERT_4_G Missing # of FCF Reflections Above STh/L=  0.600        667 Note
PLAT913_ALERT_3_G Missing # of Very Strong Reflections in FCF ....          3 Note
PLAT933_ALERT_2_G Number of HKL-OMIT Records in Embedded .res File          6 Note
PLAT978_ALERT_2_G Number C-C Bonds with Positive Residual Density.         13 Info
```

0 **ALERT level A** = Most likely a serious problem - resolve or explain
0 **ALERT level B** = A potentially serious problem, consider carefully
6 **ALERT level C** = Check. Ensure it is not caused by an omission or oversight
12 **ALERT level G** = General information/check it is not something unexpected

3 ALERT type 1 CIF construction/syntax error, inconsistent or missing data
6 ALERT type 2 Indicator that the structure model may be wrong or deficient
3 ALERT type 3 Indicator that the structure quality may be low
6 ALERT type 4 Improvement, methodology, query or suggestion
0 ALERT type 5 Informative message, check

It is advisable to attempt to resolve as many as possible of the alerts in all categories. Often the minor alerts point to easily fixed oversights, errors and omissions in your CIF or refinement strategy, so attention to these fine details can be worthwhile. In order to resolve some of the more serious problems it may be necessary to carry out additional measurements or structure refinements. However, the purpose of your study may justify the reported deviations and the more serious of these should normally be commented upon in the discussion or experimental section of a paper or in the "special_details" fields of the CIF. checkCIF was carefully designed to identify outliers and unusual parameters, but every test has its limitations and alerts that are not important in a particular case may appear. Conversely, the absence of alerts does not guarantee there are no aspects of the results needing attention. It is up to the individual to critically assess their own results and, if necessary, seek expert advice.

Publication of your CIF in IUCr journals

A basic structural check has been run on your CIF. These basic checks will be run on all CIFs submitted for publication in IUCr journals (*Acta Crystallographica*, *Journal of Applied Crystallography*, *Journal of Synchrotron Radiation*); however, if you intend to submit to *Acta Crystallographica Section C* or *E* or *IUCrData*, you should make sure that full publication checks are run on the final version of your CIF prior to submission.

Publication of your CIF in other journals

Please refer to the *Notes for Authors* of the relevant journal for any special instructions relating to CIF submission.

PLATON version of 10/05/2023; check.def file version of 10/05/2023

Datablock 284A_a - ellipsoid plot

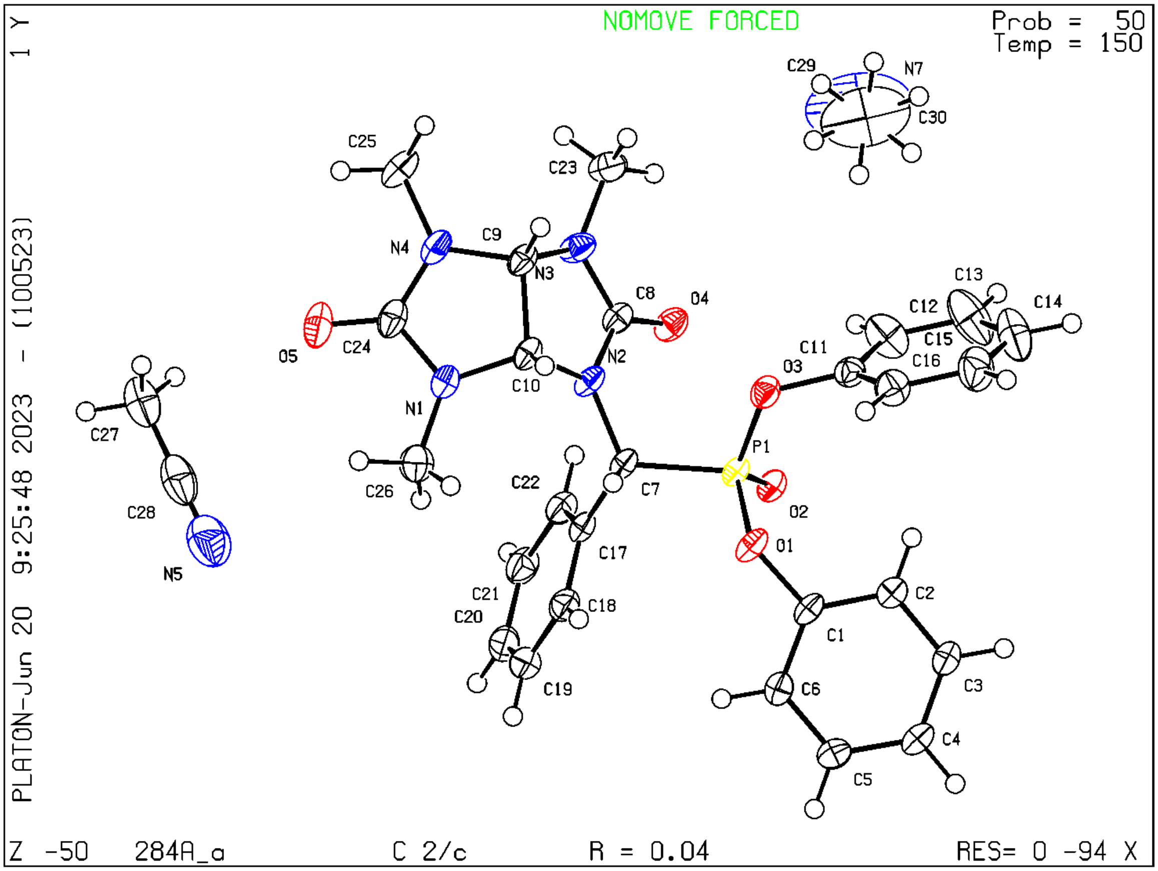

Supplement: Supplementary file 1 [file ijms-24-17082-s001.zip › 4a'_CheckCIF.pdf]
