# Supplementary material for: Synthesis of Novel Phosphorus-Containing Derivatives of 1,3,4-Trimethylglycoluril via the Birum–Oleksyszyn Reaction
Source: Int J Mol Sci. 2023 Dec 3;24(23):17082. doi: 10.3390/ijms242317082 (PMC10707106; doi:10.3390/ijms242317082)
Supplement: Supplementary file 1 [file ijms-24-17082-s001.zip › Supplemetary_mat_Synthesis_of_novel_phosphorous_containing_derivatives_re.docx]

**
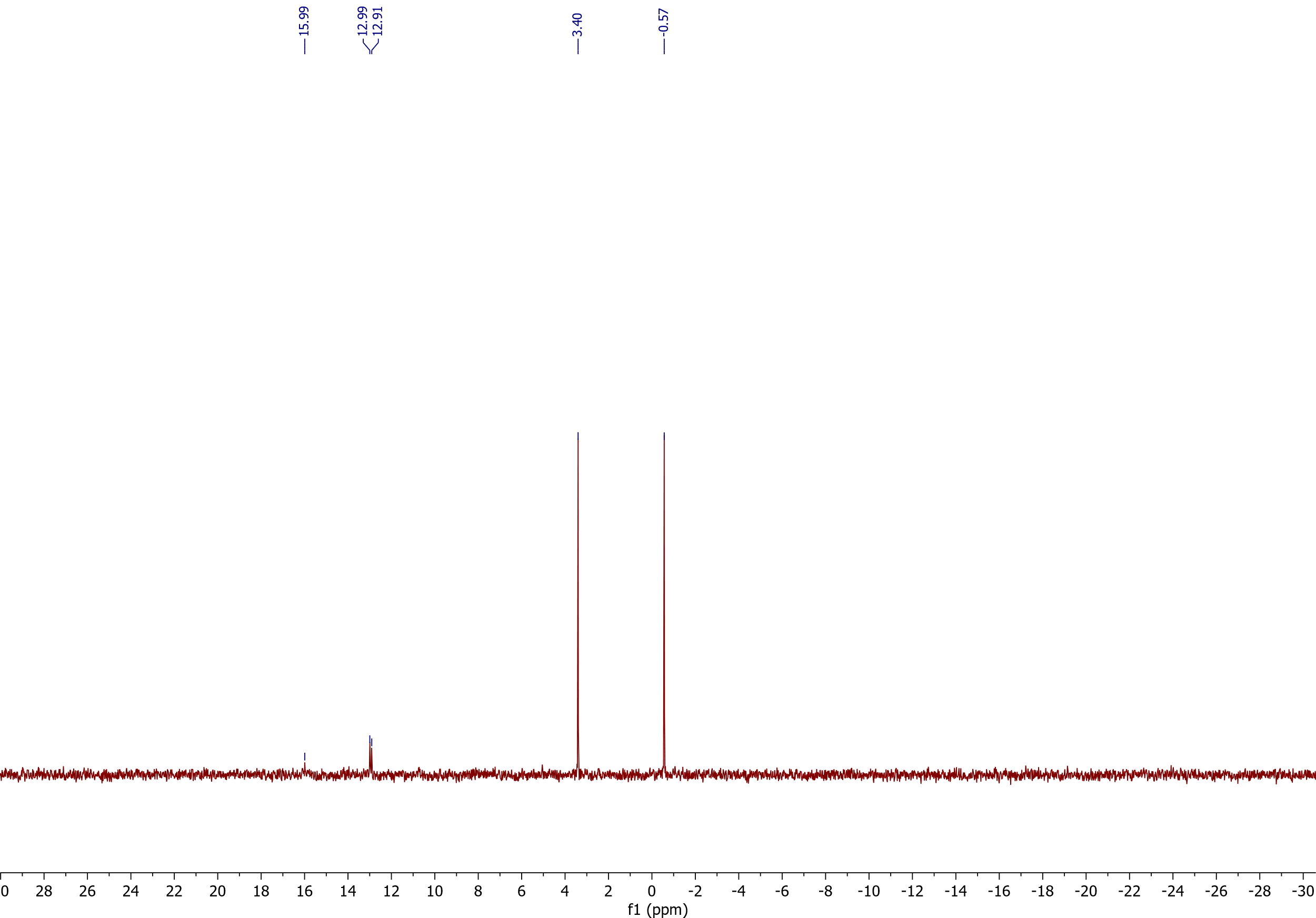
**

**Figure S1**. ^31^P NMR ((162 MHz, DMSO-d6) of the reaction mixture using acetic acid as a solvent after two hours at 80 °C


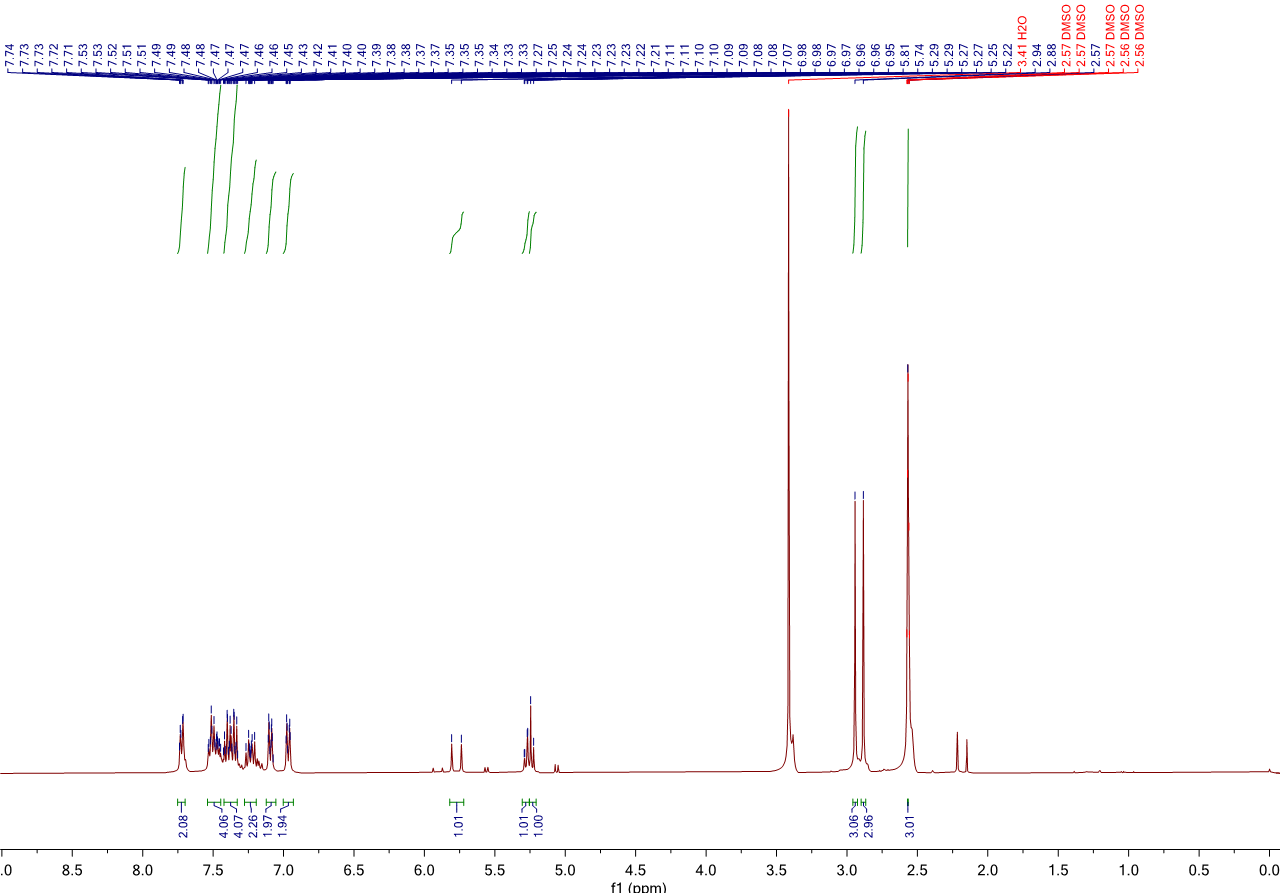


**Figure S2**. ^1^H NMR (400 MHz, DMSO-*d*_6_) of compound (**4a′**)


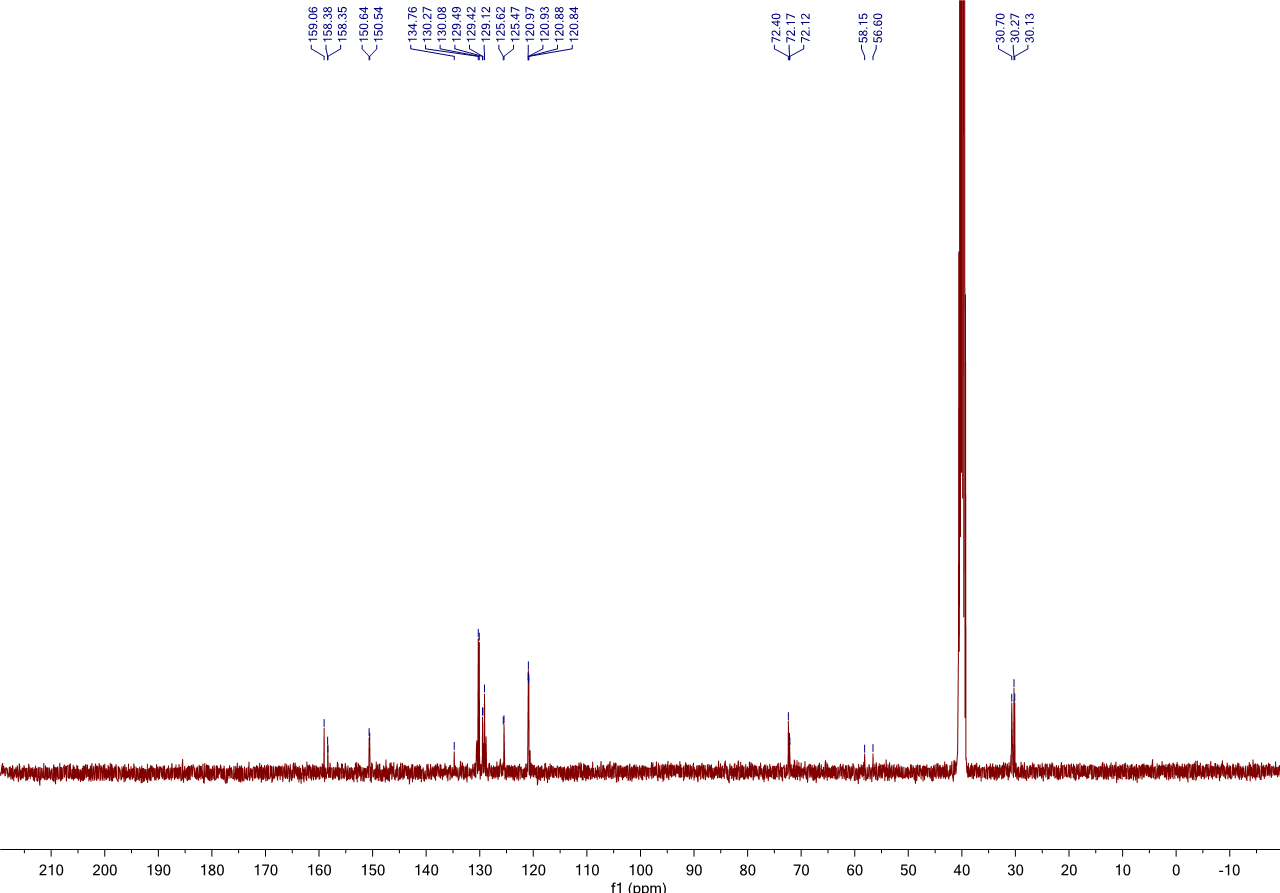


**Figure S3**. ^13^C NMR (101 MHz, DMSO-*d*_6_) of compound (**4a′**)


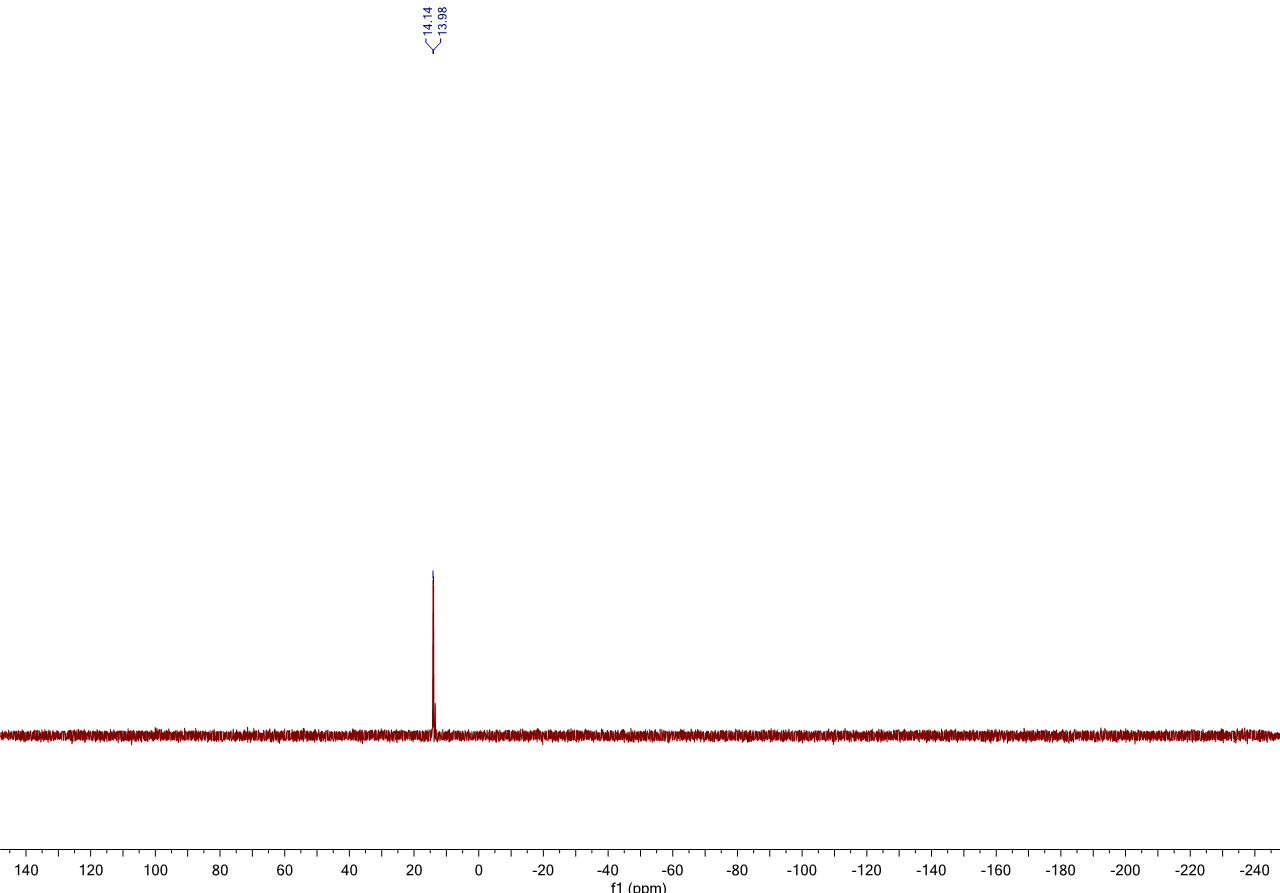


**Figure S4**. ^31^P NMR (162 MHz, DMSO-d6) of compound (**4a′**)


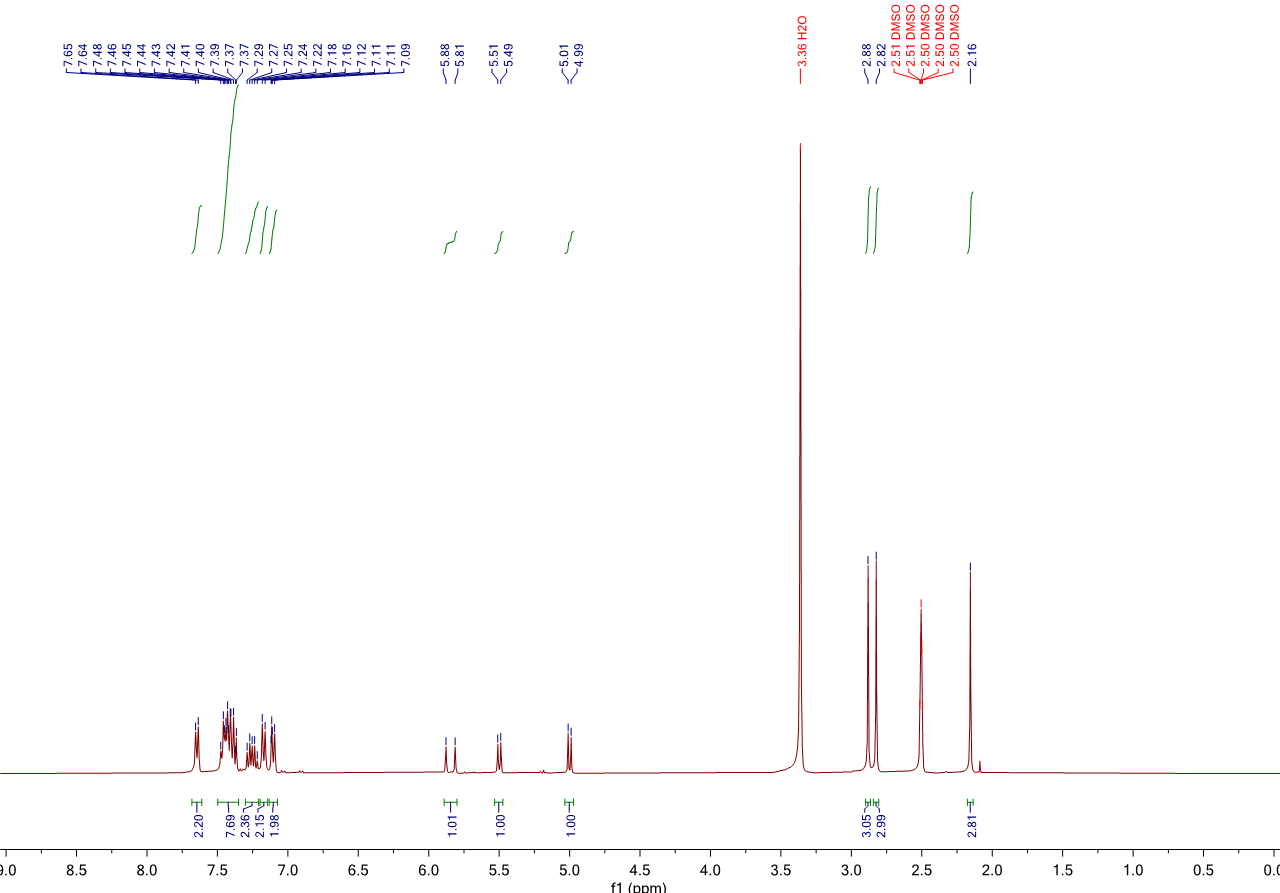


**Figure S5**. ^1^H NMR (400 MHz, DMSO-*d*_6_) of compound (**4a″**)


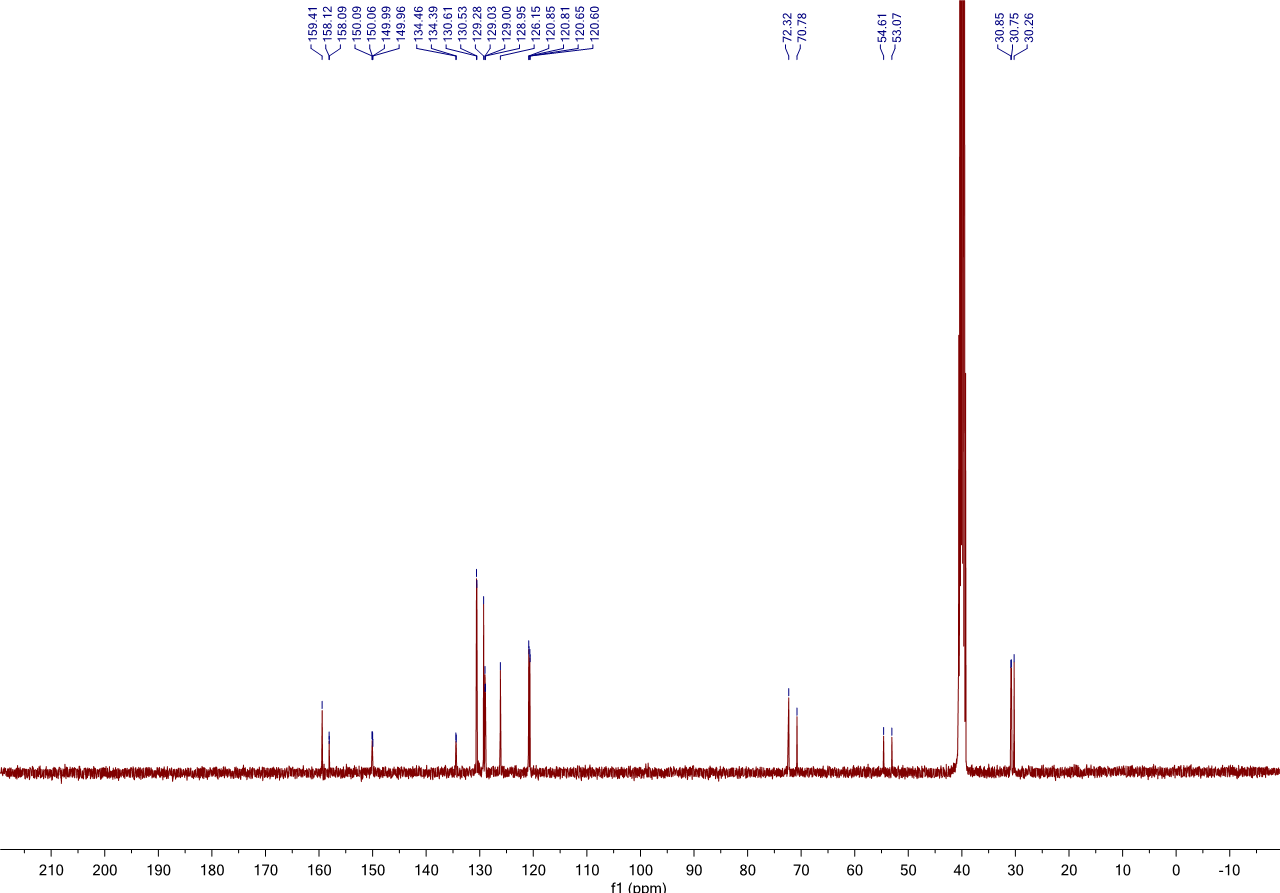


**Figure S6**. ^13^C NMR (101 MHz, DMSO-*d*_6_) of compound (**4a″**)


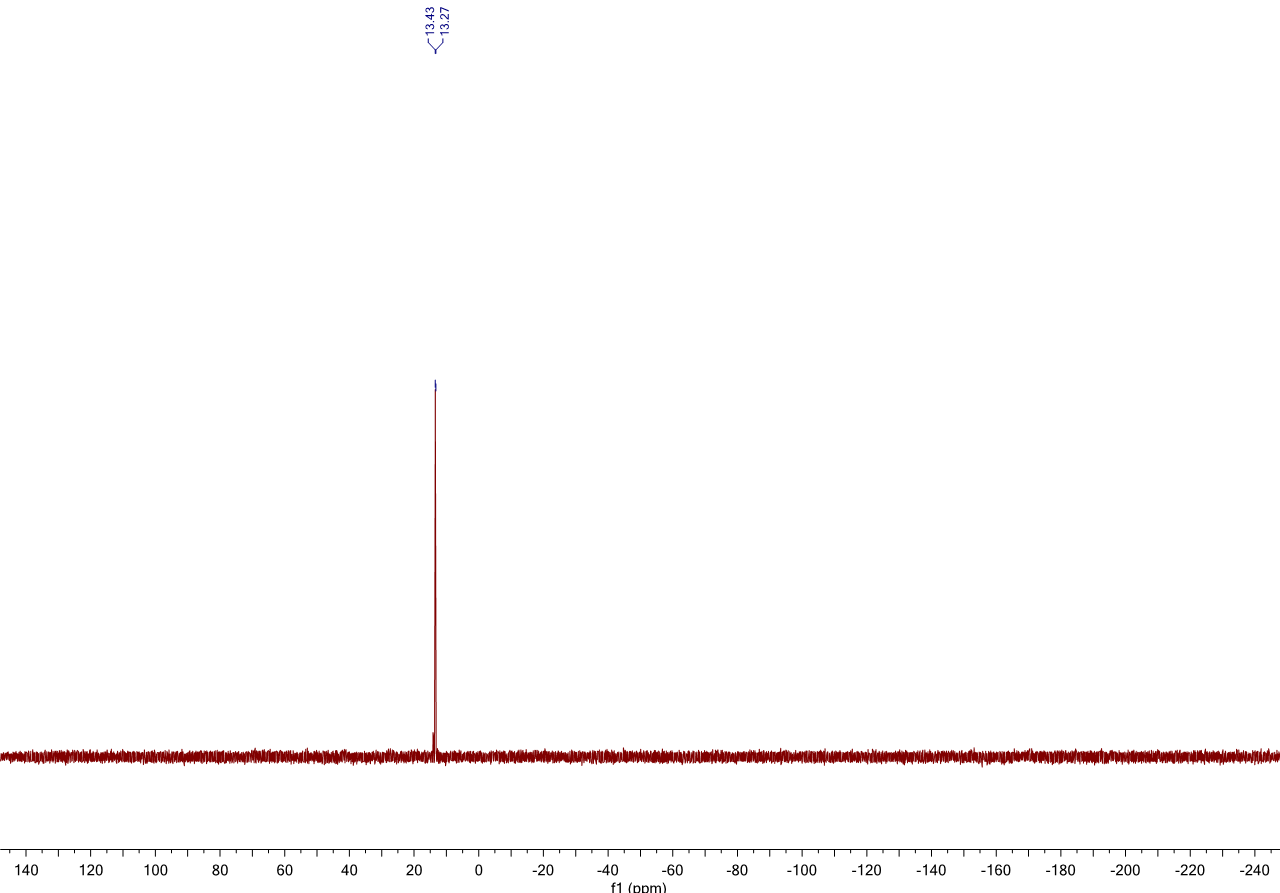


**Figure S7**. ^31^P NMR (162 MHz, DMSO-d6) of compound (**4a″**)


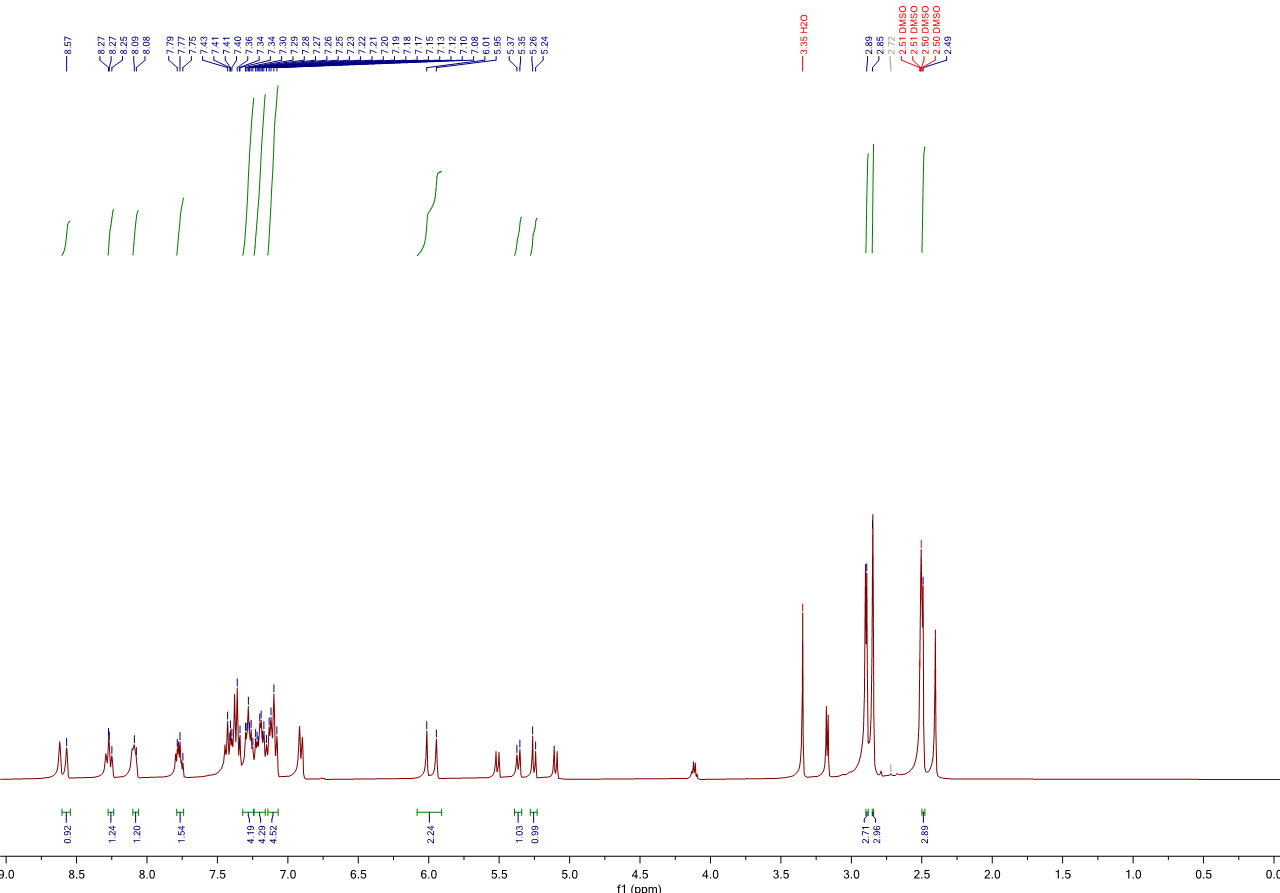


**Figure S8**. ^1^H NMR (400 MHz, DMSO-*d*_6_) of compound (**4b′**)


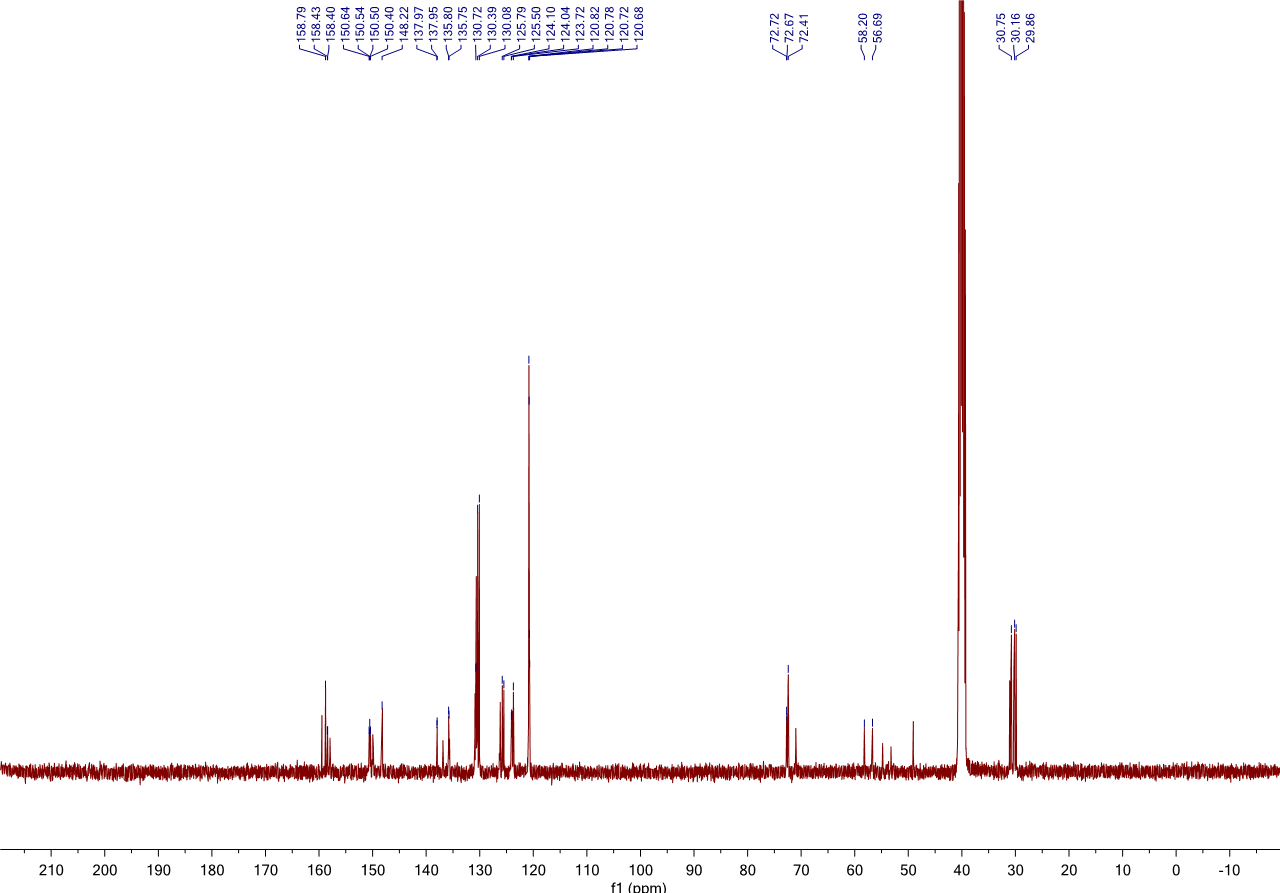


**Figure S9**. ^13^C NMR (101 MHz, DMSO-*d*_6_) of compound (**4b′**)


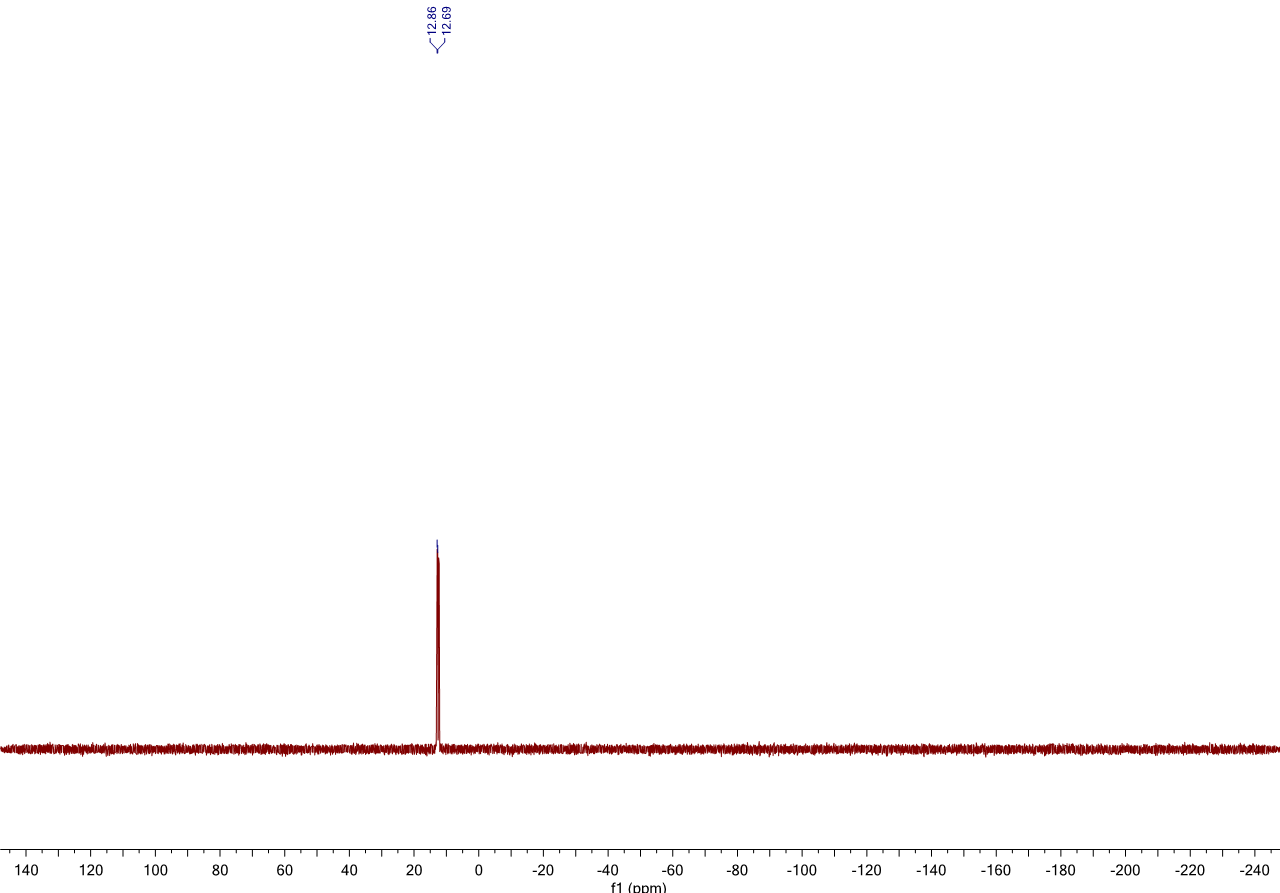


**Figure S10**. ^31^P NMR (162 MHz, DMSO-d6) of compound (**4b′**)


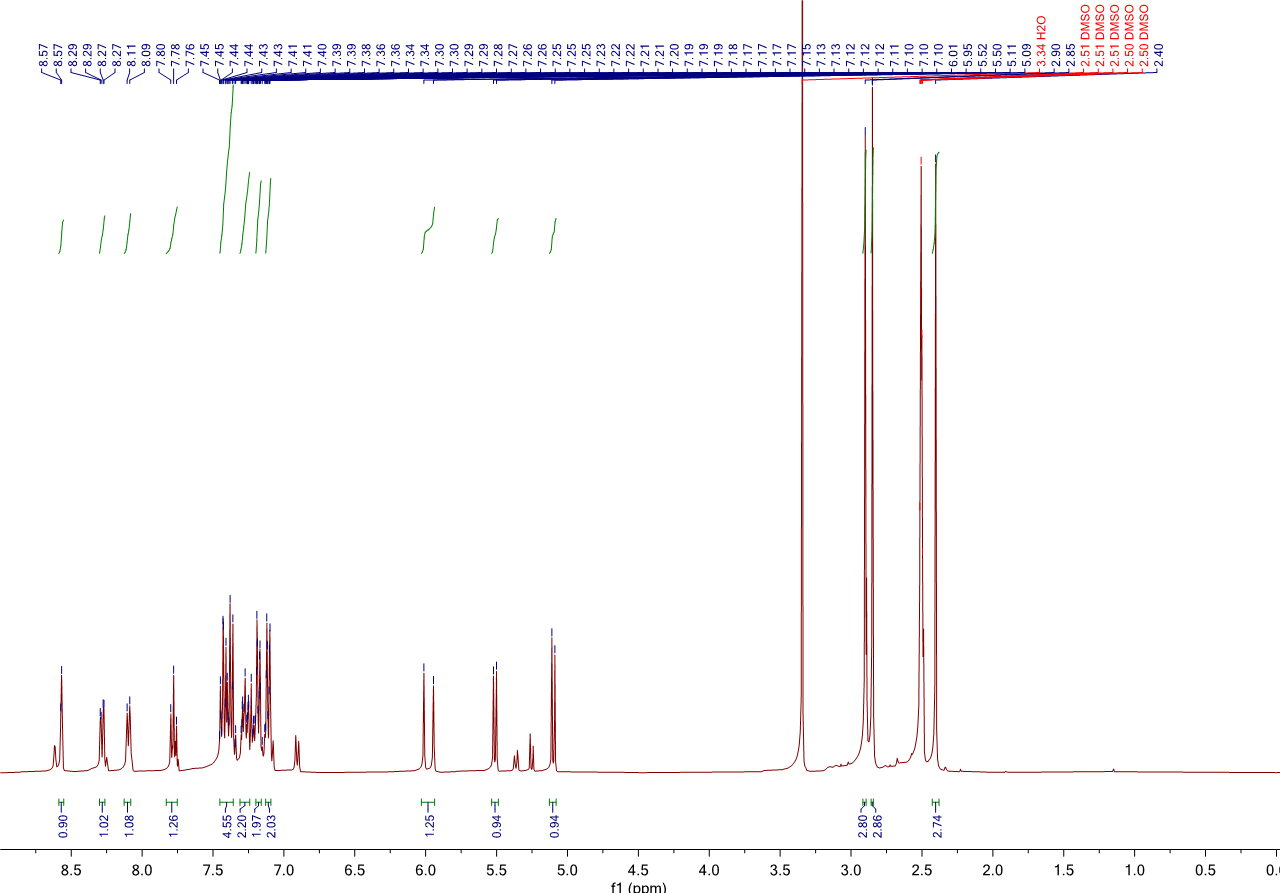


**Figure S11**. ^1^H NMR (400 MHz, DMSO-*d*_6_) of compound (**4b″**)


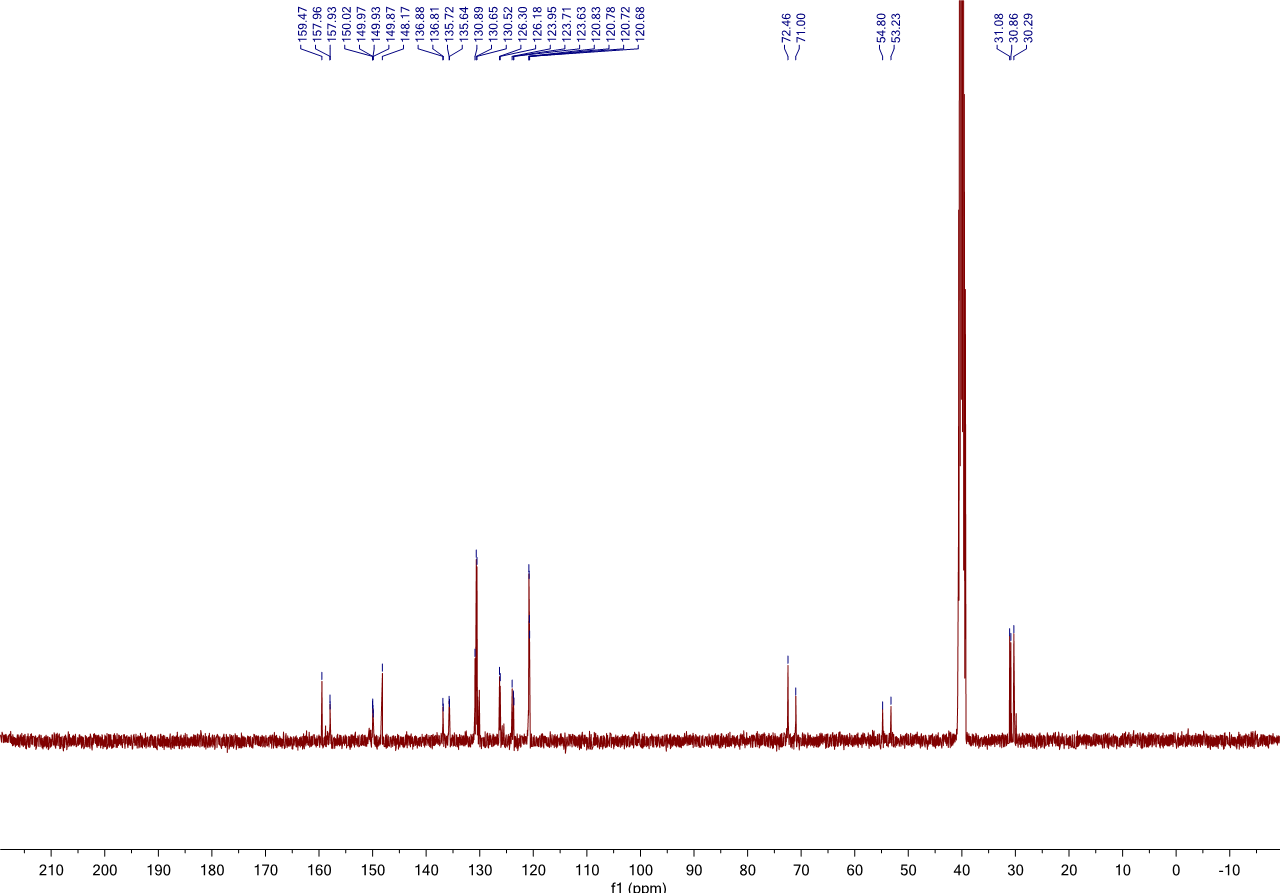


**Figure S12**. ^13^C NMR (101 MHz, DMSO-*d*_6_) of compound (**4b″**)


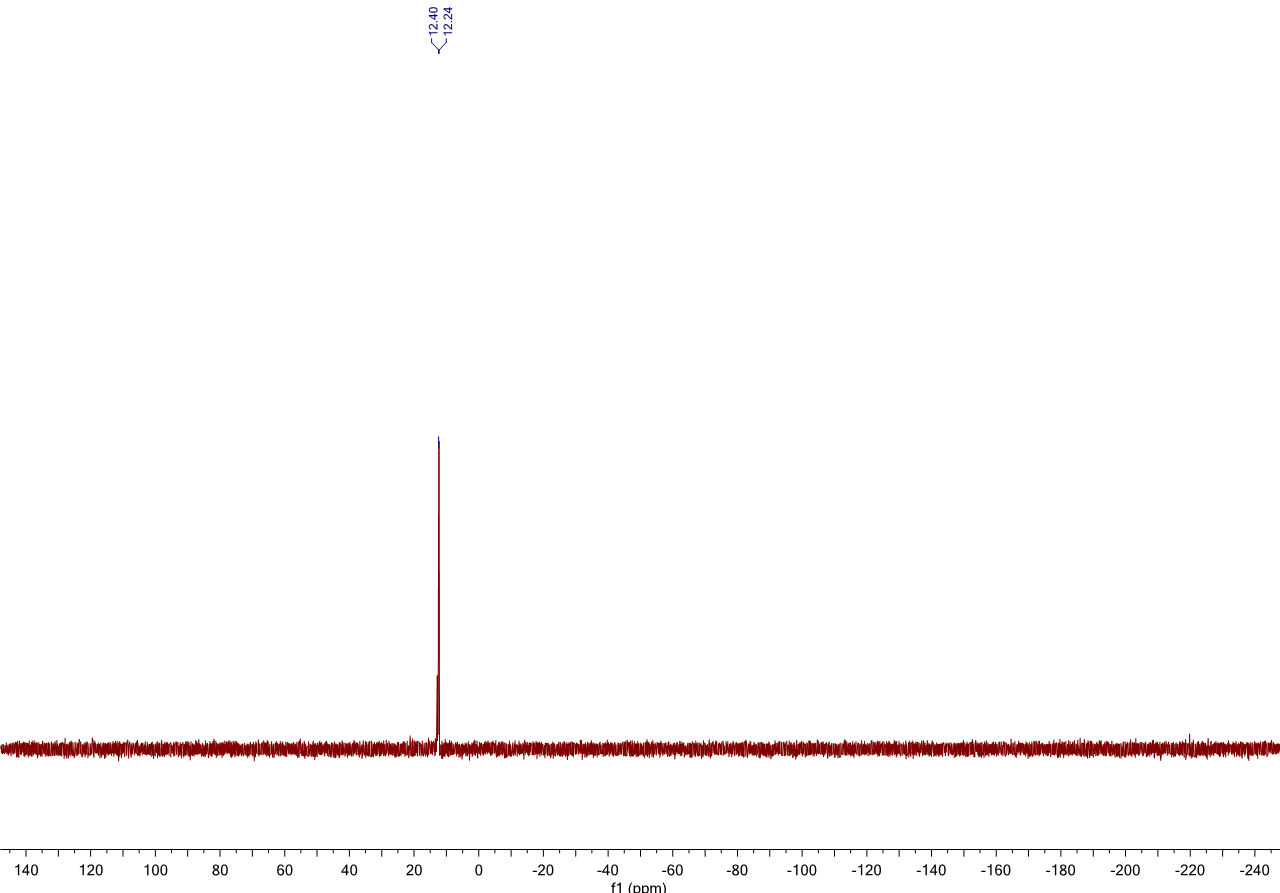


**Figure S13**. ^31^P NMR (162 MHz, DMSO-d6) of compound (**4b″**)


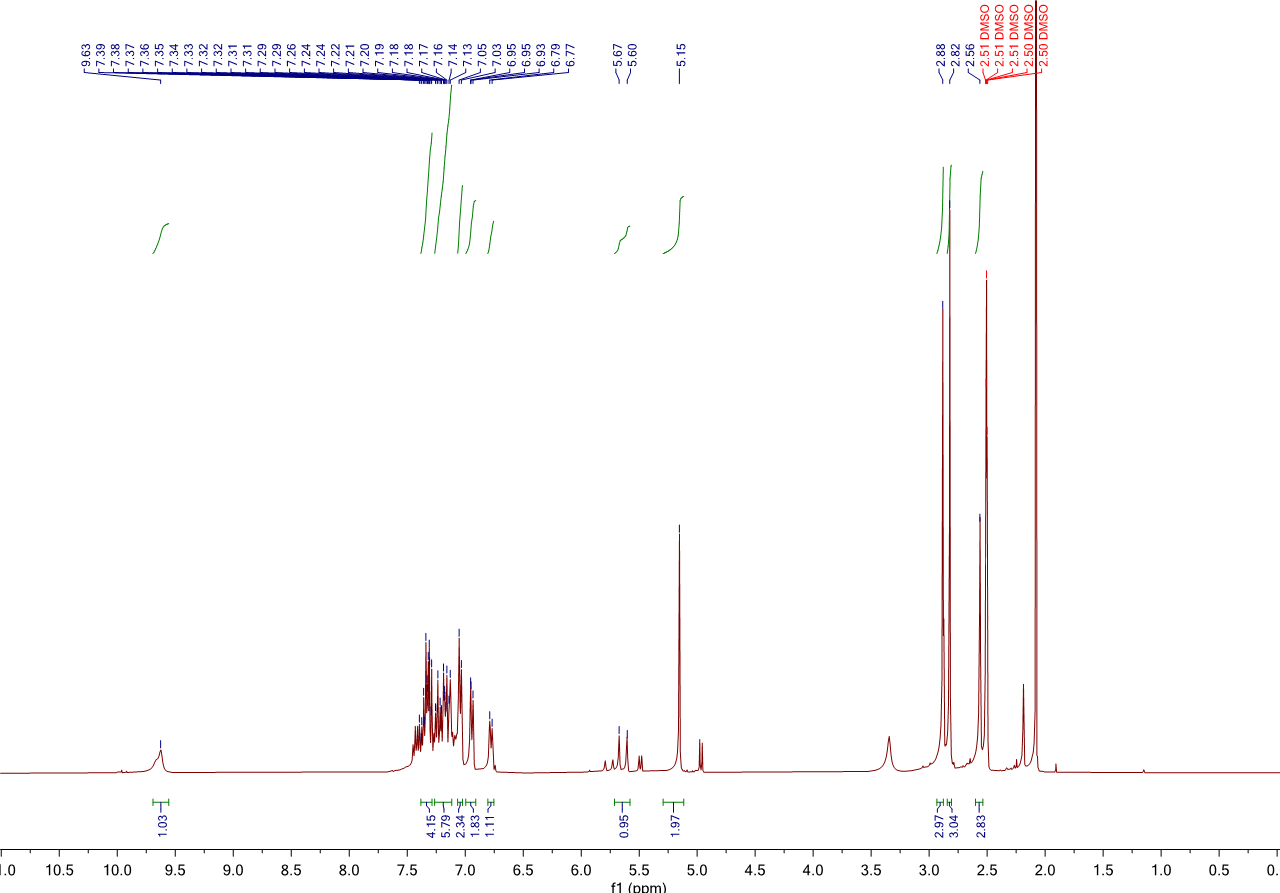


**Figure S14**. ^1^H NMR (400 MHz, DMSO-*d*_6_) of compound (**4с′**)


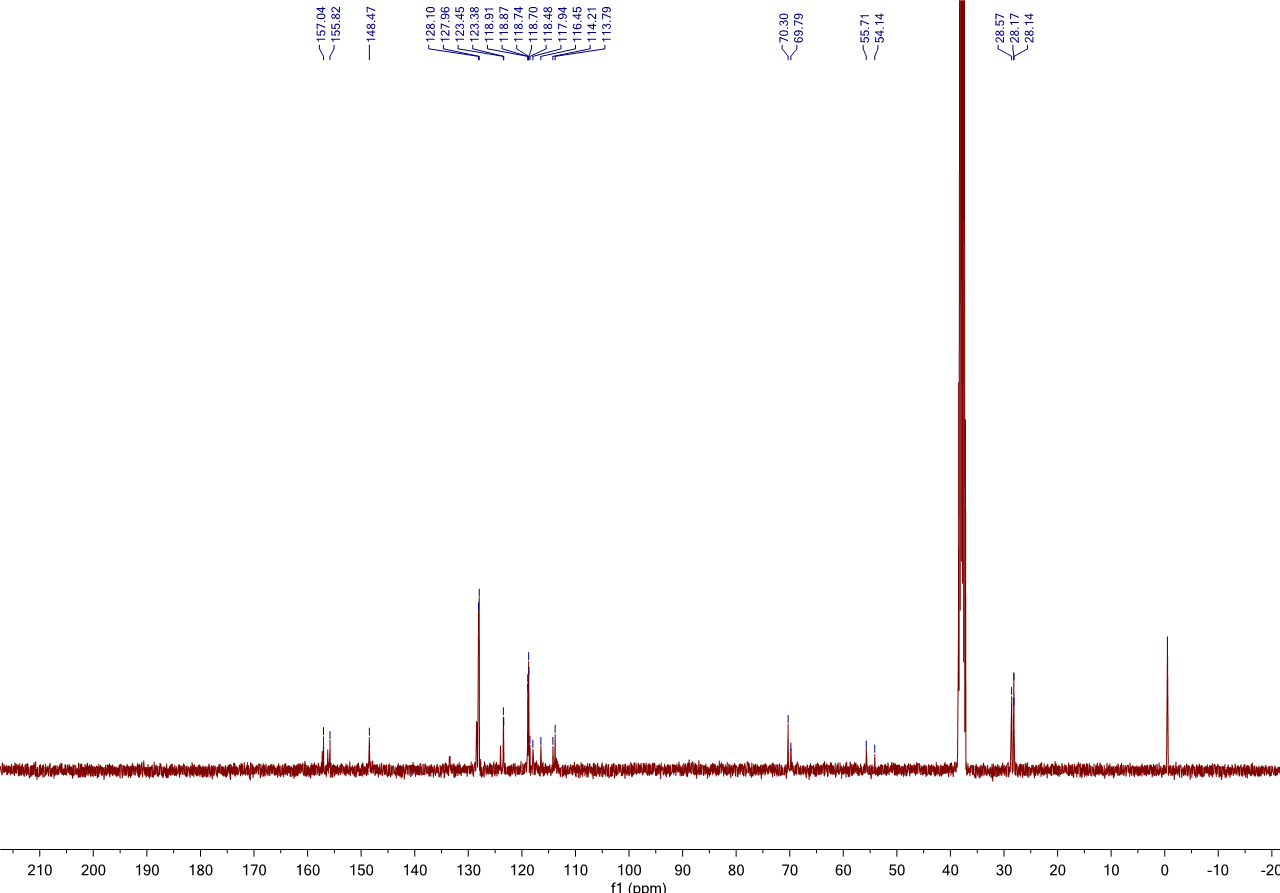


**Figure S15**. ^13^C NMR (101 MHz, DMSO-*d*_6_) of compound (**4с′**)


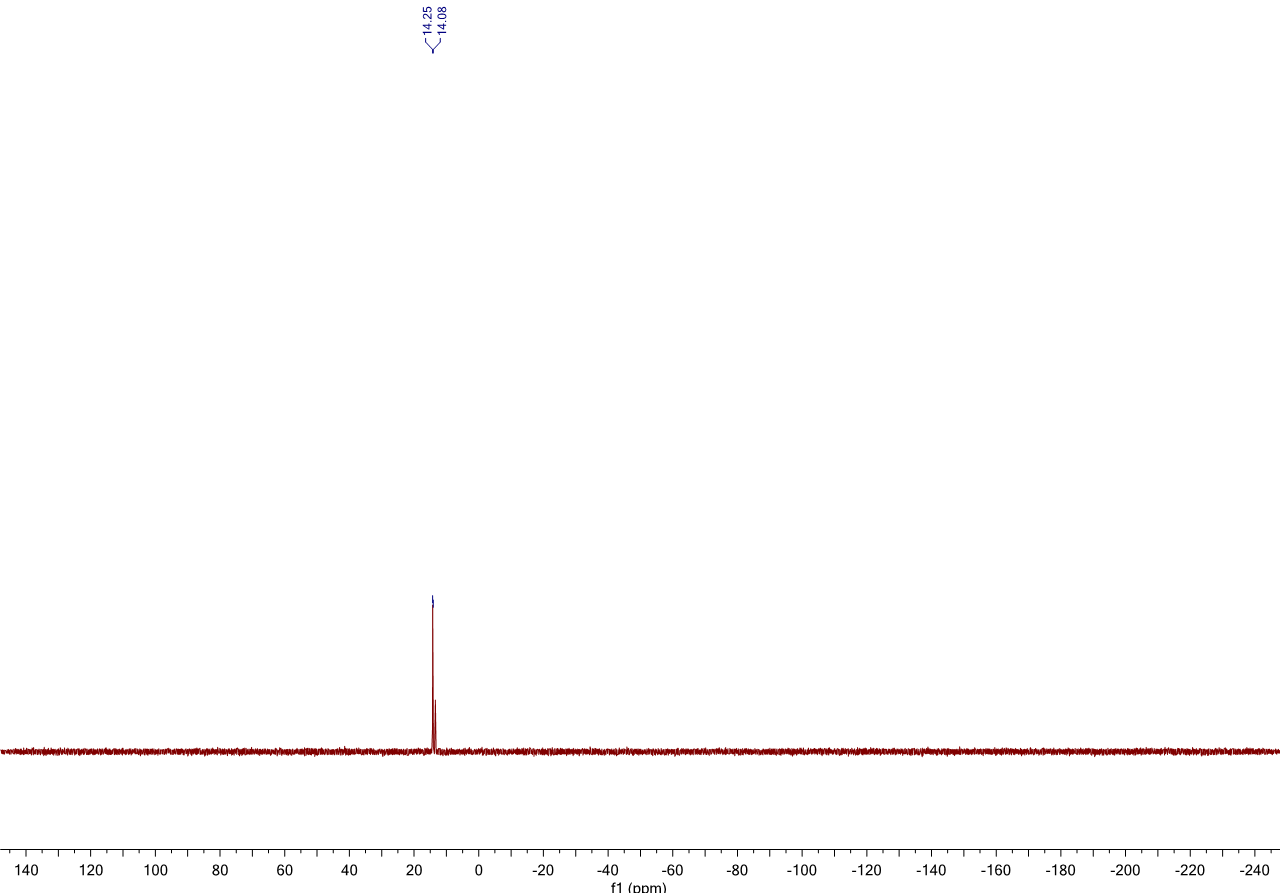


**Figure S16**. ^31^P NMR (162 MHz, DMSO-d6) of compound (**4с′**)


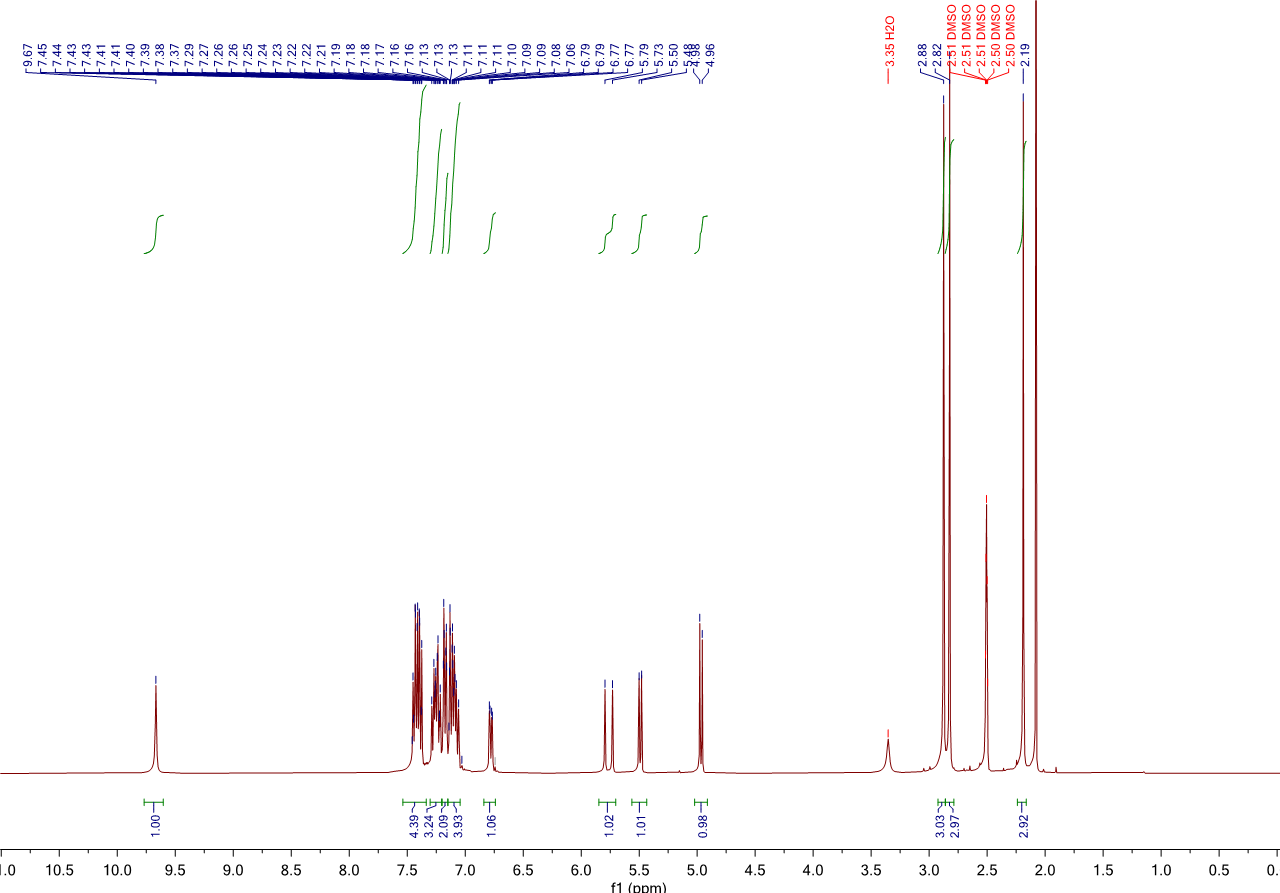


**Figure S17**. ^1^H NMR (400 MHz, DMSO-*d*_6_) of compound (**4с″**)


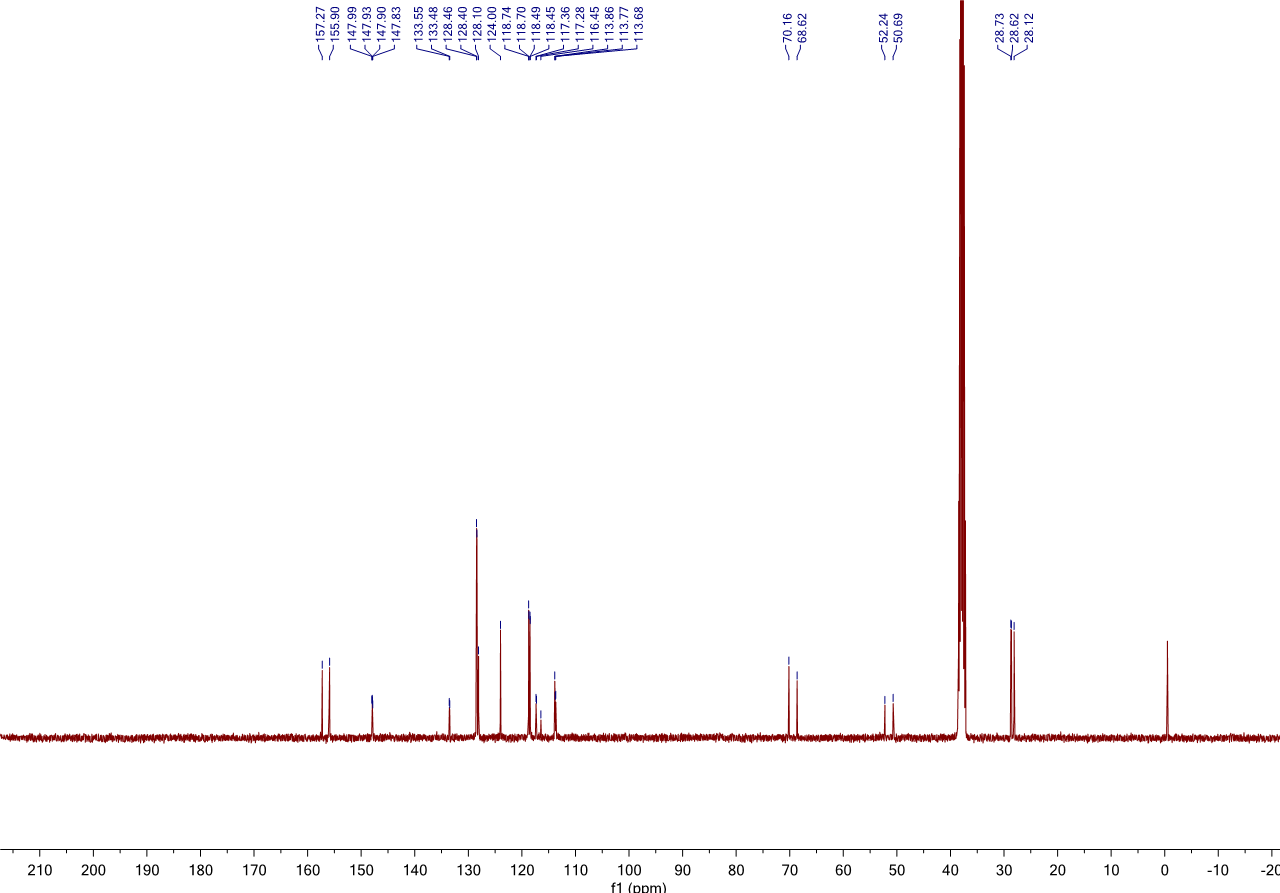


**Figure S18**. ^13^C NMR (101 MHz, DMSO-*d*_6_) of compound (**4с″**)


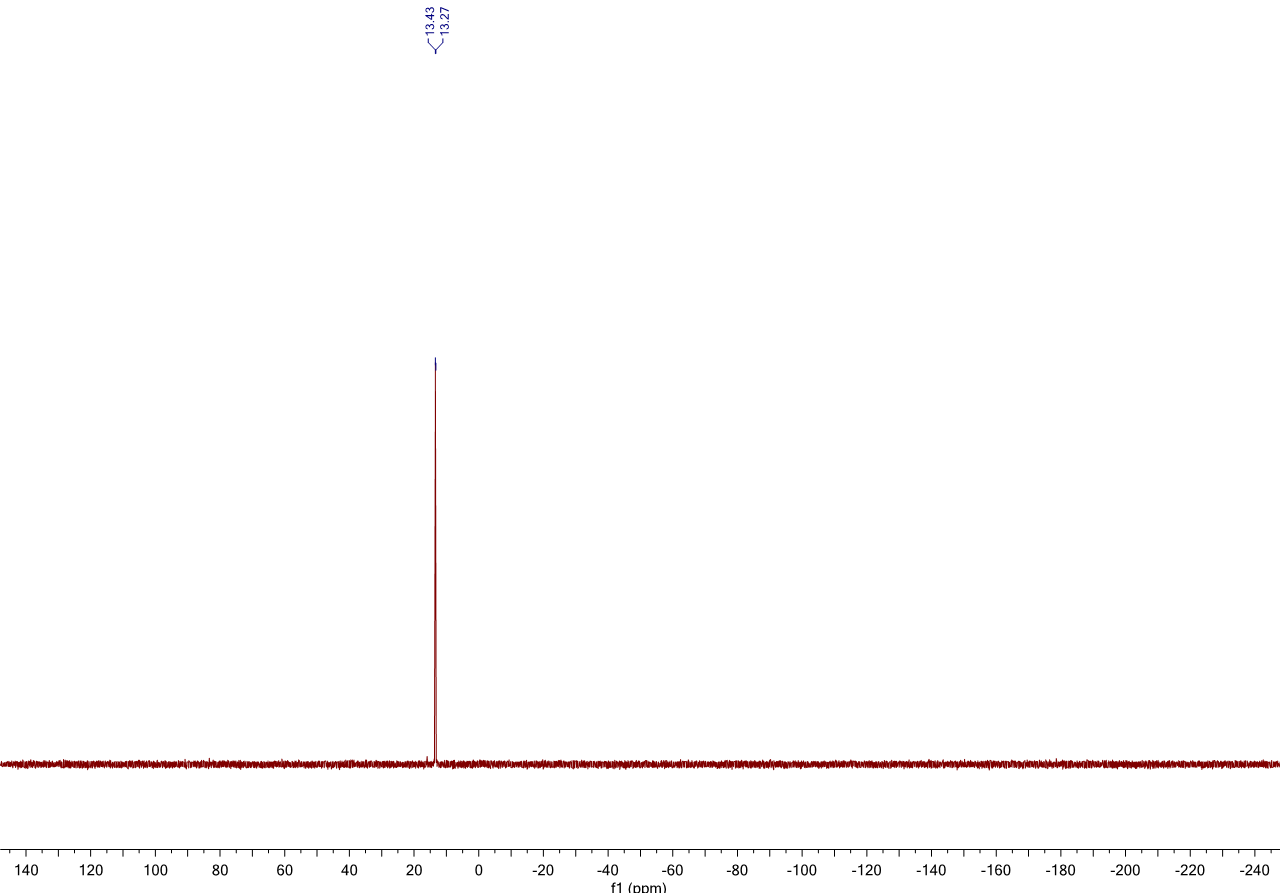


**Figure S19**. ^31^P NMR (162 MHz, DMSO-d6) of compound (**4с″**)


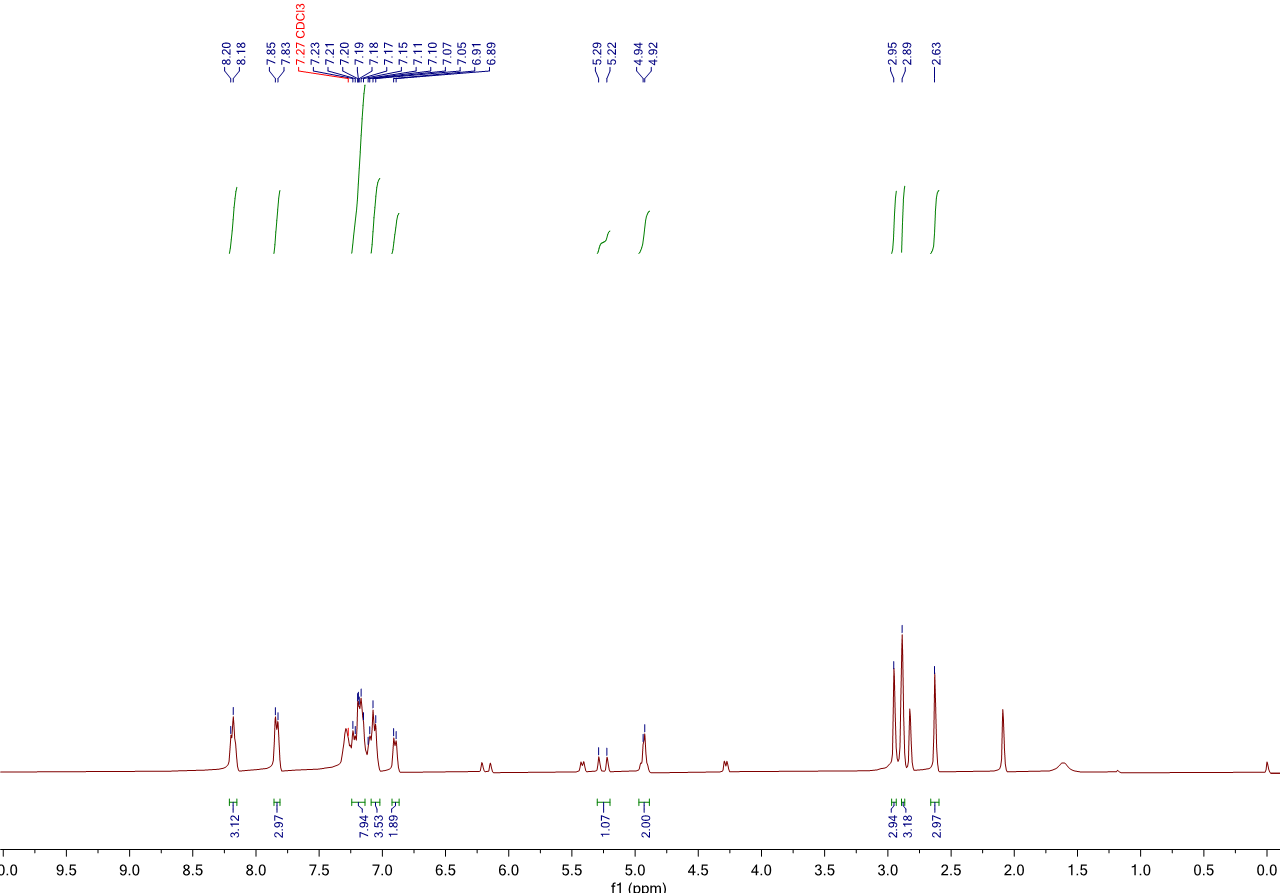


**Figure S20**. ^1^H NMR (400 MHz, Chloroform-*d*) of compound (**4d′**)


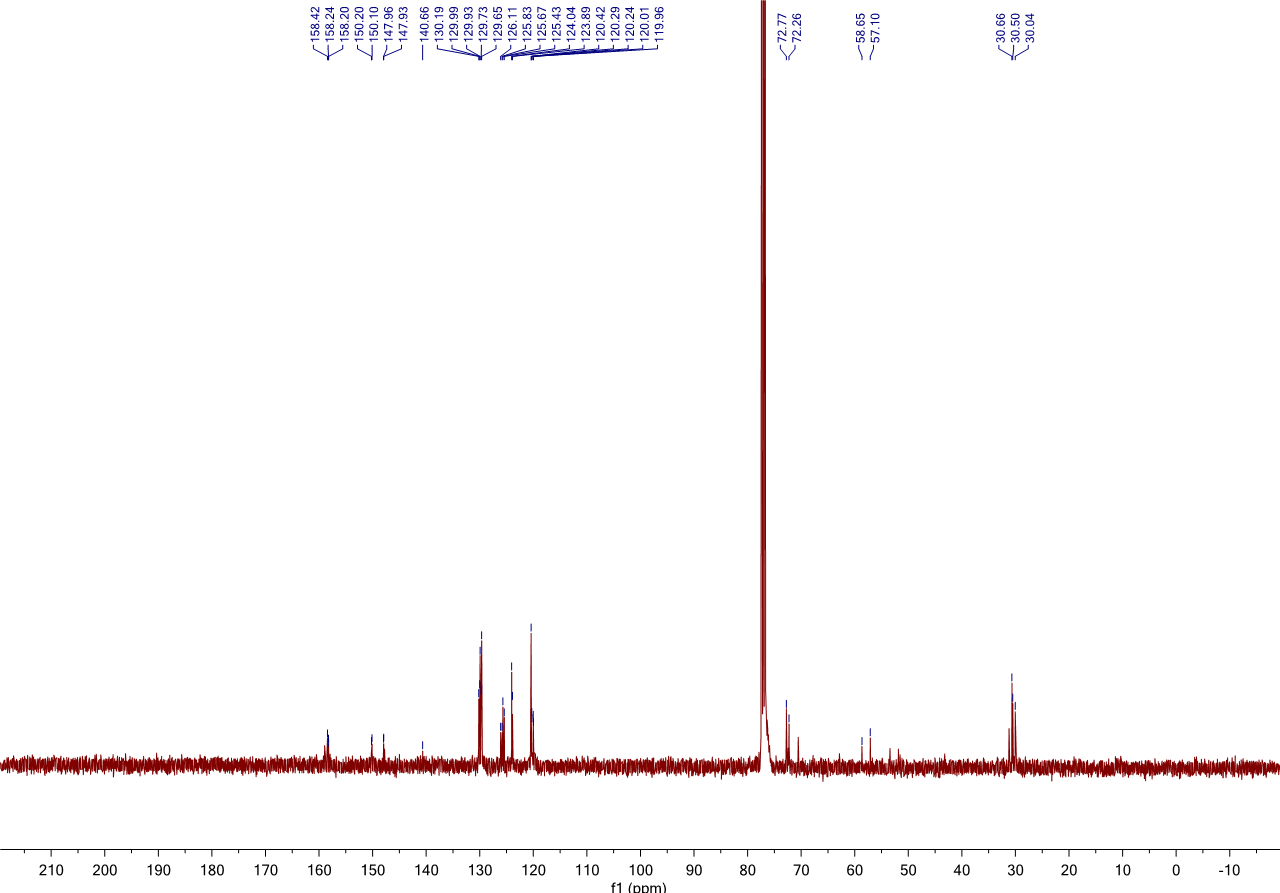


**Figure S21**. ^13^C NMR (101 MHz, Chloroform-*d*) of compound (**4d′**)


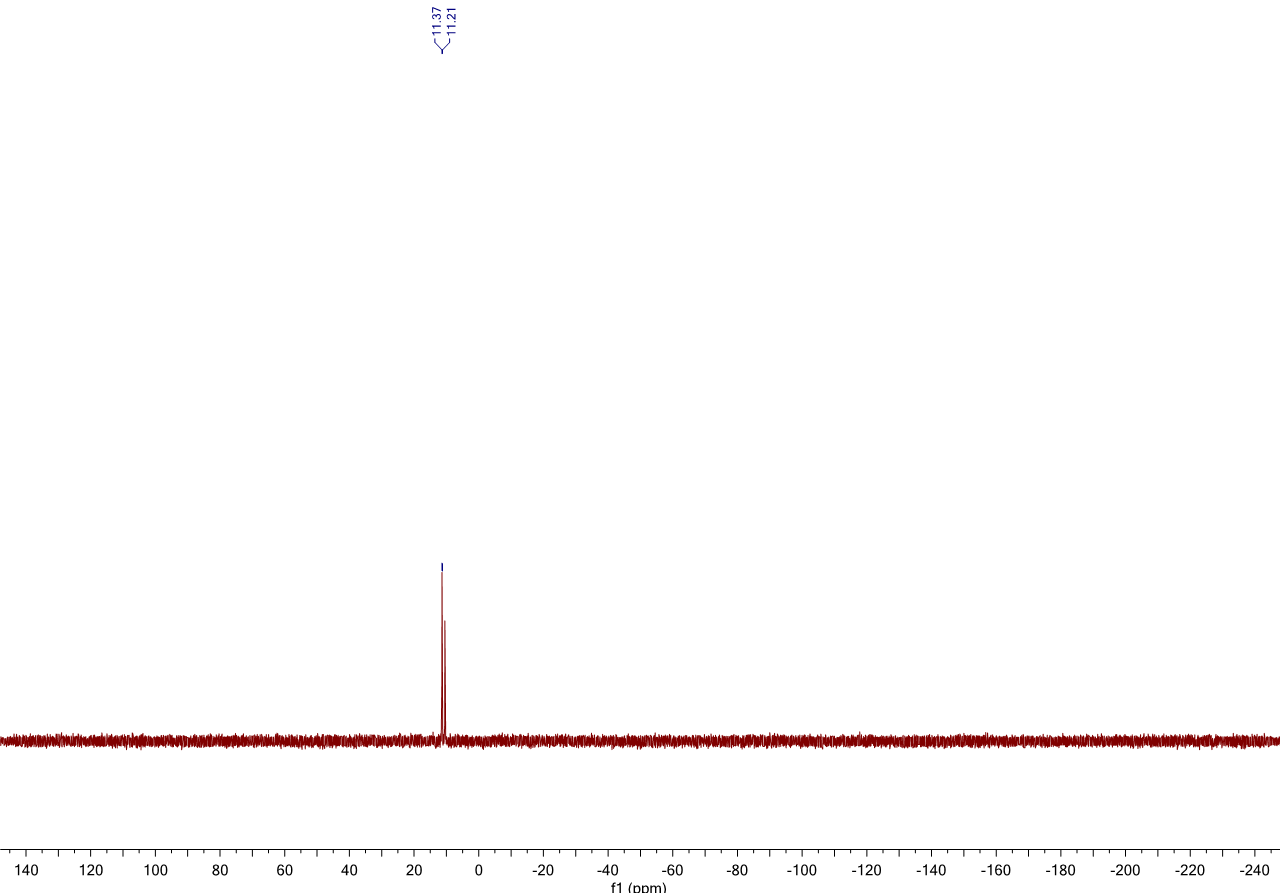


**Figure S22**. ^31^P NMR (162 MHz, Chloroform-*d*) of compound (**4d′**)


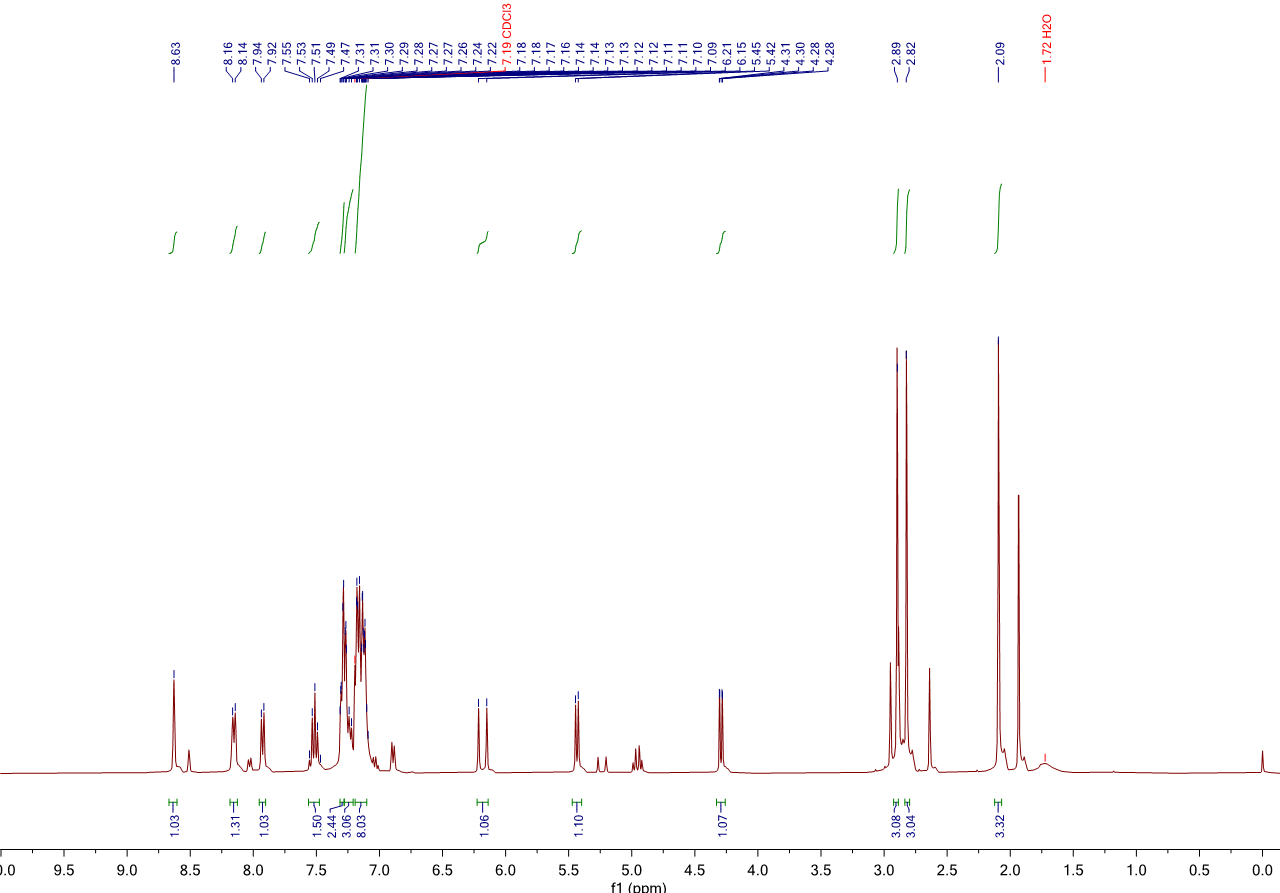


**Figure S23**. ^1^H NMR (400 MHz, Chloroform-*d*) of compound (**4d″**)


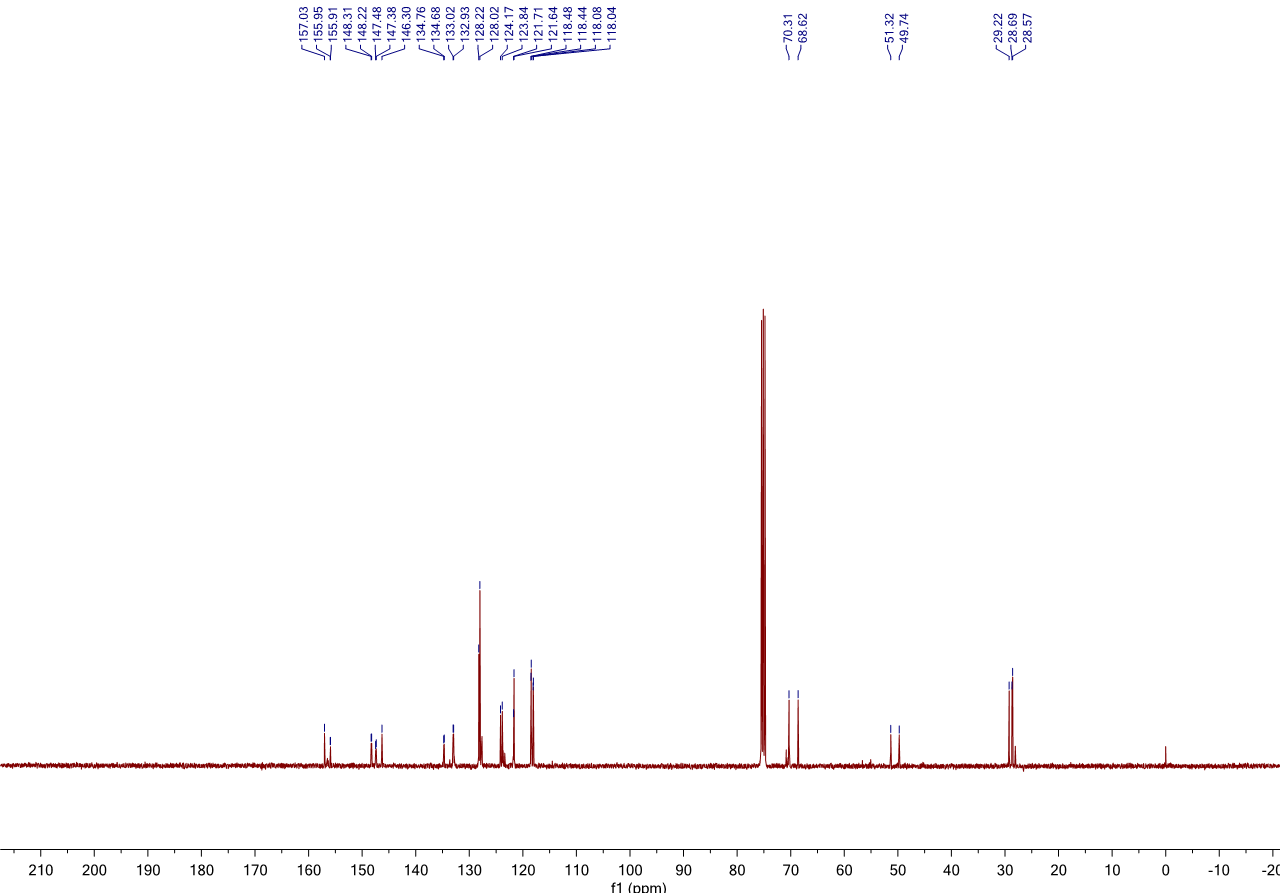


**Figure S24**. ^13^C NMR (101 MHz, Chloroform-*d*) of compound (**4d″**)


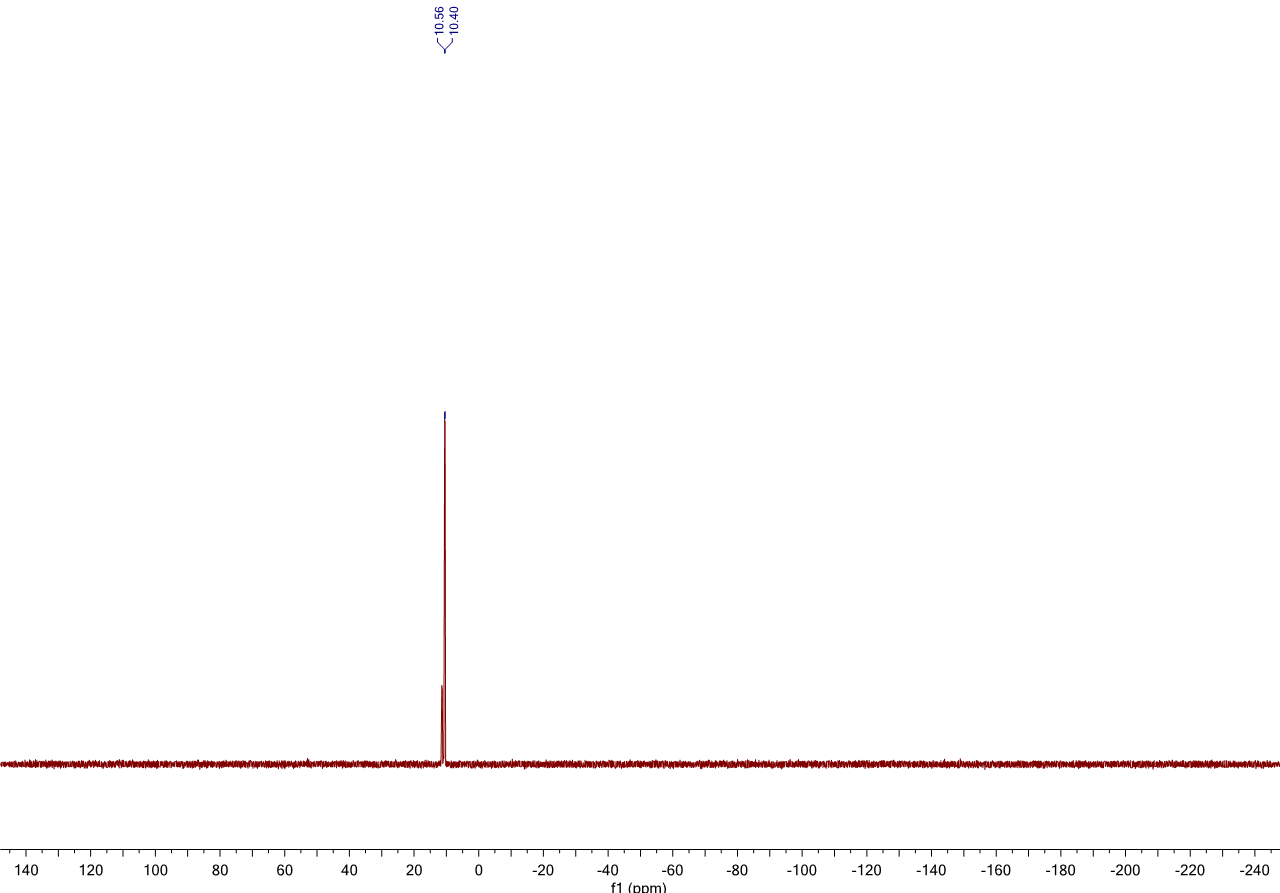


**Figure S25**. ^31^P NMR (162 MHz, Chloroform-*d*) of compound (**4d″**)


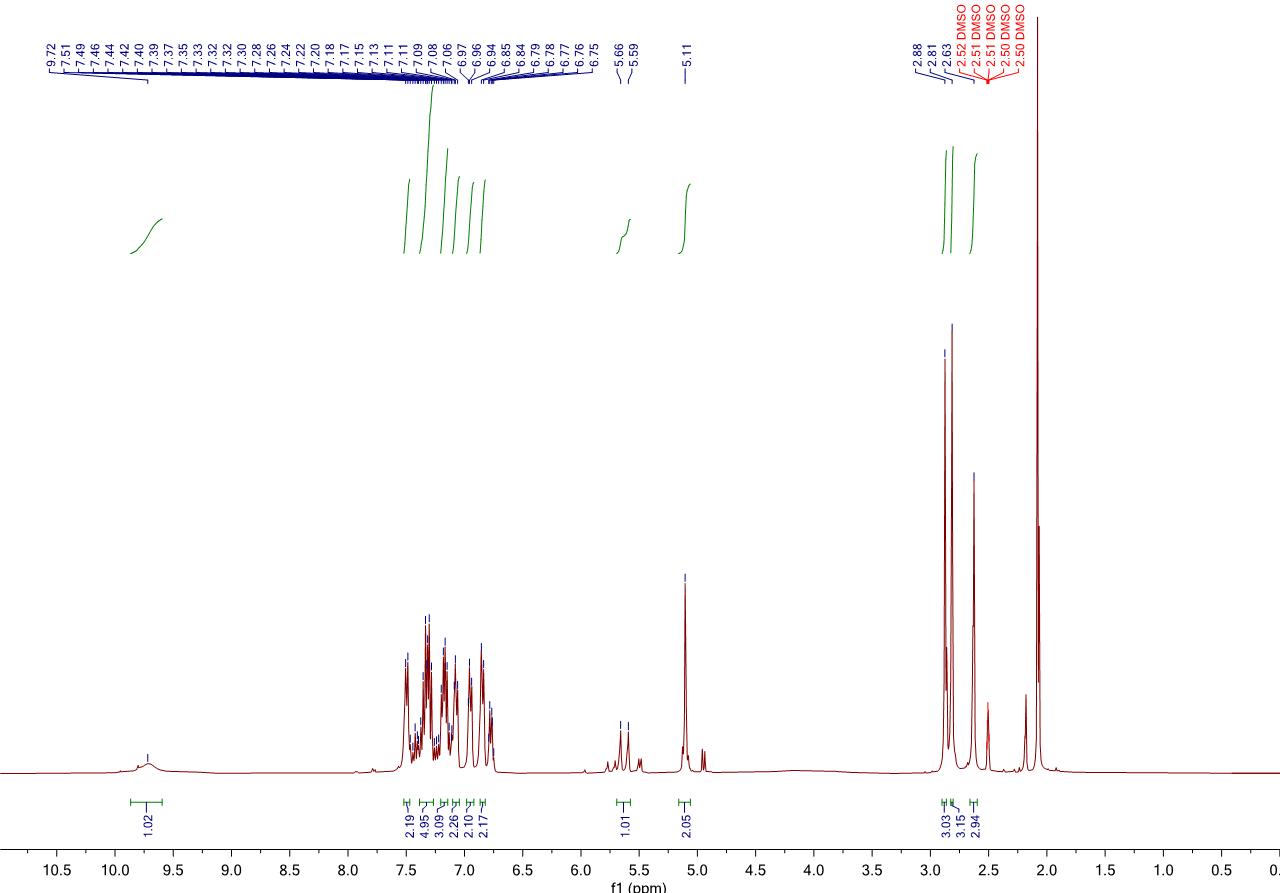


**Figure S26**. ^1^H NMR (400 MHz, DMSO-*d*_6_) of compound (**4e′**)


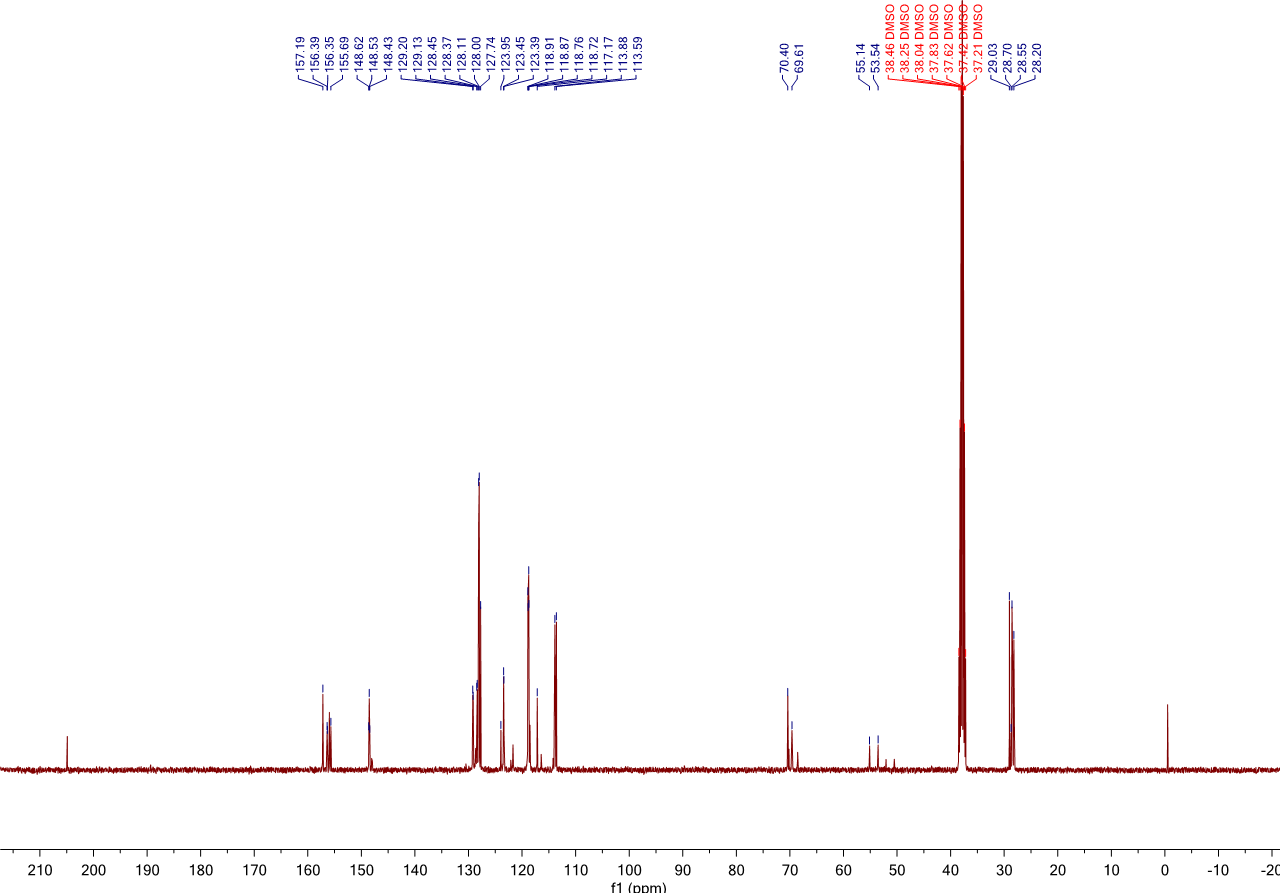


**Figure S27**. ^13^C NMR (101 MHz, DMSO-*d*_6_) of compound (**4e′**)


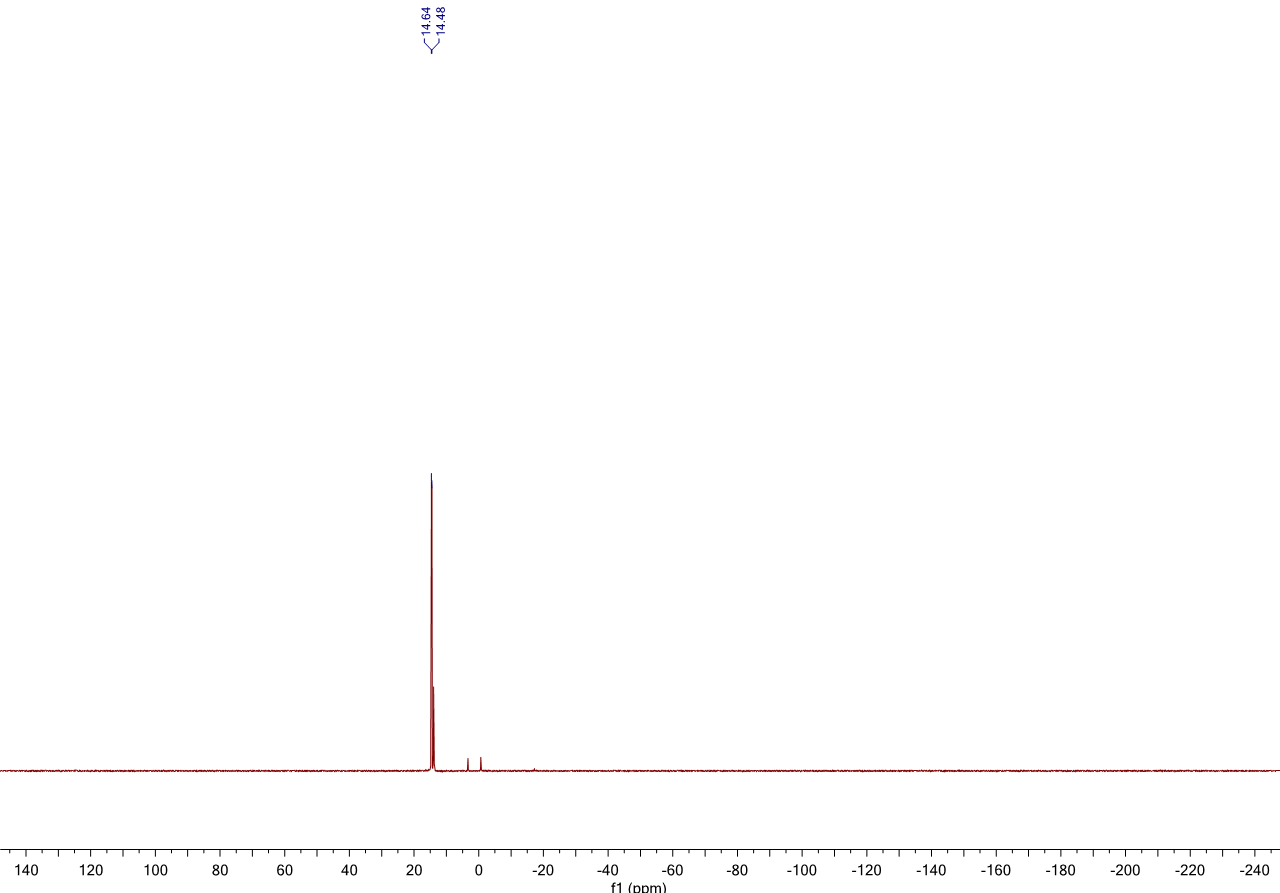


**Figure S28**. ^31^P NMR (162 MHz, DMSO-d6) of compound (**4e′**)


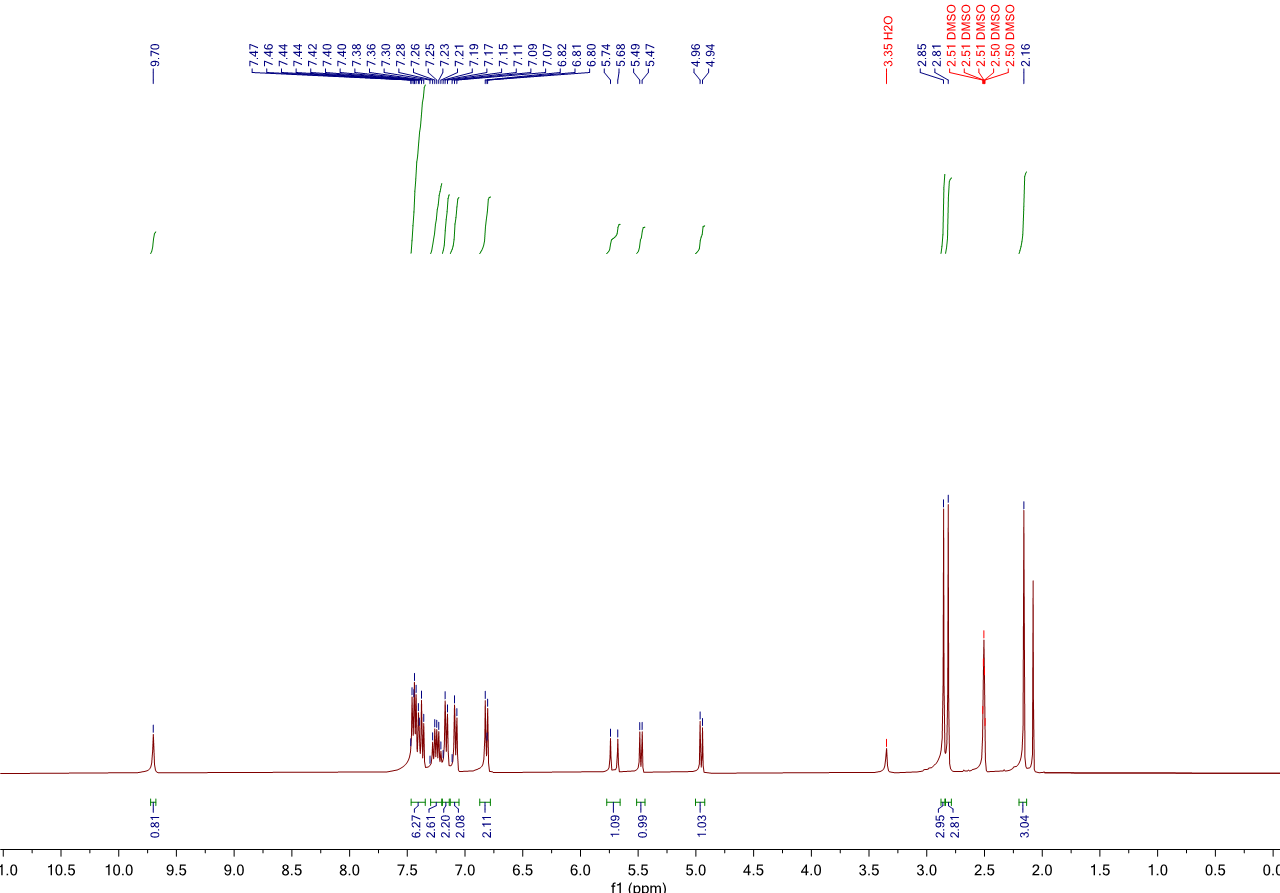


**Figure S29**. ^1^H NMR (400 MHz, DMSO-*d*_6_) of compound (**4e″**)


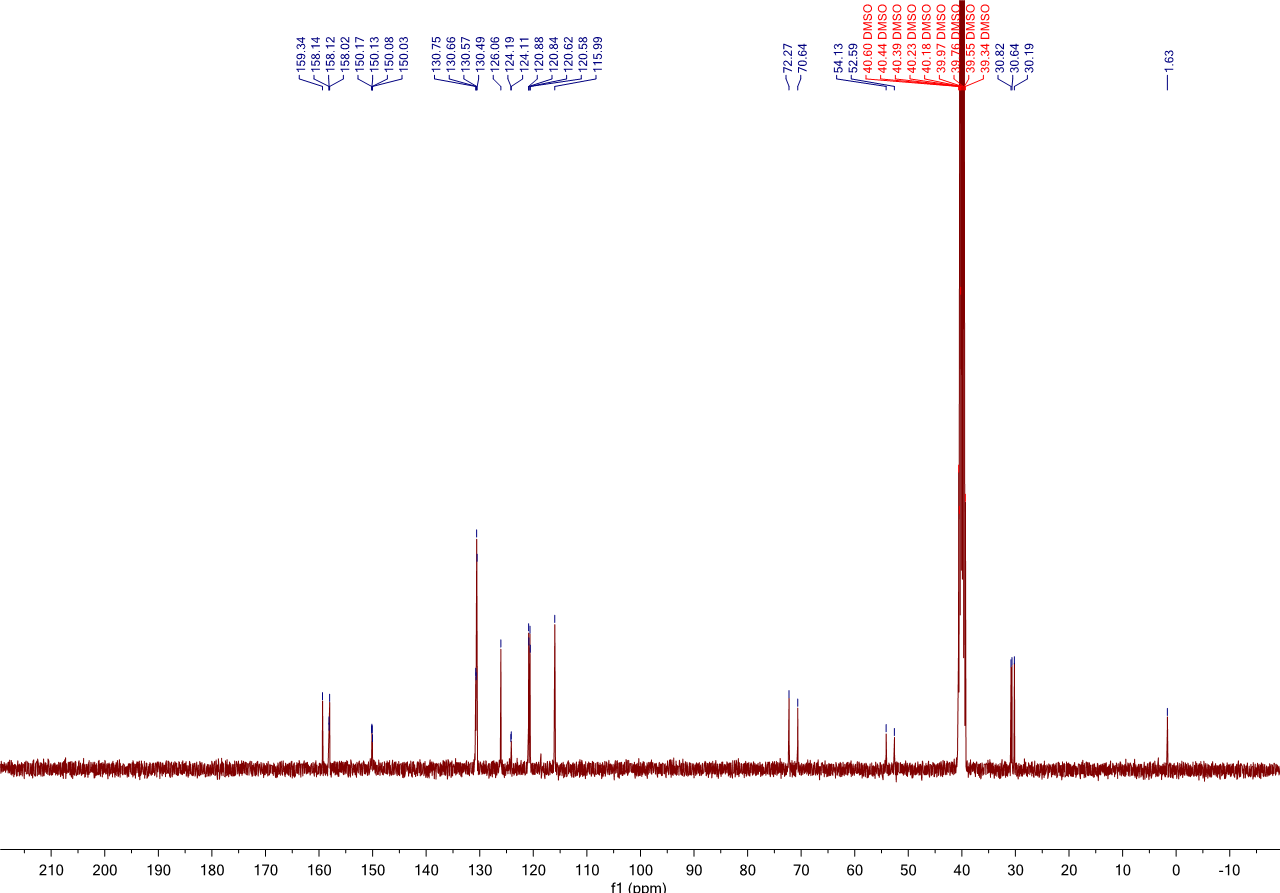


**Figure S30**. ^13^C NMR (101 MHz, DMSO-*d*_6_) of compound (**4e″**)


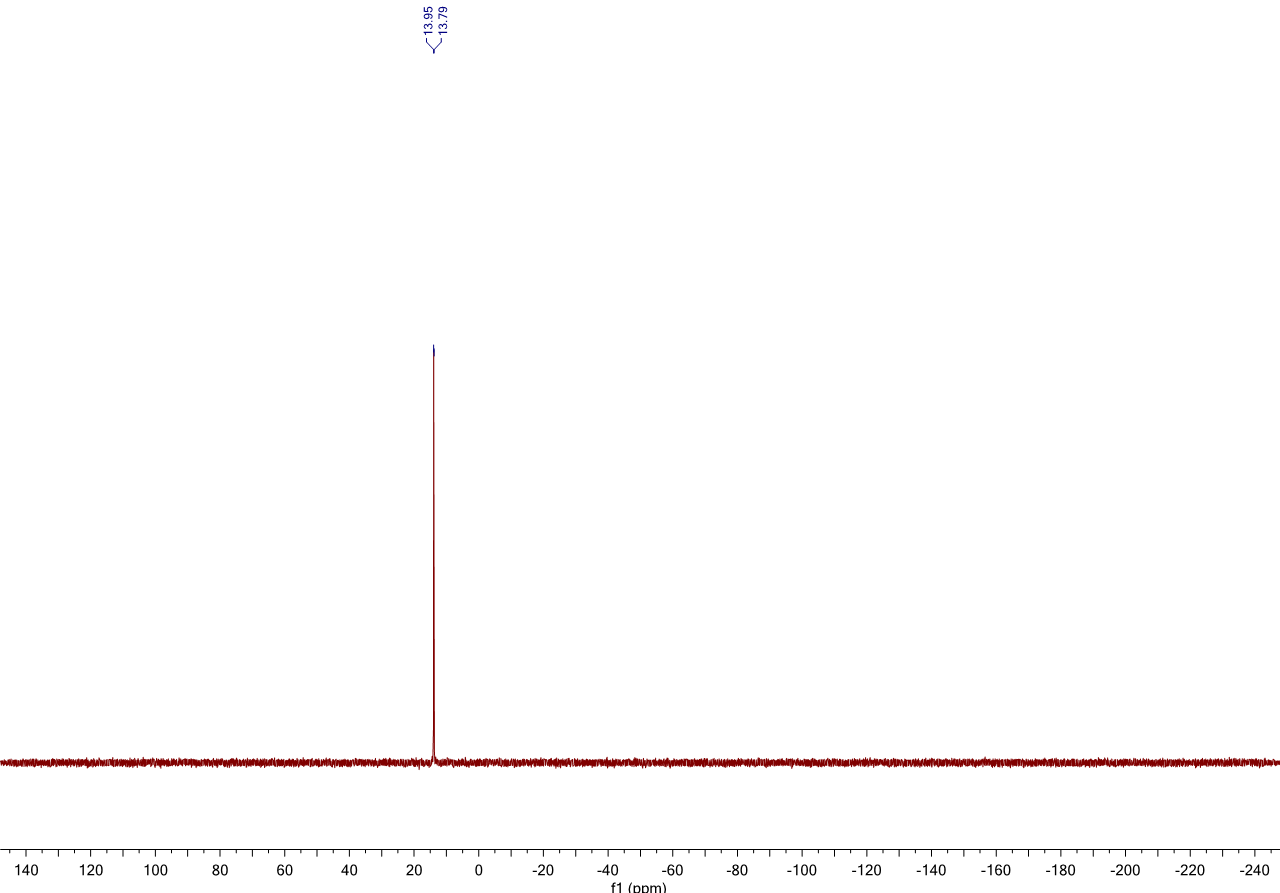


**Figure S31**. ^31^P NMR (162 MHz, DMSO-d6) of compound (**4e″**)


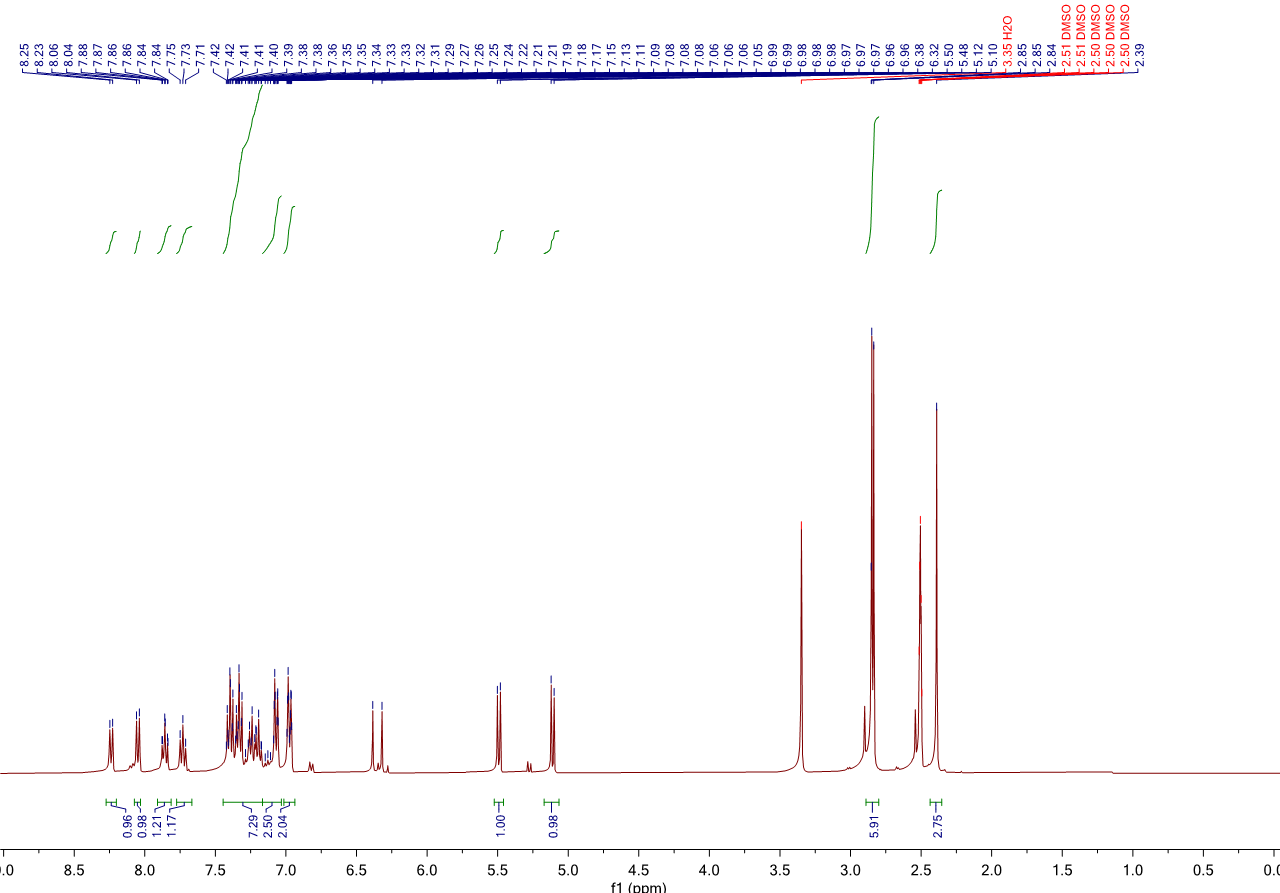


**Figure S32**. ^1^H NMR (400 MHz, DMSO-*d*_6_) of compound (**4f″**)


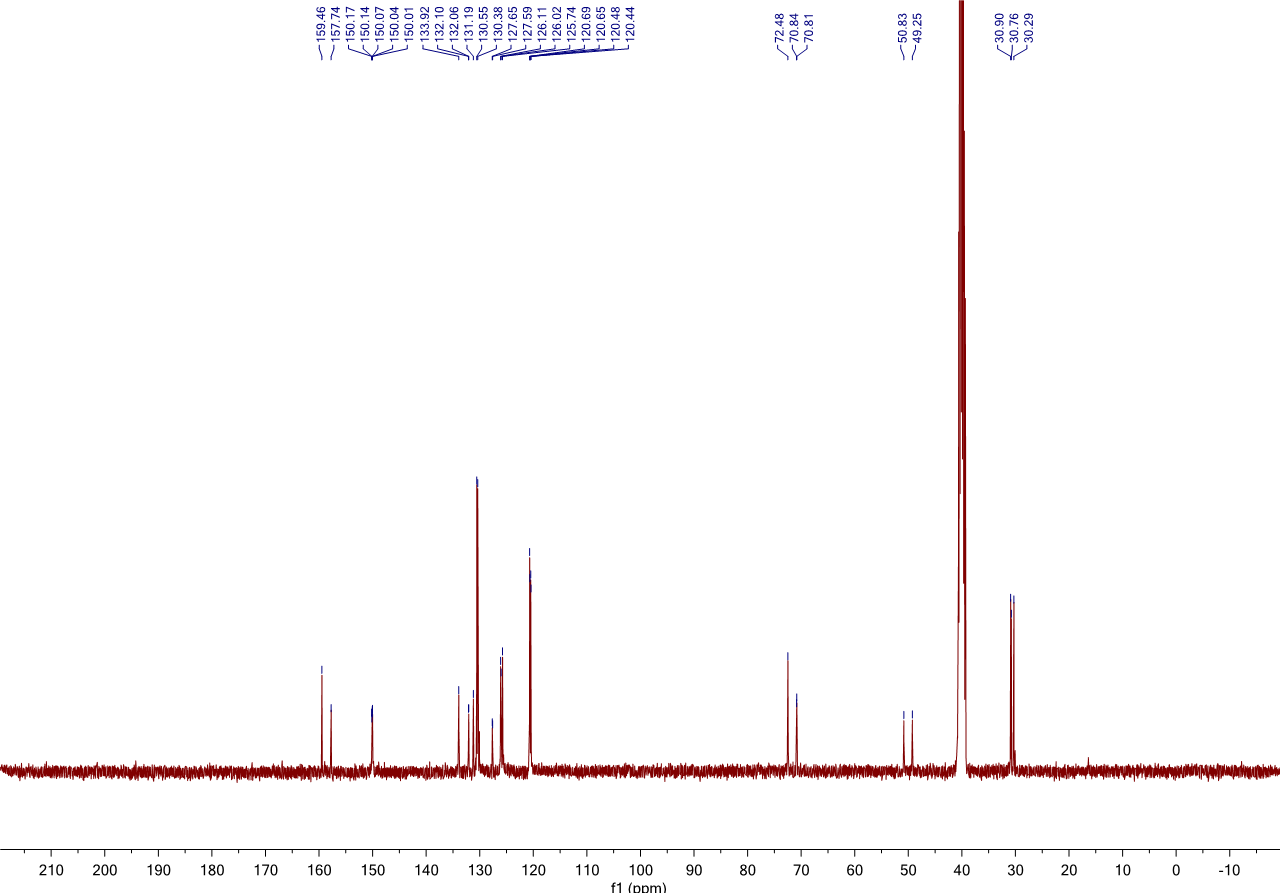


**Figure S33**. ^13^C NMR (101 MHz, DMSO-*d*_6_) of compound (**4f″**)


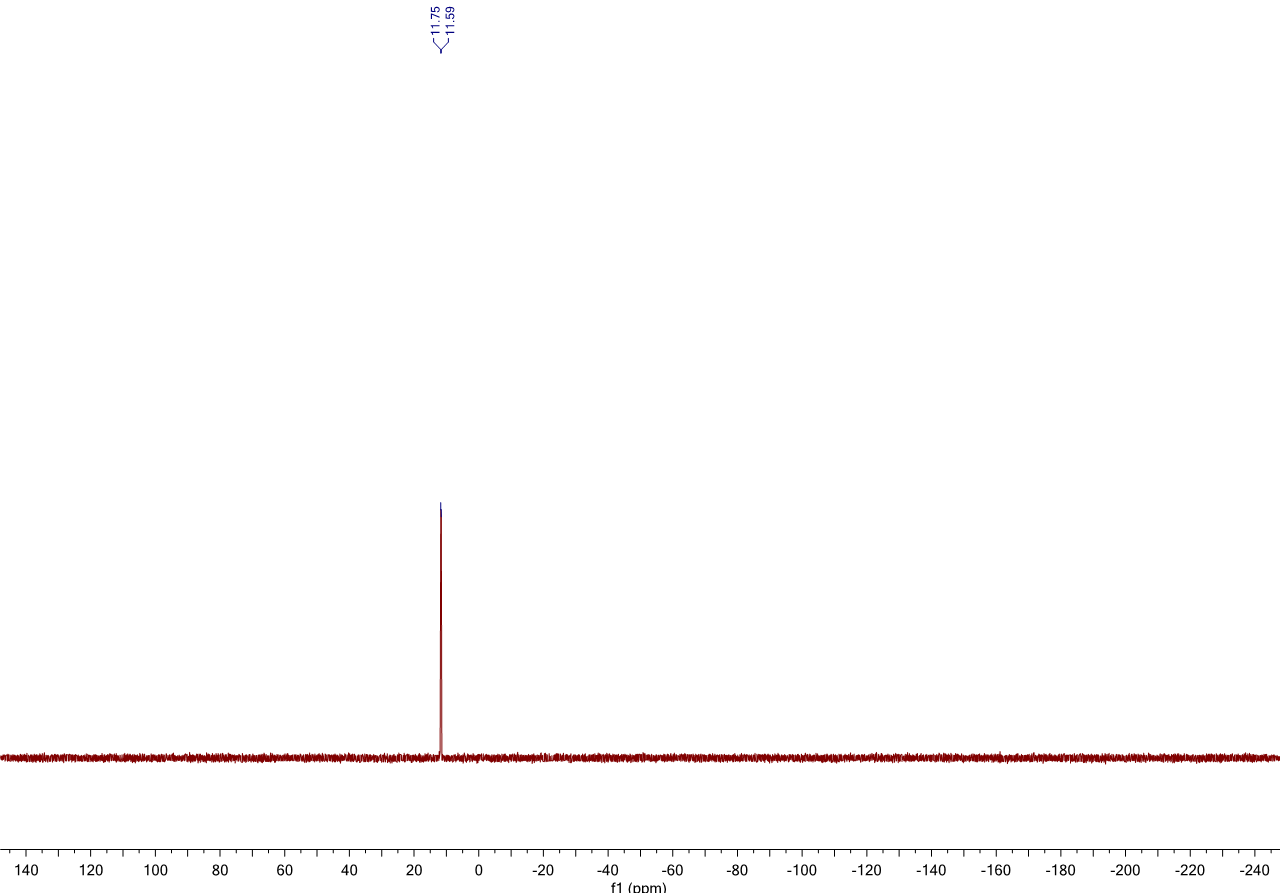


**Figure S34**. ^31^P NMR (162 MHz, DMSO-d6) of compound (**4f″**)


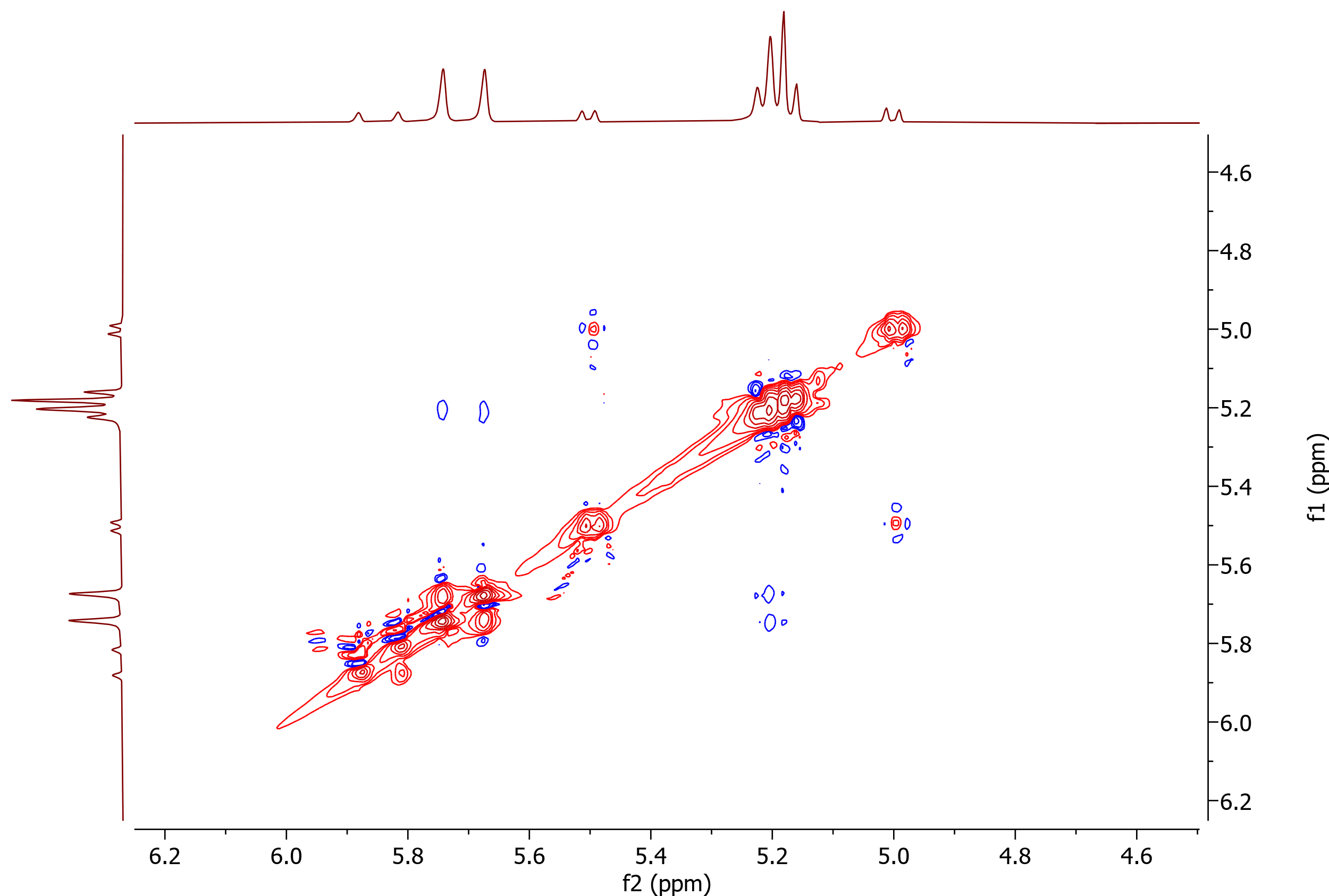


**Figure S35**. 2D NOESY spectrum of compound **4a′**


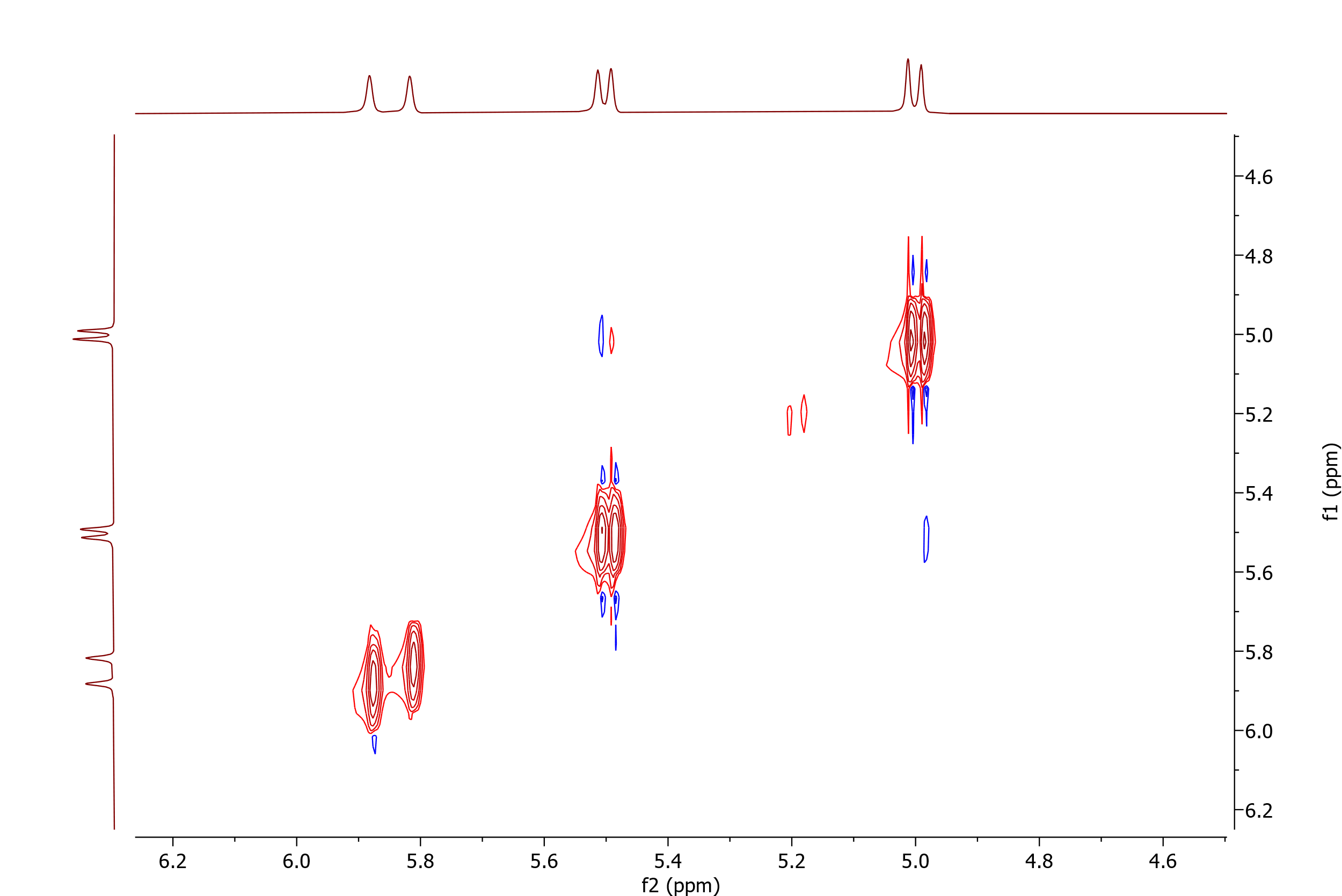


**Figure S36**. 2D NOESY spectrum of compound **4a″**


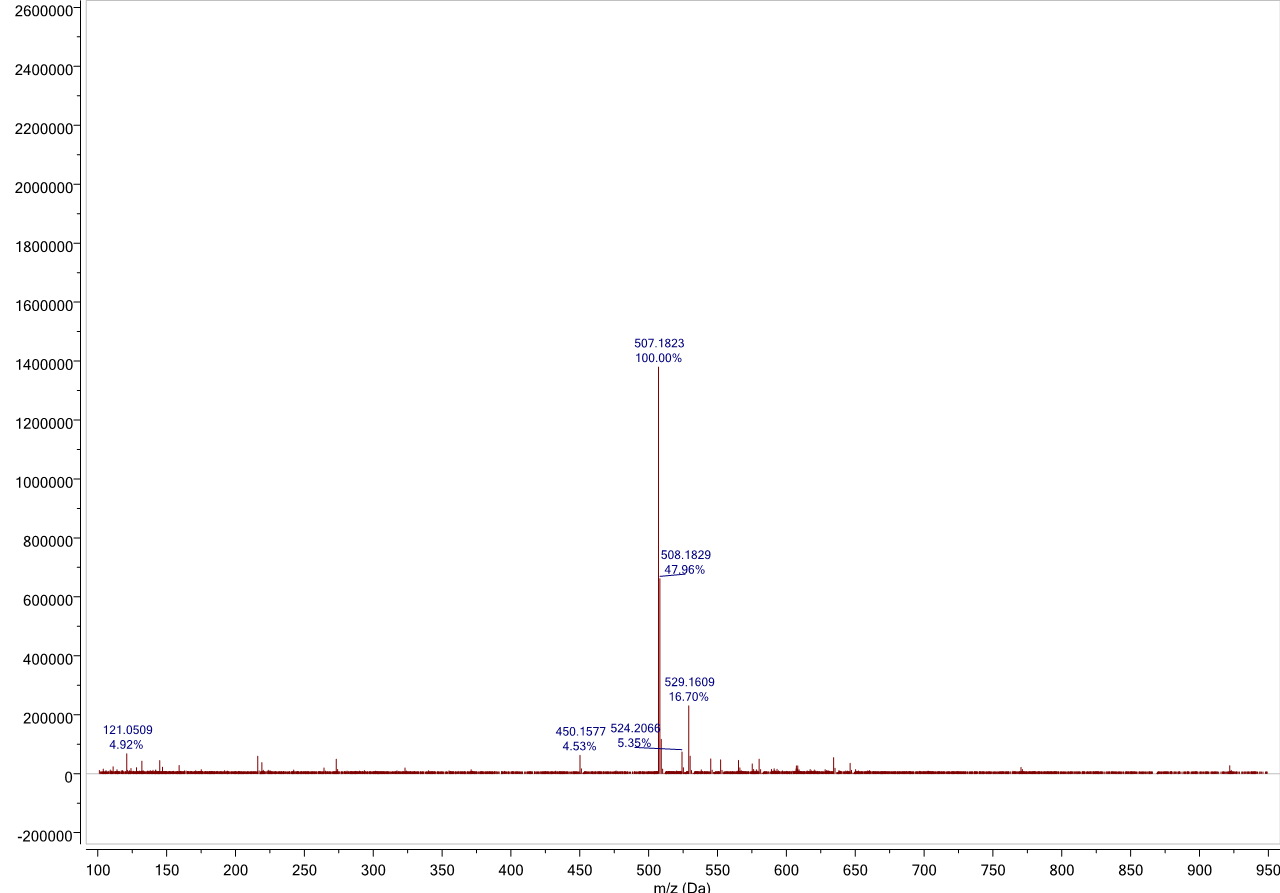


**Figure S37**. HRMS for C_26_H_27_N_4_O_5_P (Mwt.: 506.1719): m/z 507.1823 of **4a′**


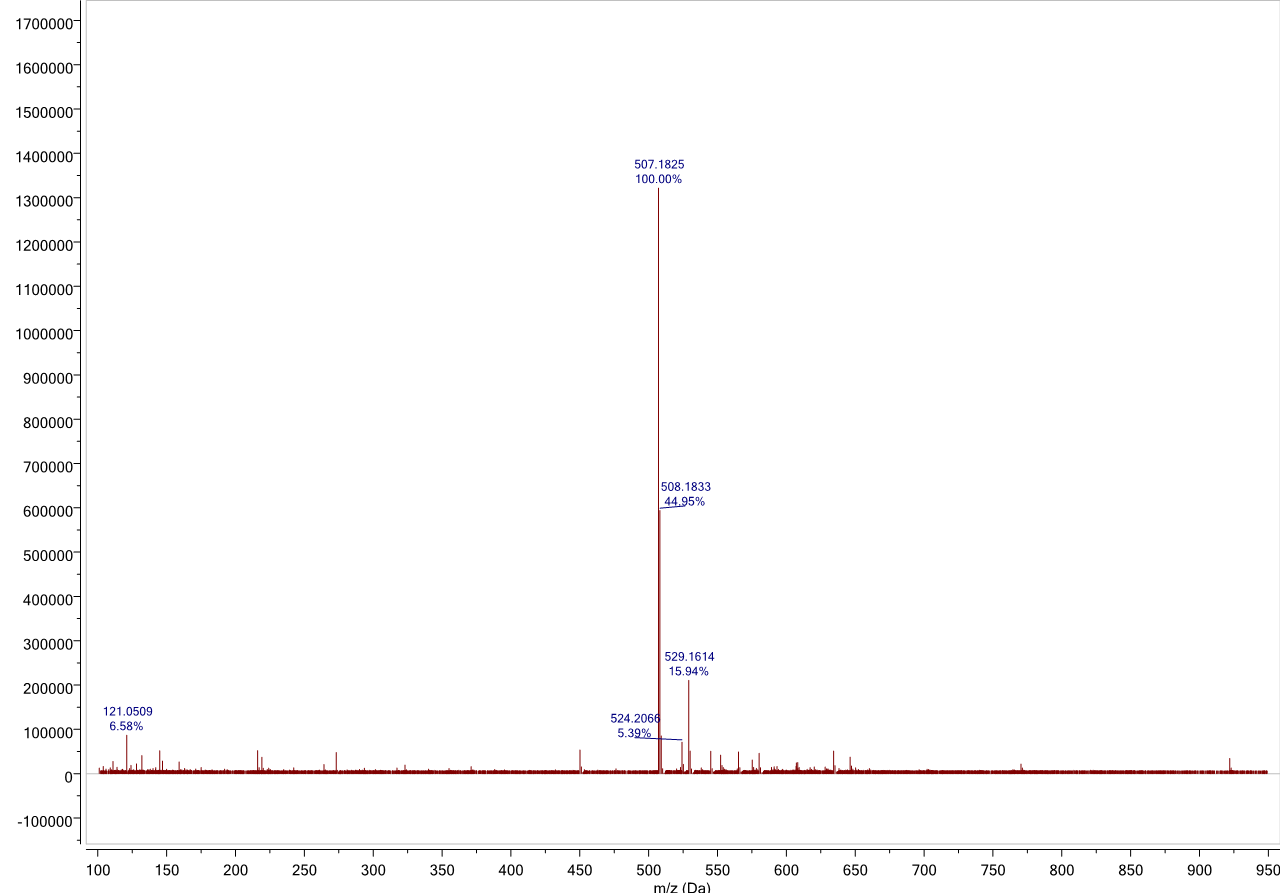


**Figure S38**. HRMS for C_26_H_27_N_4_O_5_P (Mwt.: 506.1719): m/z 507.1825 of **4a″**


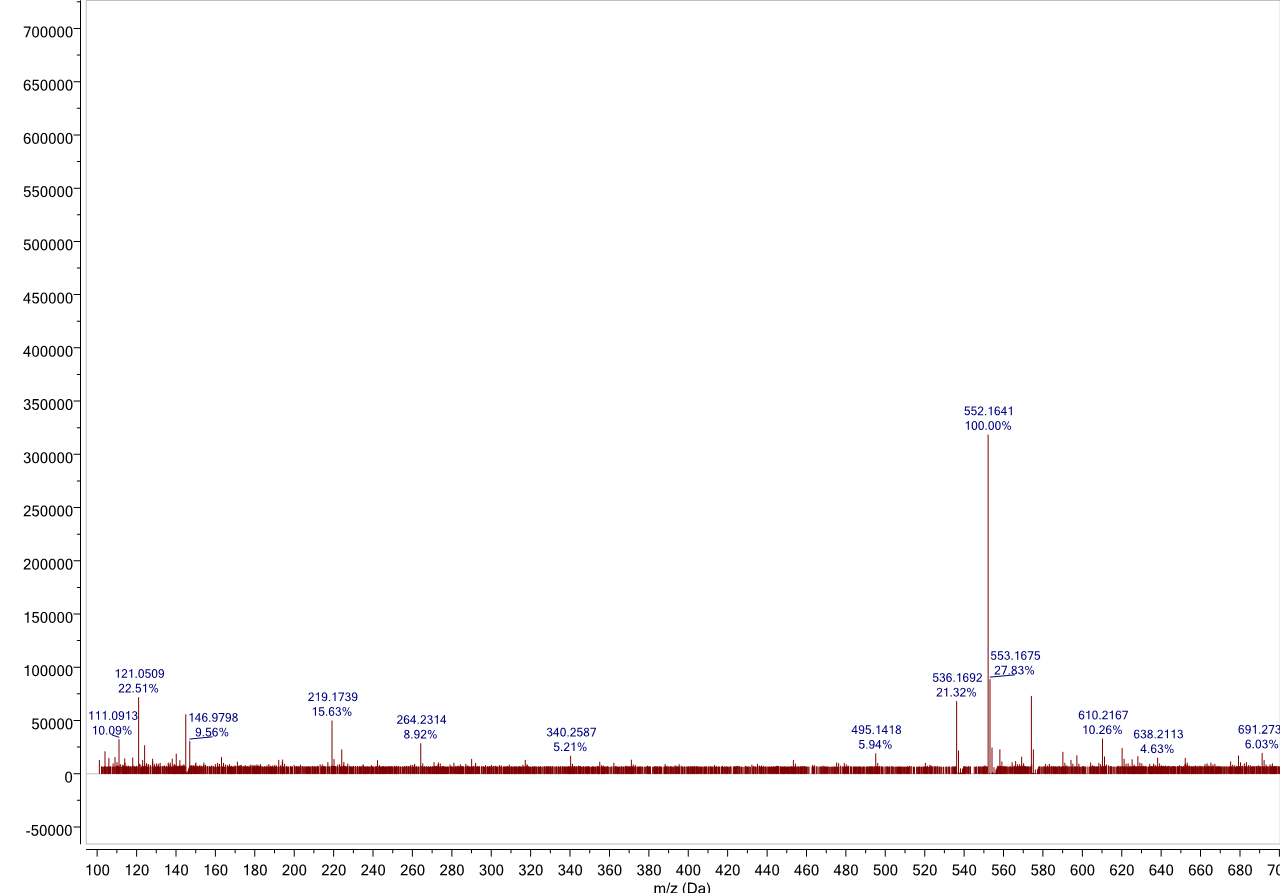


**Figure S39**. HRMS for C_26_H_26_N_5_O_7_P (Mwt.: 551.1570): m/z 552.1641 of **4b′**


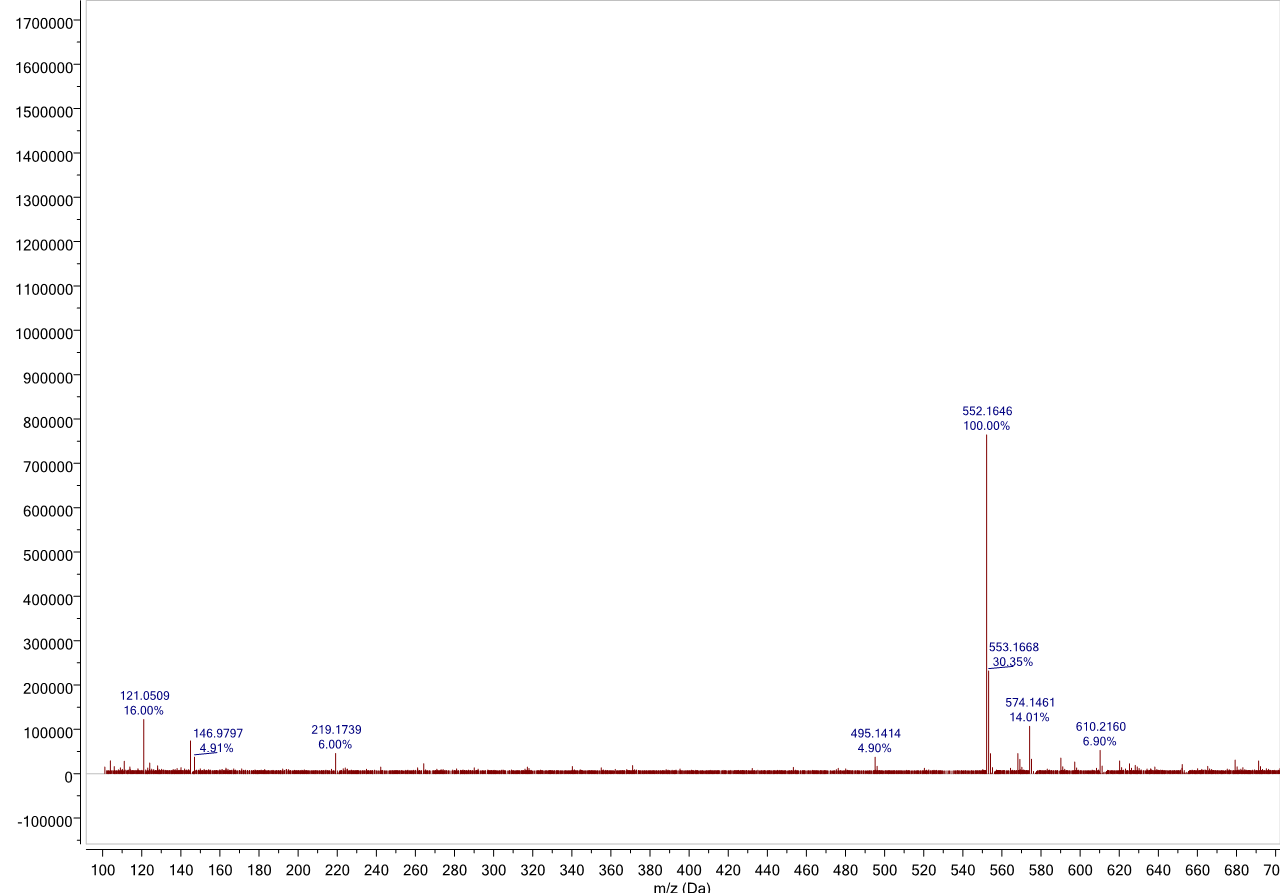


**Figure S40**. HRMS for C_26_H_26_N_5_O_7_P (Mwt.: 551.1570): m/z 552.1646 of **4b″**


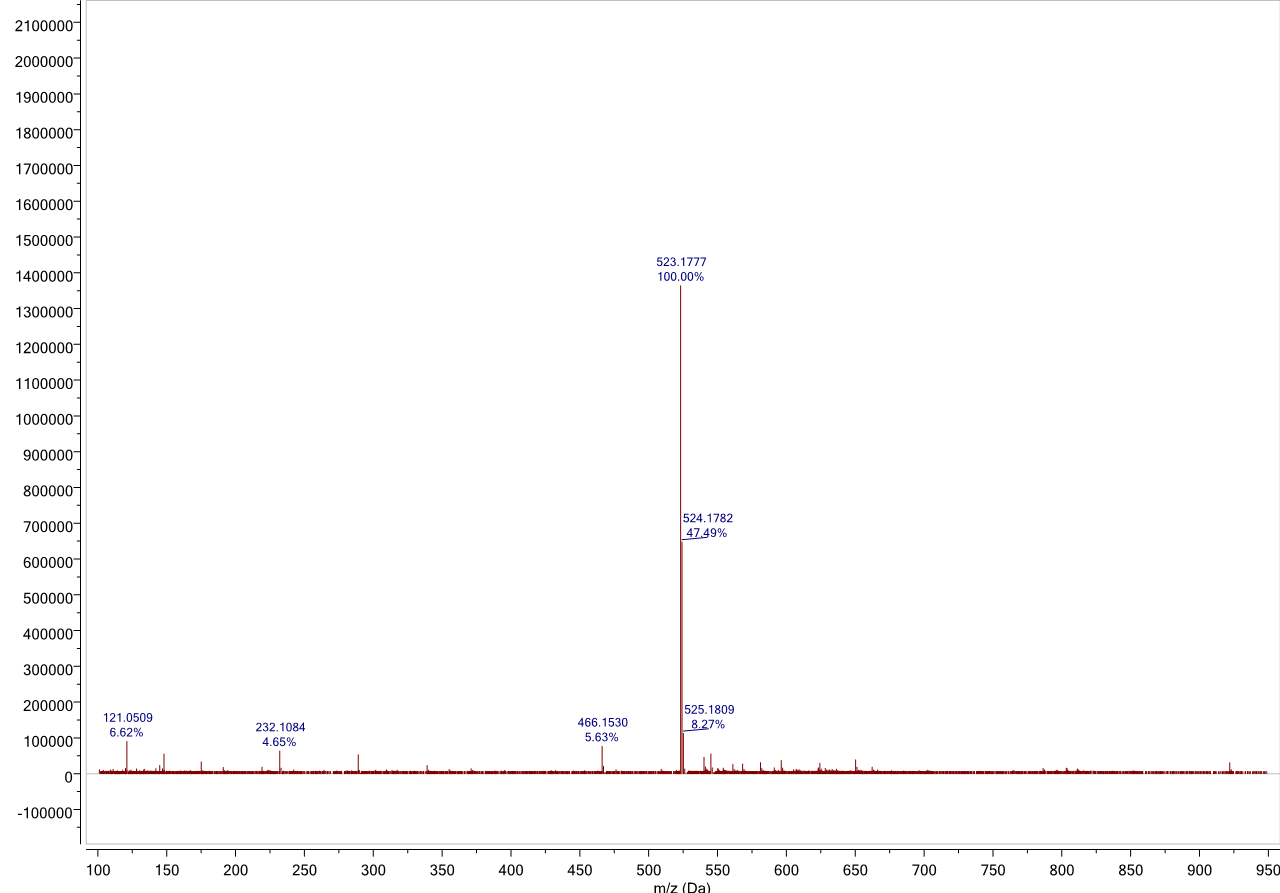


**Figure S41**. HRMS for C_26_H_27_N_4_O_6_P (Mwt.: 522.1668): m/z 523.1777 of **4с′**


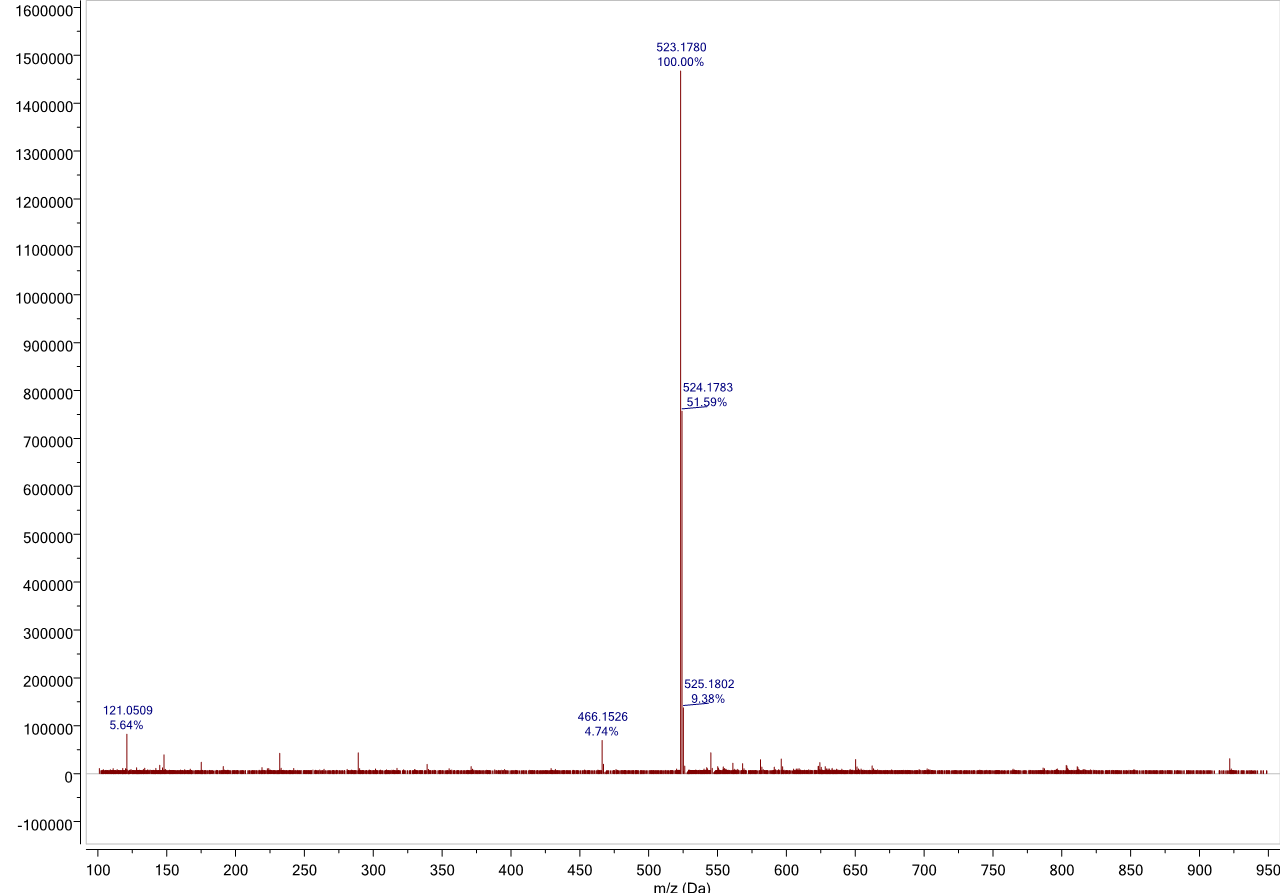


**Figure S42**. HRMS for C_26_H_27_N_4_O_6_P (Mwt.: 522.1668): m/z 523.1780 of **4с″**


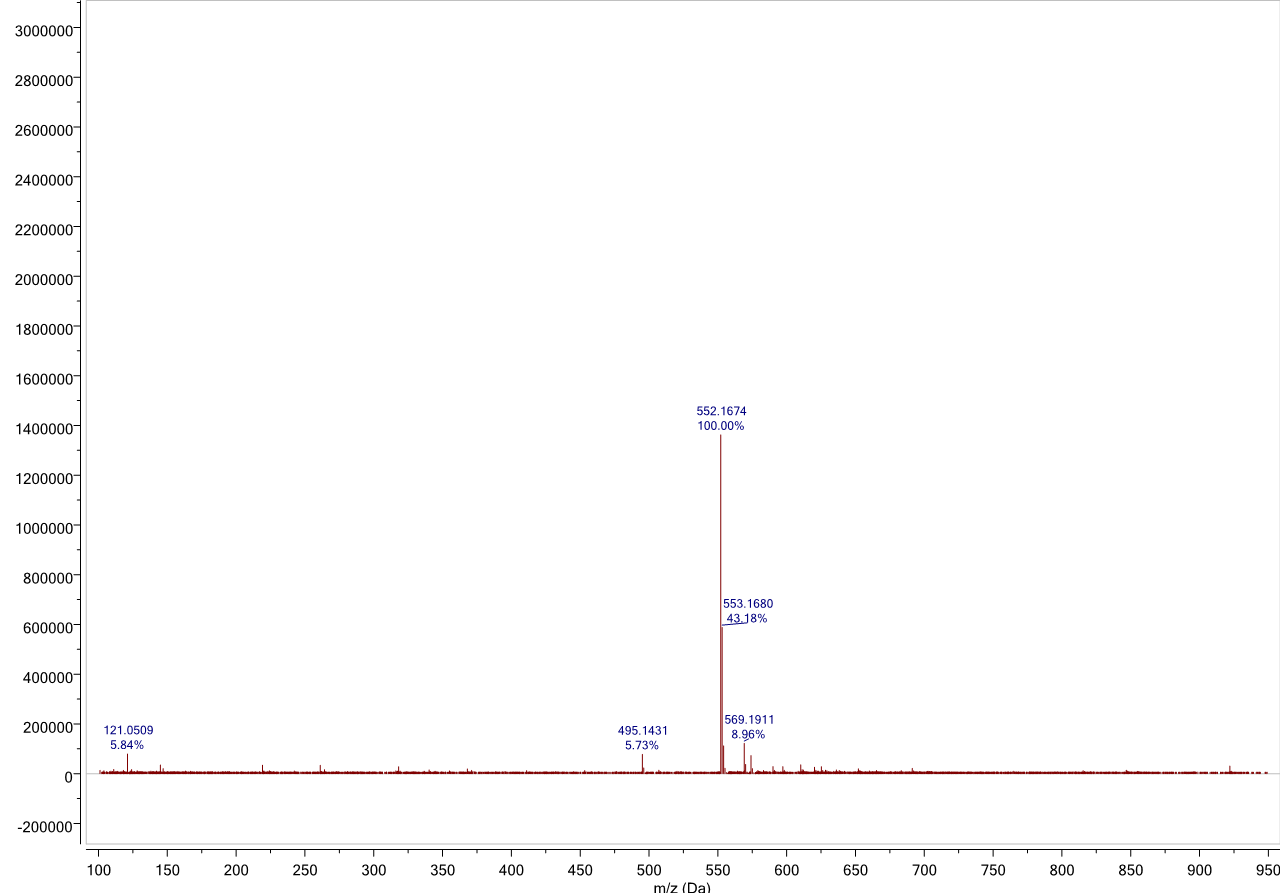


**Figure S43**. HRMS for C_26_H_26_N_5_O_7_P (Mwt.: 551.1570): m/z 552.1674 of **4d′**


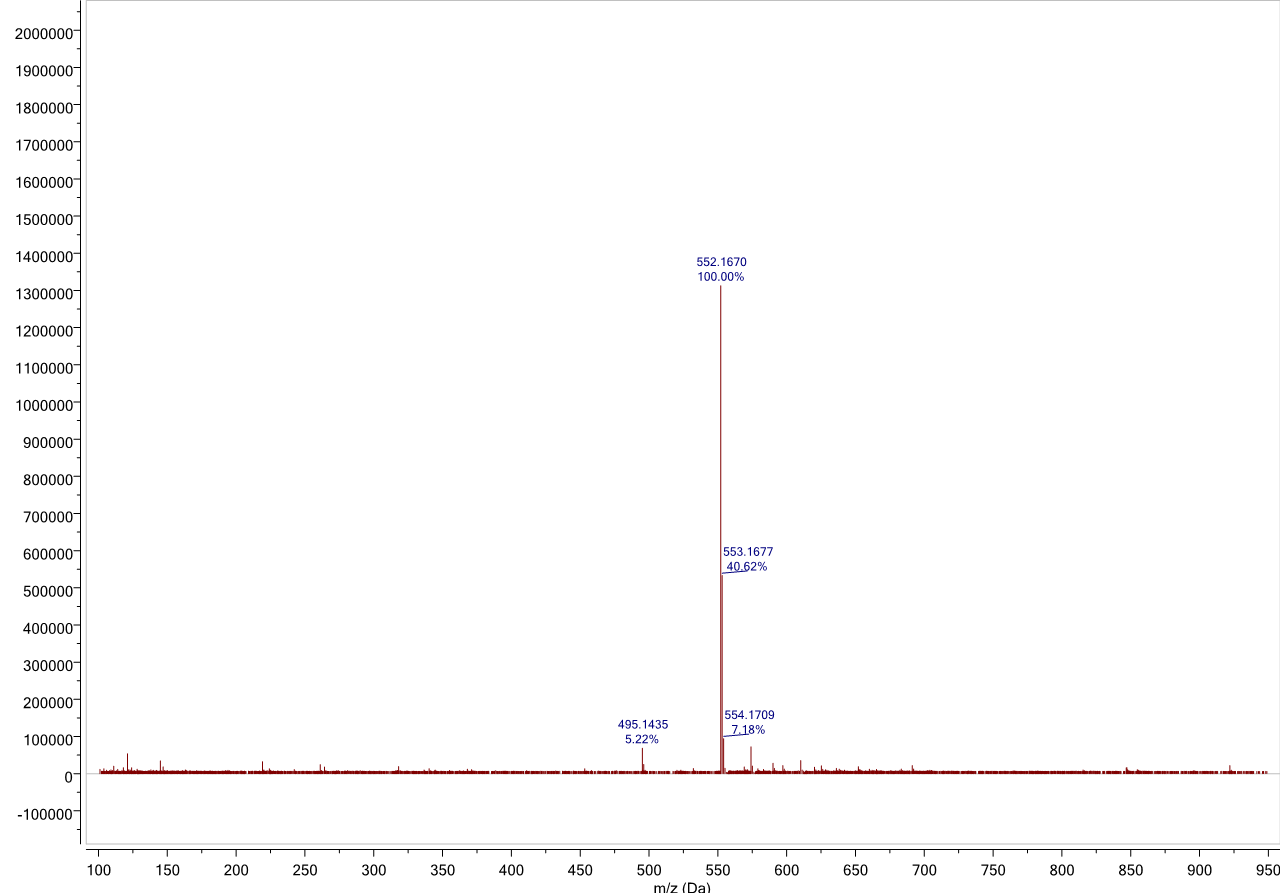


**Figure S44**. HRMS for C_26_H_26_N_5_O_7_P (Mwt.: 551.1570): m/z 552.1670 of **4d″**


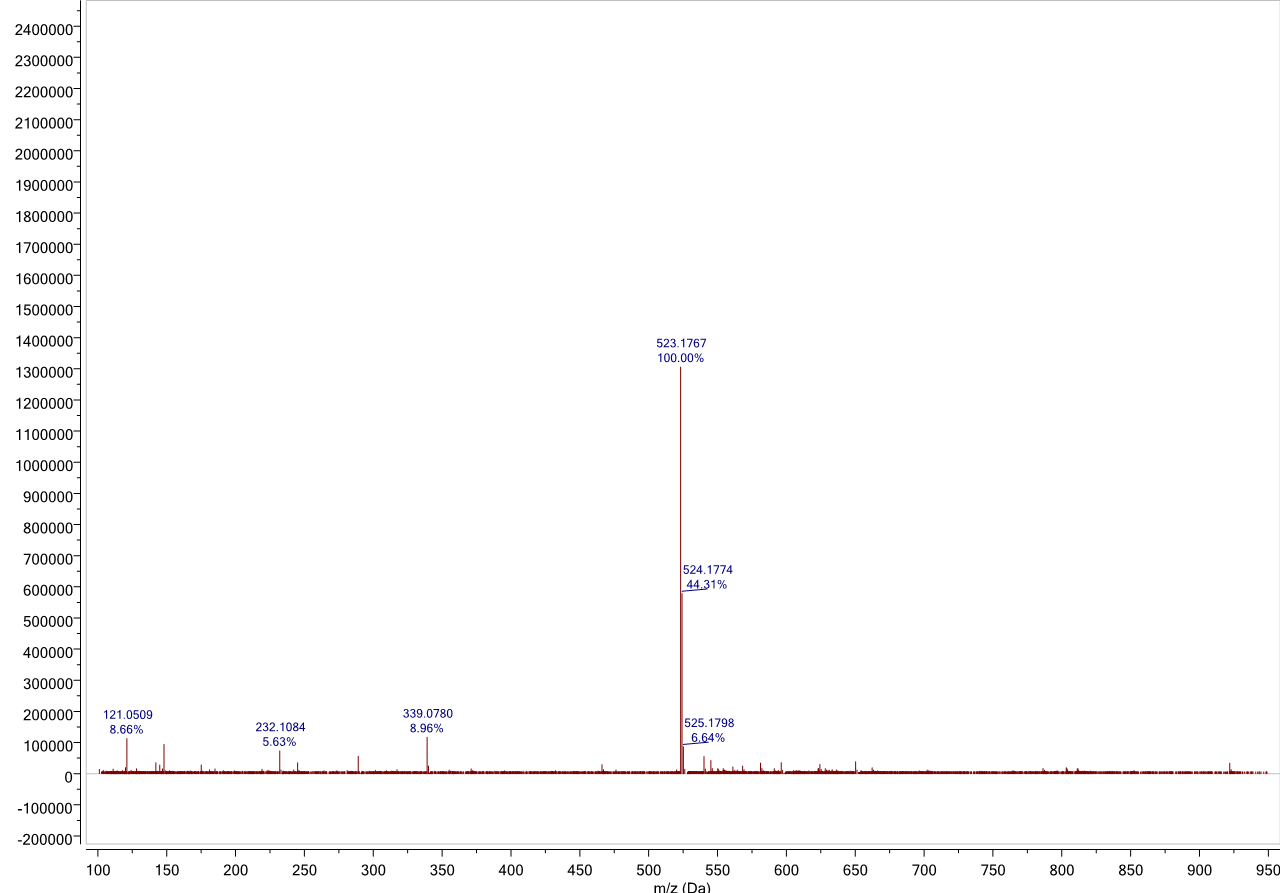


**Figure S45**. HRMS for C_26_H_27_N_4_O_6_P (Mwt.: 522.1668): m/z 523.1767 of **4e′**


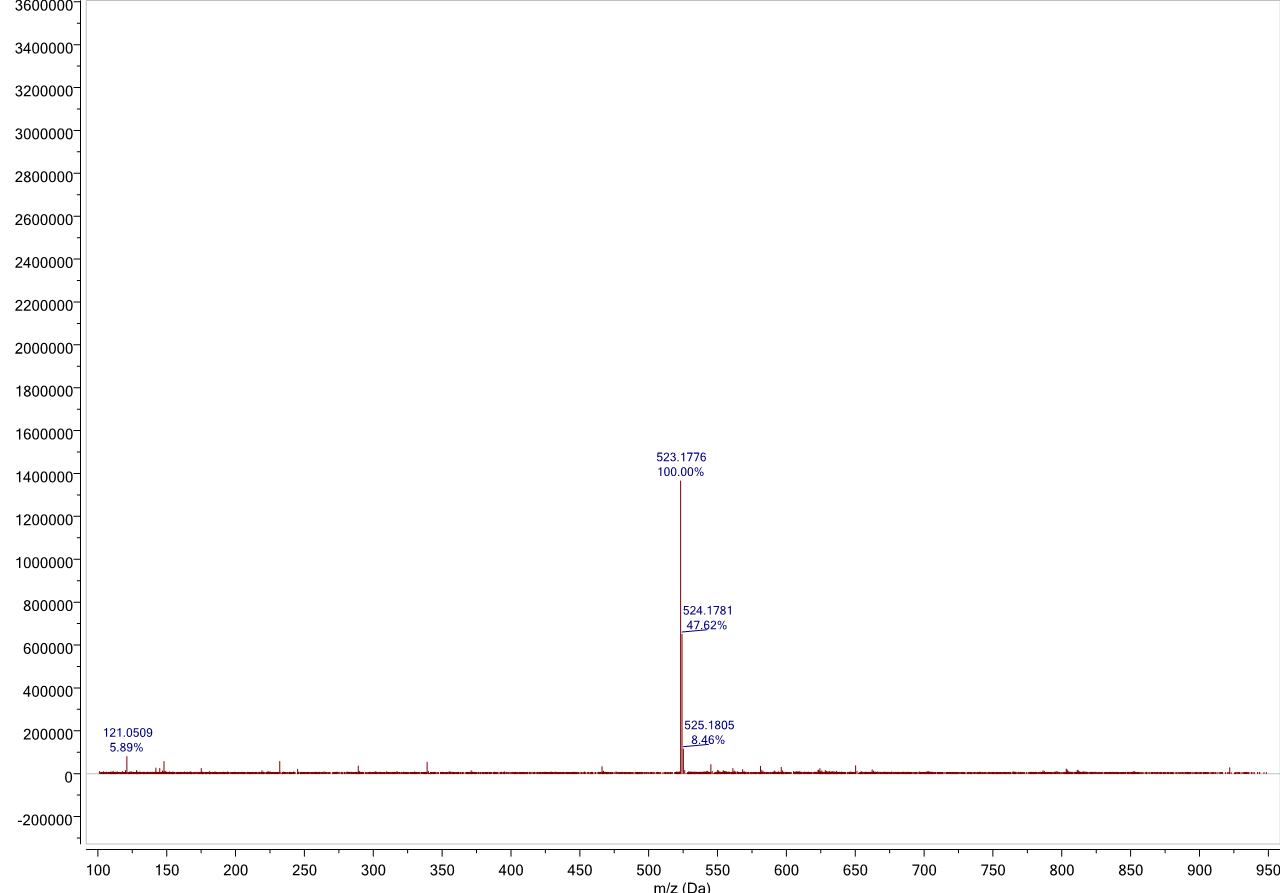


**Figure S46**. HRMS for C_26_H_27_N_4_O_6_P (Mwt.: 522.1668): m/z 523.1776 of **4e″**


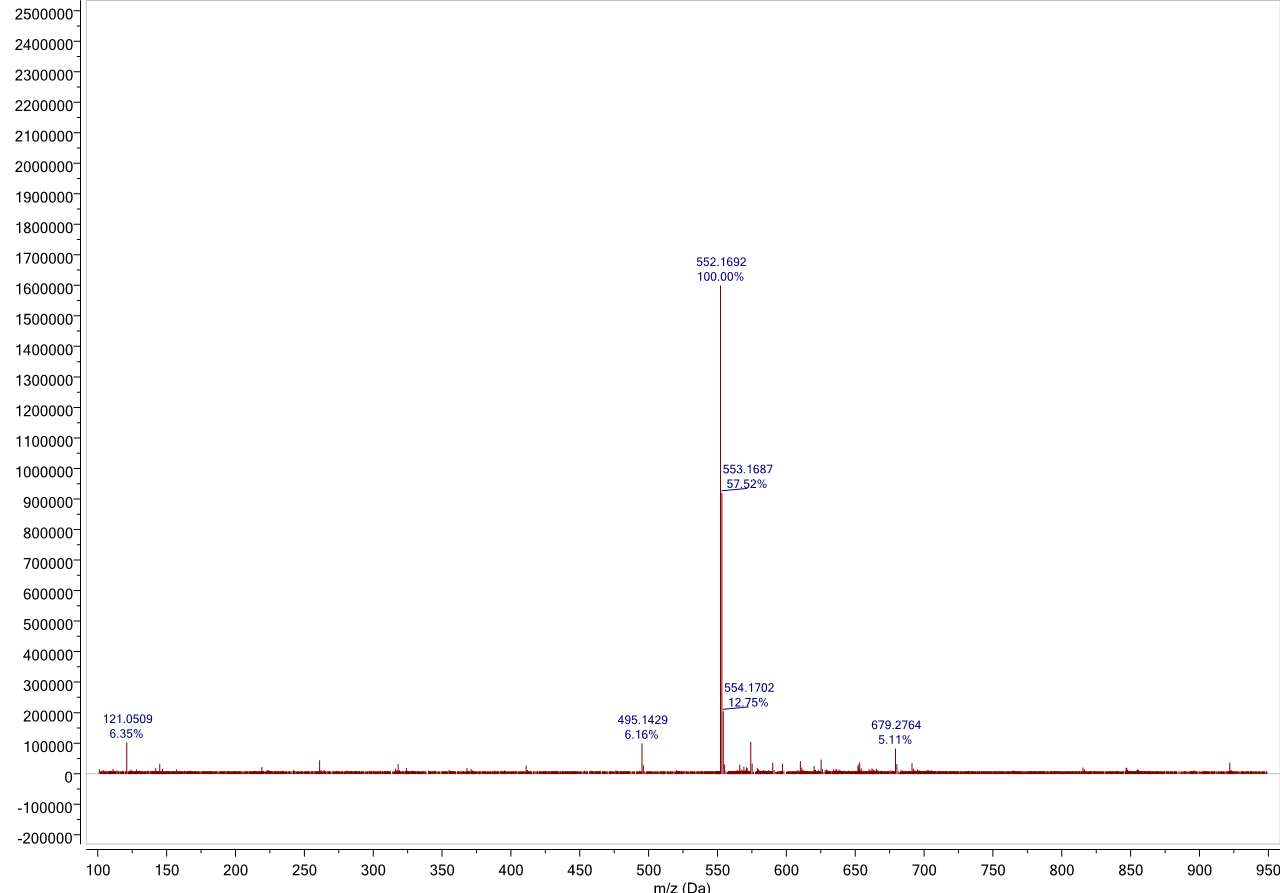


**Figure S47**. HRMS for C_26_H_26_N_5_O_7_P (Mwt.: 551.1570): m/z 552.1692 of **4f″**


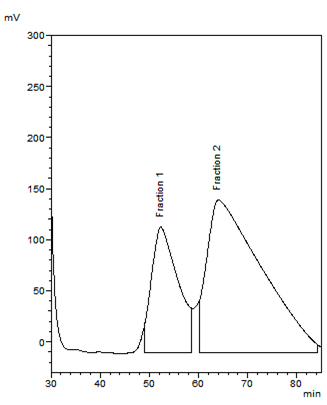


**Figure S48**. Preparative elution profile of **4a**


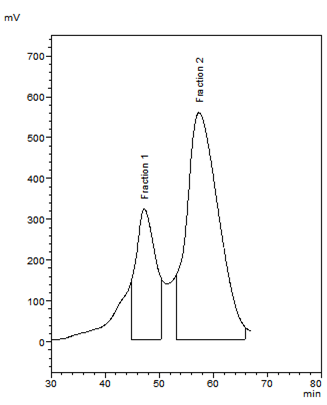


**Figure S49**. Preparative elution profile of **4b**


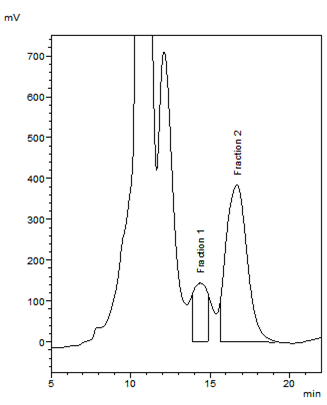


**Figure S50**. Preparative elution profile of **4c**


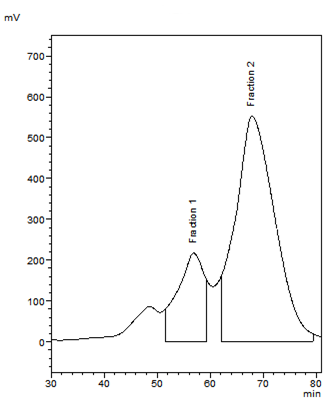


**Figure S51**. Preparative elution profile of **4d**


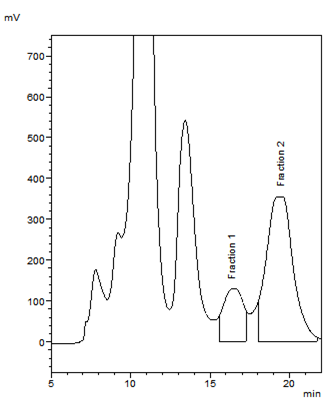


**Figure S52**. Preparative elution profile of **4e**


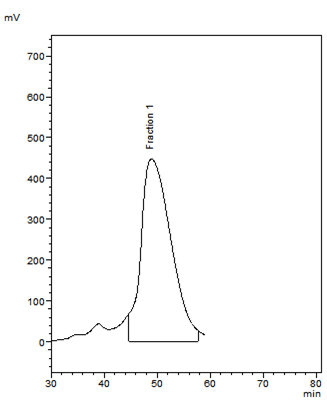


**Figure S53**. Preparative elution profile of **4f**

| **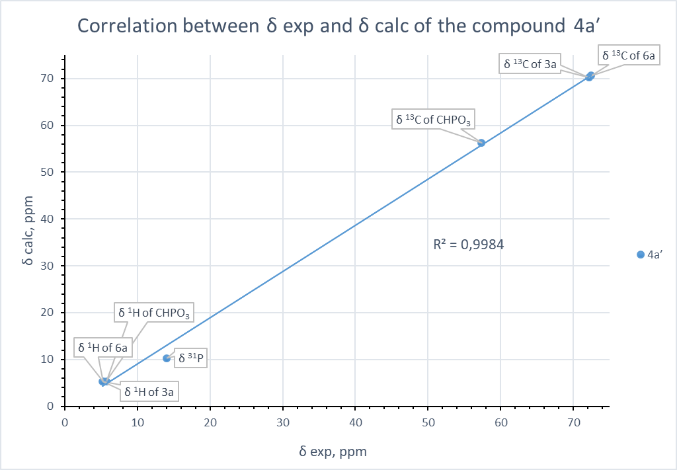** | **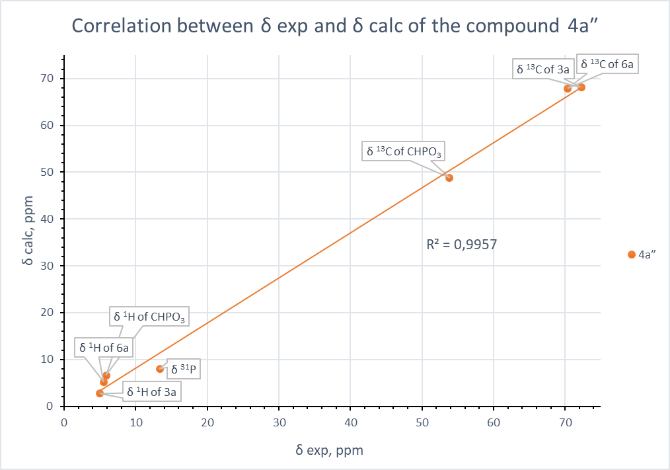** |
| --- | --- |
| **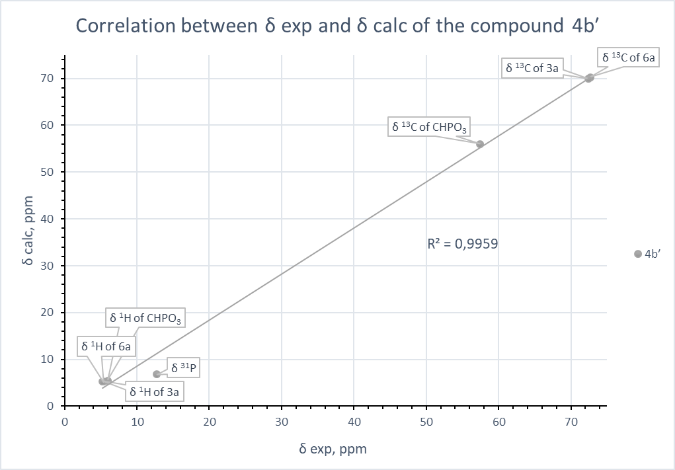** | **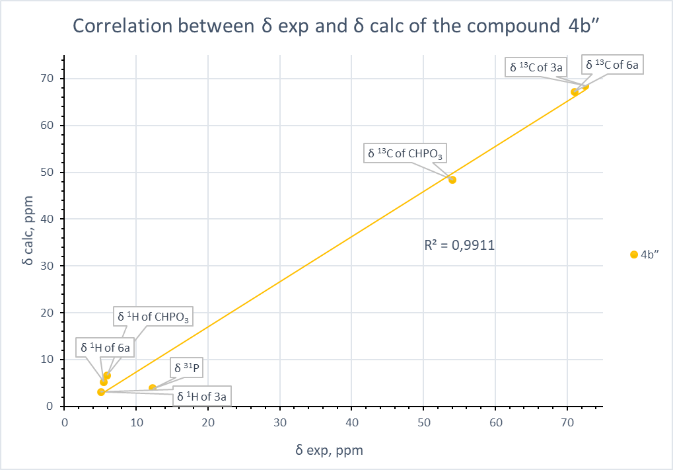** |
| **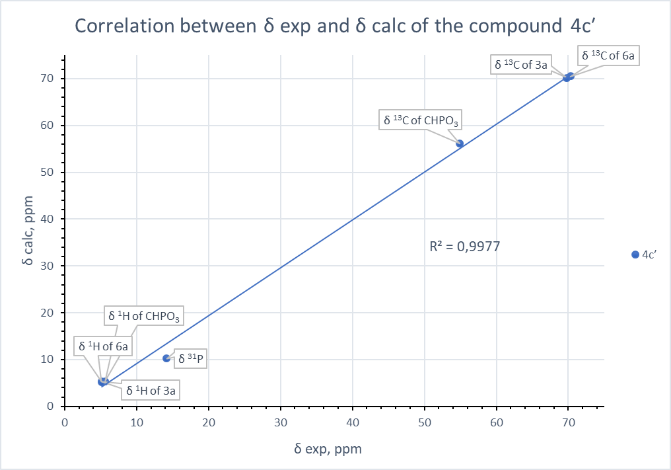** | **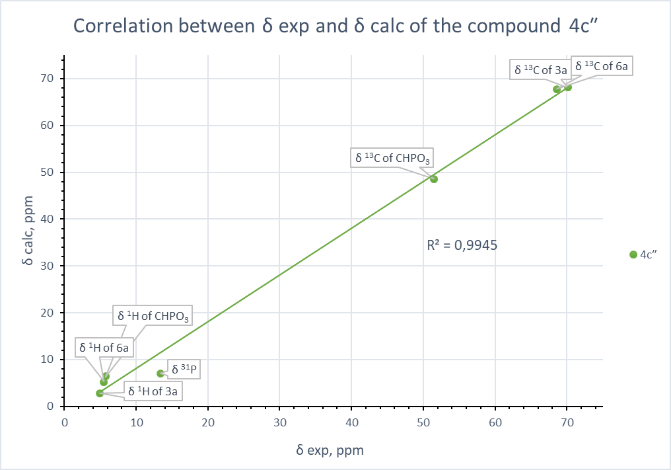** |
| **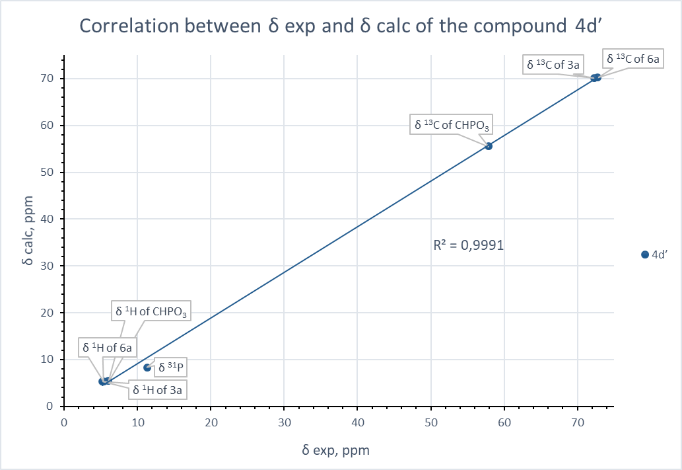** | **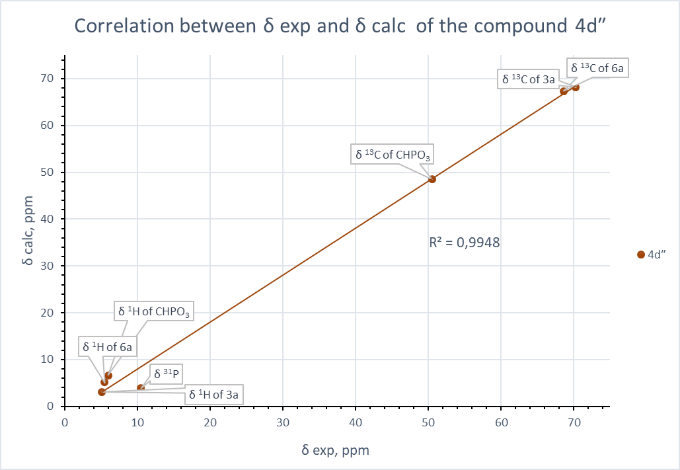** |
| **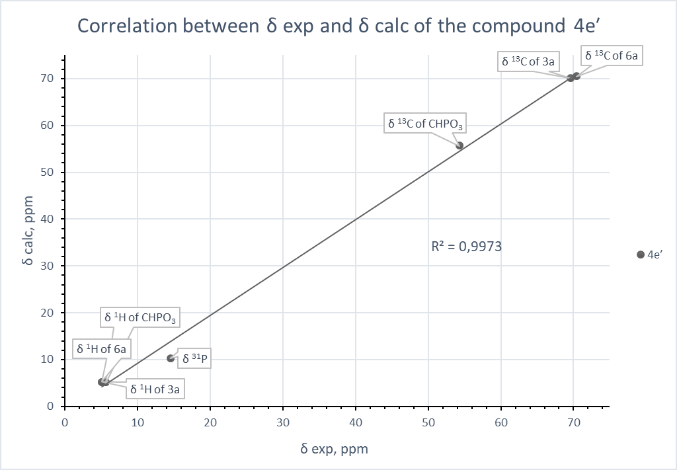** | **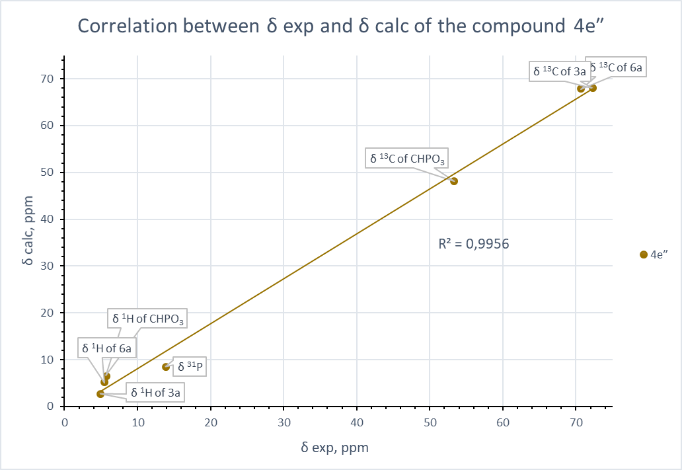** |
|  | **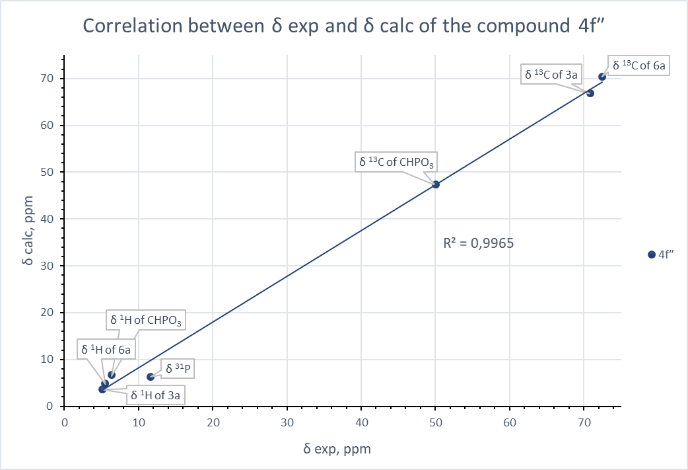** |

**Figure S54. Correlations between experimental and calculation values δ ^1^Н, ^13^С atoms of 3a, 6a, CHPO_3_ and δ ^31^Р**

**Table S1**. Thermodynamic parameters of optimized molecular structures

| Structure | E, Eh | E, kcal/mol | H, Eh | H, kcal/mol |
| --- | --- | --- | --- | --- |
| **4a′** (RSR/SRS) | -1943.50 | -1219564.78 | -1942.96 | -1219226.81 |
| **4a″** (SRR/RSS) | -1943.51 | -1219568.36 | -1942.97 | -1219230.08 |
| **4b′** (RSR/SRS) | -2148.00 | -1347890.67 | -2147.46 | -1347549.35 |
| **4b″** (SRR/RSS) | -2148.01 | -1347894.07 | -2147.46 | -1347552.39 |
| **4c′** (RSR/SRS) | -2018.73 | -1266772.71 | -2018.19 | -1266431.35 |
| **4c″** (SRR/RSS) | -2018.74 | -1266776.20 | -2018.19 | -1266434.29 |
| **4d′** (RSR/SRS) | -2148.00 | -1347890.80 | -2147.46 | -1347549.44 |
| **4d″** (SRR/RSS) | -2148.01 | -1347894.34 | -2147.46 | -1347552.60 |
| **4e′**(RSR/SRS) | -2018.73 | -1266772.84 | -2018.19 | -1266431.31 |
| **4e″** (SRR/RSS) | -2018.74 | -1266776.38 | -2018.19 | -1266434.61 |
| **4f′** (RSR/SRS) | -2148.00 | -1347887.53 | -2147.45 | -1347545.80 |
| **4f″** (SRR/RSS) | -2148.00 | -1347890.38 | -2147.46 | -1347548.71 |

**Table S2**. Thermodynamic parameters of stereoisomer transformation of substances **4а-4f**

| Название | ∆E, kcal/mol | ∆H, kcal/mol |
| --- | --- | --- |
| **4a″→ 4a′** (SRR→RSR/ RSS→SRS) | 3.58 | 3.26 |
| **4b″→ 4b′** (SRR**→**RSR/RSS**→**SRS) | 3.40 | 3.03 |
| **4c″→ 4c′** (SRR**→**RSR/RSS**→**SRS) | 3.49 | 2.94 |
| **4d″→ 4d′** (SRR**→**RSR/RSS**→**SRS) | 3.55 | 3.16 |
| **4e″→ 4e′** (SRR**→**RSR/RSS**→**SRS) | 3.54 | 3.30 |
| **4f″→ 4f′** (SRR**→**RSR/RSS**→**SRS) | 2.85 | 2.92 |

**Table S3**. Comparison of geometric parameters of **4a′ (**RSR) optimized molecular structure and experimental data.

| Parameter | **4a′ (**RSR) Experiment data | **4a′ (**RSR) calculation data  m062x/6-311+G(2d,p) | δ, %  Relative error |
| --- | --- | --- | --- |
| 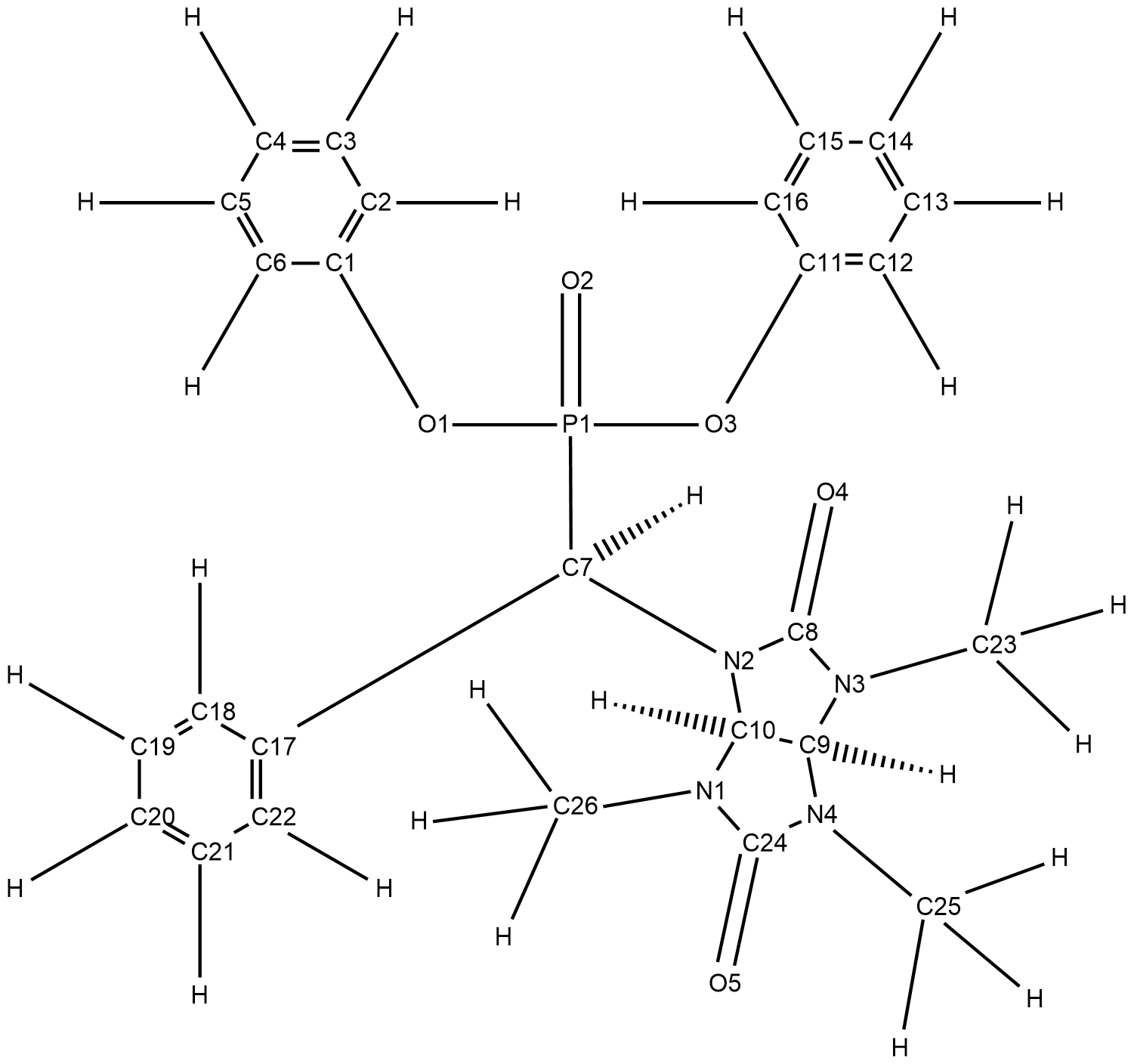 | | | |
| Bond length, A | | | |
| C7-P1 | 1.83 | 1.84 | 0.25 |
| C7-C17 | 1.52 | 1.52 | 0.38 |
| C7-N2 | 1.46 | 1.44 | 0.99 |
| C7-H | 1.00 | 1.10 | 9.51 |
| P1-O3 | 1.59 | 1.60 | 0.91 |
| P1-O1 | 1.60 | 1.61 | 0.92 |
| P1=O2 | 1.46 | 1.47 | 0.02 |
| C17-C22 | 1.40 | 1.39 | 0.33 |
| C22-H | 0.95 | 1.08 | 14.08 |
| N2-C8 | 1.37 | 1.37 | 0.21 |
| N2-C10 | 1.46 | 1.45 | 0.67 |
| C10-H | 1.00 | 1.09 | 9.27 |
| C10-N1 | 1.44 | 1.45 | 0.40 |
| C8=O4 | 1.23 | 1.22 | 0.71 |
| N1-C26 | 1.45 | 1.45 | 0.25 |
| Angles, ̊ | | | |
| C7-P1=O2 | 120.81 | 120.97 | 0.13 |
| C7-P1=O3 | 100.89 | 102.11 | 1.20 |
| C7-P1=O1 | 97.89 | 98.31 | 0.43 |
| C7-N2-C8 | 125.97 | 125.24 | 0.58 |
| C7-N2-C10 | 120.78 | 122.07 | 1.07 |
| C7-C17=C22 | 120.58 | 120.69 | 0.09 |
| C17=C22=C21 | 120.02 | 119.98 | 0.03 |

**Table S4**. Comparison of geometric parameters of **4a′ (**SRS) optimized molecular structure and experimental data.

| Parameter | **4a′ (**SRS) Experiment data | **4a′ (**SRS) calculation data  m062x/6-311+G(2d,p) | δ, %  Relative error |
| --- | --- | --- | --- |
| 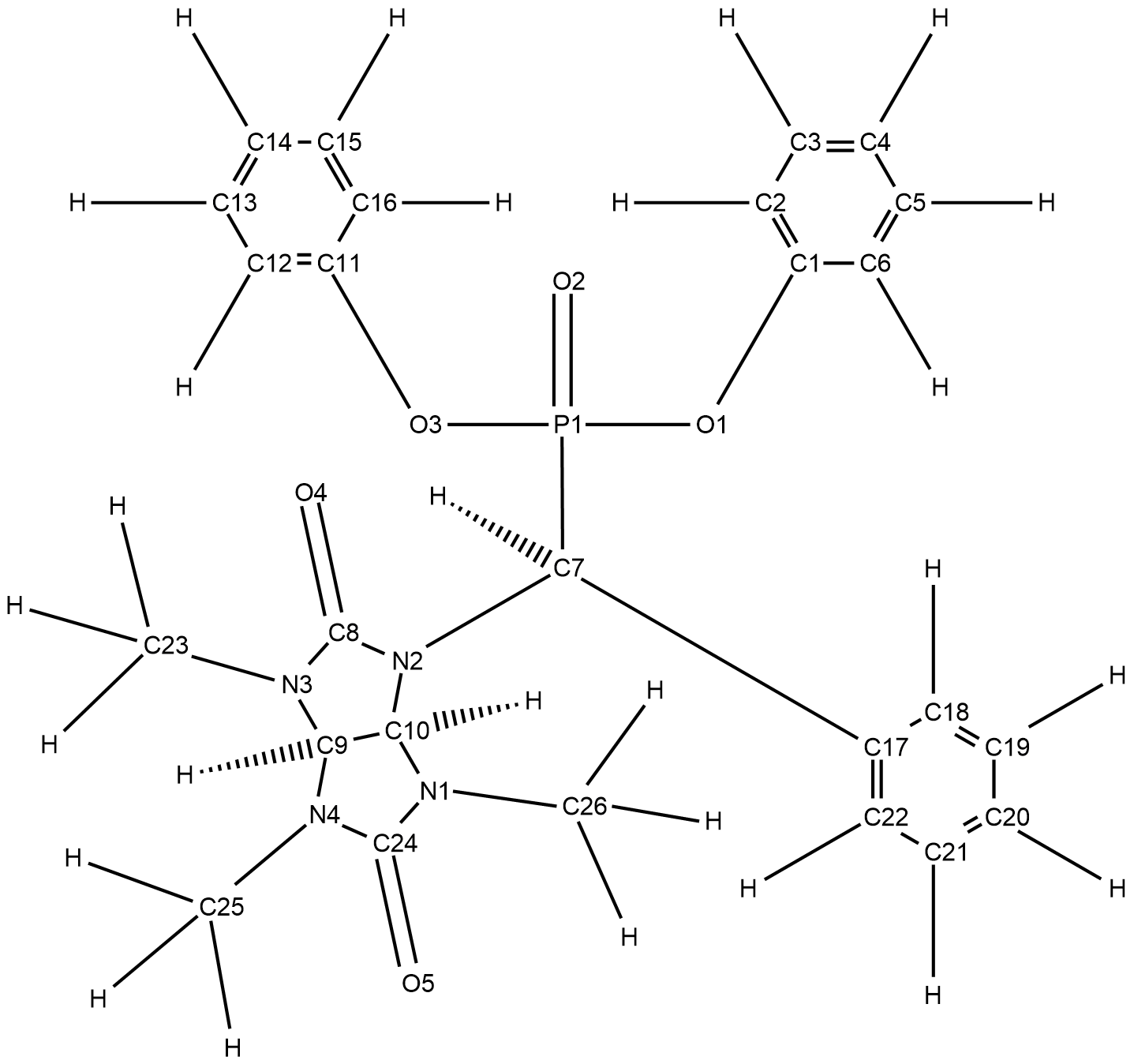 | | | |
| Bond length, A | | | |
| C7-P1 | 1.83 | 1.84 | 0.25 |
| C7-C17 | 1.52 | 1.52 | 0.38 |
| C7-N2 | 1.46 | 1.44 | 0.99 |
| C7-H | 1.00 | 1.10 | 9.51 |
| P1-O3 | 1.59 | 1.60 | 0.91 |
| P1-O1 | 1.60 | 1.61 | 0.92 |
| P1=O2 | 1.46 | 1.47 | 0.02 |
| C17-C22 | 1.40 | 1.39 | 0.33 |
| C22-H | 0.95 | 1.08 | 14.08 |
| N2-C8 | 1.37 | 1.37 | 0.21 |
| N2-C10 | 1.46 | 1.45 | 0.67 |
| C10-H | 1.00 | 1.09 | 9.27 |
| C10-N1 | 1.44 | 1.45 | 0.40 |
| C8=O4 | 1.23 | 1.22 | 0.71 |
| N1-C26 | 1.45 | 1.45 | 0.25 |
| Angles, ° | | | |
| C7-P1=O2 | 120.81 | 120.97 | 0.13 |
| C7-P1=O3 | 100.89 | 102.11 | 1.20 |
| C7-P1=O1 | 97.89 | 98.31 | 0.43 |
| C7-N2-C8 | 125.97 | 125.24 | 0.58 |
| C7-N2-C10 | 120.78 | 122.07 | 1.07 |
| C7-C17=C22 | 120.58 | 120.69 | 0.09 |
| C17=C22=C21 | 120.02 | 119.98 | 0.03 |

**Table S5**. Comparison of geometric parameters of **4a″ (**SRR) optimized molecular structure and experimental data.

| Parameter | **4a″ (**SRR)  Experiment data | **4a″ (**SRR) calculation data  m062x/6-311+G(2d,p) | δ, %  Relative error |
| --- | --- | --- | --- |
| 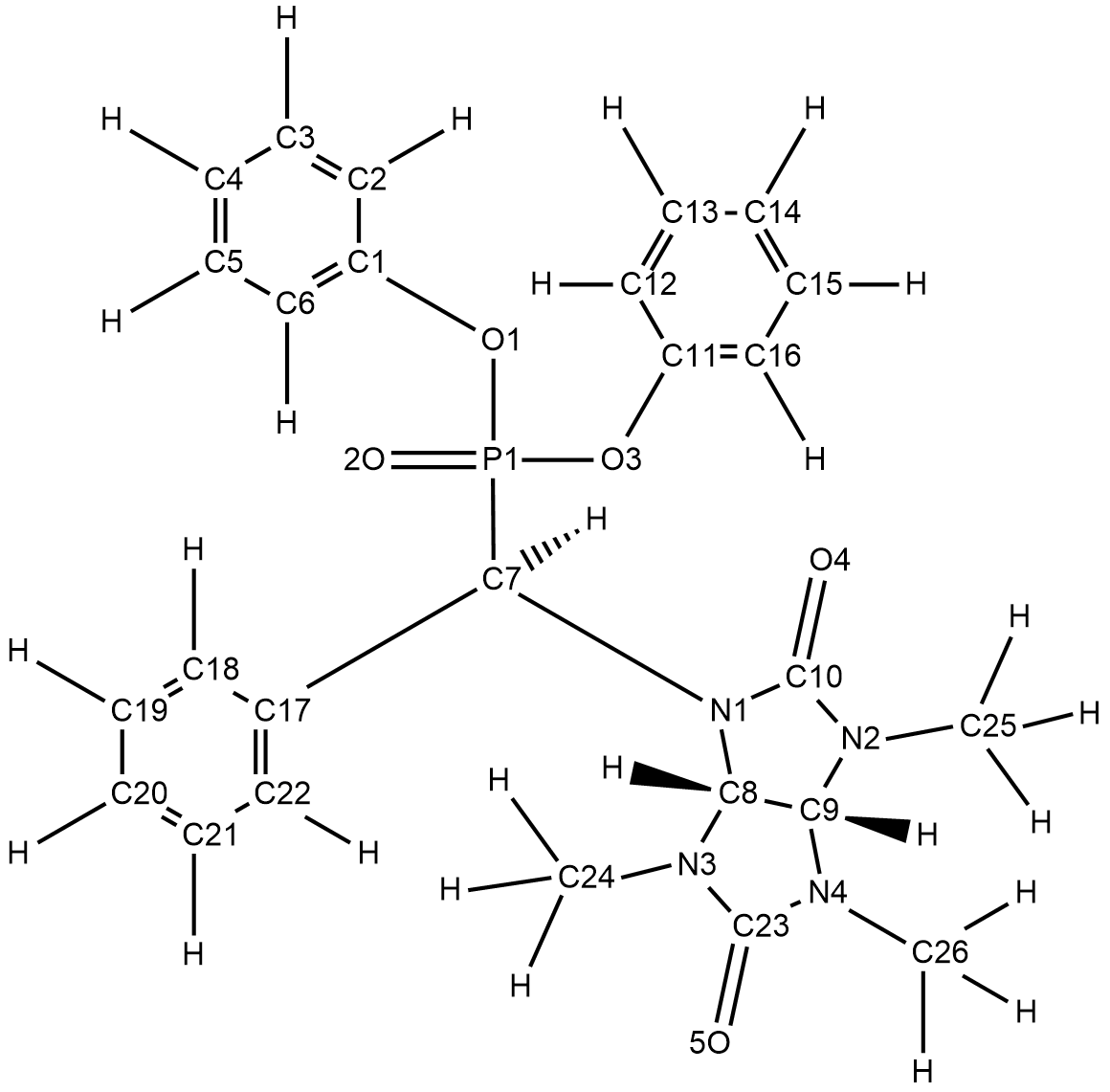 | | | |
| Bond length, A | | | |
| C7-P1 | 1.81 | 1.82 | 0.28 |
| C7-C17 | 1.53 | 1.52 | 0.69 |
| C7-N1 | 1.46 | 1.45 | 0.66 |
| C7-H | 1.00 | 1.09 | 9.48 |
| P1-O3 | 1.59 | 1.60 | 1.06 |
| P1-O1 | 1.58 | 1.59 | 0.54 |
| P1=O2 | 1.47 | 1.47 | 0.26 |
| C17-C22 | 1.38 | 1.39 | 0.45 |
| C22-H | 0.95 | 1.08 | 14.21 |
| N1-C10 | 1.38 | 1.38 | 0.05 |
| N1-C8 | 1.47 | 1.45 | 1.32 |
| C8-H | 1.00 | 1.09 | 9.29 |
| C8-N3 | 1.43 | 1.45 | 1.13 |
| C10=O4 | 1.23 | 1.22 | 0.60 |
| N3-C24 | 1.46 | 1.45 | 0.24 |
| Angles, ° | | | |
| C7-P1=O2 | 115.82 | 116.57 | 0.65 |
| C7-P1=O3 | 104.14 | 103.74 | 0.38 |
| C7-P1=O1 | 101.94 | 102.01 | 0.07 |
| C7-N1-C10 | 120.53 | 120.97 | 0.36 |
| C7-N1-C8 | 126.96 | 126.81 | 0.11 |
| C7-C17=C18 | 123.19 | 123.94 | 0.61 |
| C17=C18=C19 | 120.43 | 120.12 | 0.26 |

**Table S6**. Comparison of geometric parameters of **4a″ (**RSS) optimized molecular structure and experimental data.

| Parameter | **4a″ (**RSS)  Experiment data | **4a″ (**RSS) calculation data  m062x/6-311+G(2d,p) | δ, %  Relative error |
| --- | --- | --- | --- |
| 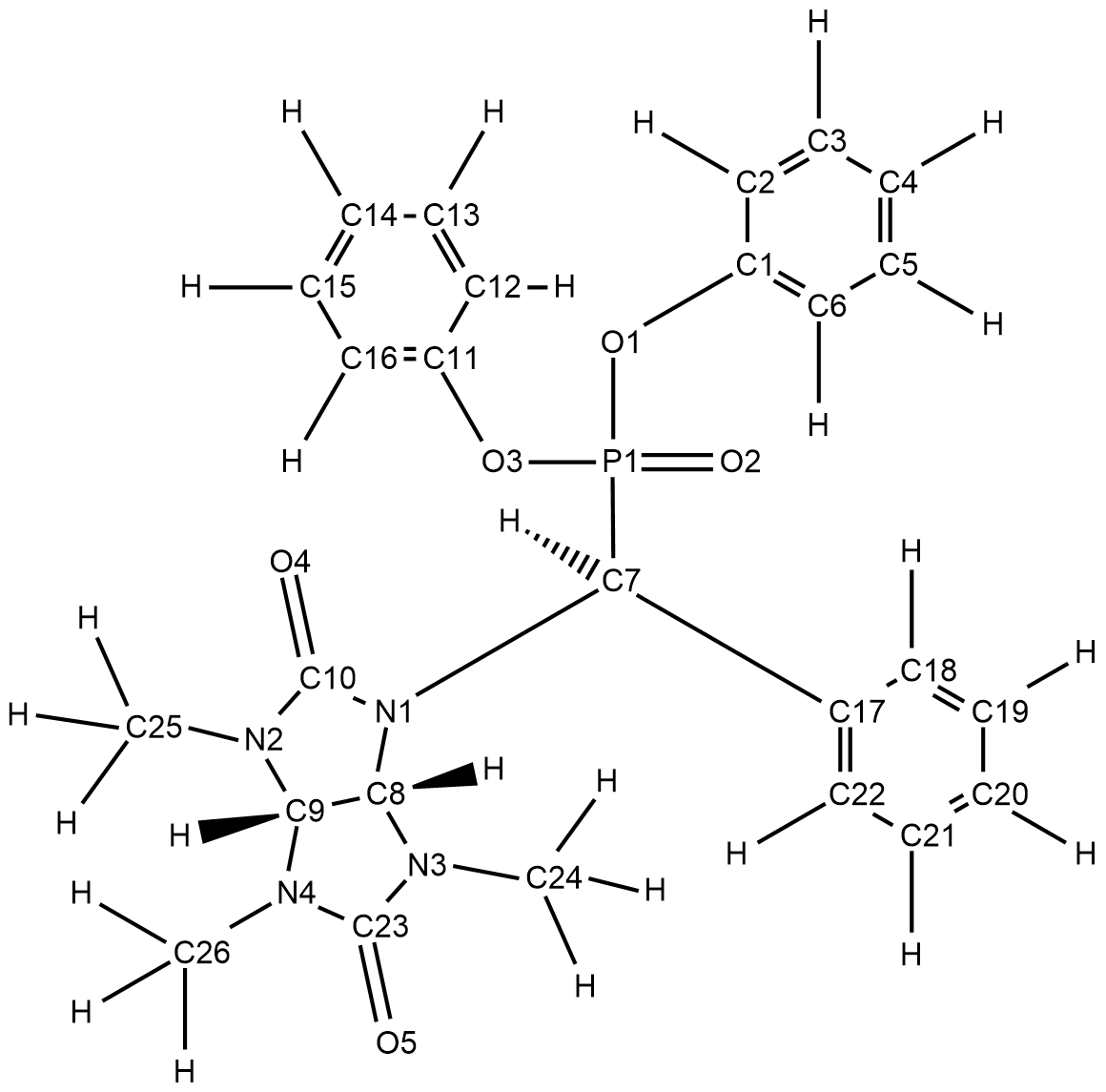 | | | |
| Bond length, A | | | |
| C7-P1 | 1.81 | 1.82 | 0.28 |
| C7-C17 | 1.53 | 1.52 | 0.69 |
| C7-N1 | 1.46 | 1.45 | 0.66 |
| C7-H | 1.00 | 1.09 | 9.48 |
| P1-O3 | 1.59 | 1.60 | 1.06 |
| P1-O1 | 1.58 | 1.59 | 0.54 |
| P1=O2 | 1.47 | 1.47 | 0.26 |
| C17-C22 | 1.38 | 1.39 | 0.45 |
| C22-H | 0.95 | 1.08 | 14.21 |
| N1-C10 | 1.38 | 1.38 | 0.05 |
| N1-C8 | 1.47 | 1.45 | 1.32 |
| C8-H | 1.00 | 1.09 | 9.29 |
| C8-N3 | 1.43 | 1.45 | 1.13 |
| C10=O4 | 1.23 | 1.22 | 0.60 |
| N3-C24 | 1.46 | 1.45 | 0.24 |
| Angles, ° | | | |
| C7-P1=O2 | 115.82 | 116.57 | 0.65 |
| C7-P1=O3 | 104.14 | 103.74 | 0.38 |
| C7-P1=O1 | 101.94 | 102.01 | 0.07 |
| C7-N1-C10 | 120.53 | 120.97 | 0.36 |
| C7-N1-C8 | 126.96 | 126.81 | 0.11 |
| C7-C17=C18 | 123.19 | 123.94 | 0.61 |
| C17=C18=C19 | 120.43 | 120.12 | 0.26 |

**Table S7**. Cartesian coordinates of the all structures of phosphonate glycolurils **4a**-**4f**.

**4a′ (**RSR)

P 1.116219000000 -0.105428000000 0.328243000000

O 1.523214000000 0.280305000000 1.681779000000

O -1.059608000000 -1.237727000000 1.963003000000

O 1.141930000000 -1.663316000000 -0.037579000000

O 2.071262000000 0.541705000000 -0.798294000000

O -5.918520000000 0.770422000000 -0.839923000000

N -4.882565000000 -1.270609000000 -0.558307000000

N -1.591611000000 -0.493220000000 -0.145707000000

N -3.005501000000 -1.872804000000 0.883909000000

N -3.635740000000 0.510722000000 -1.056298000000

C -0.753770000000 1.855637000000 0.075606000000

C -0.282049000000 2.904032000000 -0.707540000000

H 0.257152000000 2.690072000000 -1.624419000000

C -1.813776000000 -1.206426000000 1.008587000000

C -4.916294000000 0.077160000000 -0.809297000000

C -1.226189000000 4.491716000000 0.833172000000

H -1.419182000000 5.516580000000 1.124354000000

C -2.701023000000 -0.595420000000 -1.067586000000

H -2.333826000000 -0.785389000000 -2.079014000000

C -0.501664000000 0.424789000000 -0.359004000000

H -0.298209000000 0.420562000000 -1.435034000000

C -1.691819000000 3.444273000000 1.621228000000

H -2.243127000000 3.653325000000 2.529342000000

C -0.516408000000 4.220268000000 -0.329523000000

H -0.155126000000 5.031167000000 -0.949337000000

C -3.287559000000 -3.040855000000 1.698563000000

H -4.271395000000 -2.968384000000 2.159124000000

H -2.534645000000 -3.099189000000 2.481388000000

H -3.244009000000 -3.946682000000 1.087898000000

C -3.533899000000 -1.754903000000 -0.465343000000

H -3.426202000000 -2.704756000000 -0.995276000000

C -1.451505000000 2.129268000000 1.248043000000

H -1.812917000000 1.314438000000 1.864639000000

C -6.007780000000 -1.968149000000 0.025674000000

H -6.923050000000 -1.589758000000 -0.423848000000

H -6.060792000000 -1.826681000000 1.108065000000

H -5.917385000000 -3.032091000000 -0.193610000000

C -3.406644000000 1.747925000000 -1.782657000000

H -4.314369000000 2.020056000000 -2.319423000000

H -2.598509000000 1.598960000000 -2.501168000000

H -3.129082000000 2.560851000000 -1.110701000000

C 2.385337000000 -2.291342000000 -0.142947000000

C 2.756347000000 -2.780221000000 -1.384201000000

C 3.206832000000 -2.409702000000 0.966250000000

C 3.992528000000 -3.399091000000 -1.518162000000

H 2.084618000000 -2.661796000000 -2.224551000000

C 4.444960000000 -3.023106000000 0.815772000000

H 2.881205000000 -2.013481000000 1.919126000000

C 4.840612000000 -3.514628000000 -0.422226000000

H 4.296433000000 -3.782156000000 -2.484005000000

H 5.100870000000 -3.114541000000 1.672157000000

H 5.807016000000 -3.989335000000 -0.533392000000

C 3.285467000000 1.143495000000 -0.466078000000

C 4.460581000000 0.467946000000 -0.750443000000

C 3.289686000000 2.412891000000 0.087123000000

C 5.673188000000 1.088403000000 -0.473788000000

H 4.417939000000 -0.527154000000 -1.177626000000

C 4.507845000000 3.020687000000 0.361398000000

H 2.348982000000 2.905724000000 0.301007000000

C 5.699857000000 2.361721000000 0.081624000000

H 6.598356000000 0.570417000000 -0.692983000000

H 4.523685000000 4.012528000000 0.795104000000

H 6.646817000000 2.840202000000 0.295986000000

**4a″** (SRR)

P -1.530182000000 -0.013529000000 -0.022051000000

O -1.493604000000 0.101368000000 -1.486103000000

O -1.373870000000 -1.505306000000 0.540418000000

O 1.322767000000 -0.378667000000 2.847585000000

O 4.642143000000 1.686566000000 -1.741782000000

O -2.876334000000 0.432785000000 0.698280000000

N 1.009073000000 0.306626000000 0.665072000000

N 2.793683000000 -0.928331000000 1.156962000000

N 2.473531000000 1.324006000000 -1.029136000000

C -0.267410000000 2.433260000000 0.694780000000

C 1.683446000000 -0.339273000000 1.684685000000

C 1.692326000000 0.177457000000 -0.606810000000

H 0.994916000000 -0.121189000000 -1.393307000000

C 2.773992000000 -0.889703000000 -0.296422000000

H 2.545240000000 -1.884638000000 -0.692602000000

C 3.782678000000 0.964425000000 -1.266625000000

C -4.103327000000 -0.049805000000 0.221592000000

C -0.276989000000 0.929545000000 0.899897000000

H -0.499881000000 0.719550000000 1.951193000000

C -0.453784000000 -2.435959000000 0.060664000000

C 0.645331000000 3.163758000000 1.455965000000

H 1.293205000000 2.642702000000 2.152988000000

C -1.091973000000 3.098734000000 -0.203205000000

H -1.793446000000 2.551095000000 -0.820407000000

C -4.820003000000 0.721920000000 -0.675602000000

H -4.413654000000 1.665270000000 -1.017205000000

C -0.084244000000 5.201383000000 0.408056000000

H -0.009324000000 6.275196000000 0.290920000000

C 0.311459000000 -3.098733000000 1.008881000000

H 0.206760000000 -2.833969000000 2.054041000000

C -0.359215000000 -2.738770000000 -1.290038000000

H -0.977347000000 -2.214037000000 -2.004884000000

C 3.529197000000 -1.940516000000 1.892787000000

H 2.929109000000 -2.271016000000 2.739961000000

H 4.480649000000 -1.555076000000 2.259564000000

H 3.720420000000 -2.791497000000 1.236687000000

C 0.739211000000 4.538388000000 1.314089000000

H 1.454478000000 5.094535000000 1.906854000000

C -4.576432000000 -1.262591000000 0.692011000000

H -3.983318000000 -1.833672000000 1.394190000000

C -0.999500000000 4.480787000000 -0.344437000000

H -1.640480000000 4.989043000000 -1.053661000000

C 1.879175000000 2.420237000000 -1.778573000000

H 0.990790000000 2.062769000000 -2.304808000000

H 2.609001000000 2.777011000000 -2.503093000000

H 1.592188000000 3.244146000000 -1.125508000000

C -5.812434000000 -1.712949000000 0.244844000000

H -6.196895000000 -2.658699000000 0.604985000000

C 1.310861000000 -4.400759000000 -0.756556000000

H 1.998976000000 -5.171889000000 -1.077874000000

C 1.190842000000 -4.089379000000 0.593307000000

H 1.783812000000 -4.617363000000 1.329605000000

C 0.537786000000 -3.722772000000 -1.691013000000

H 0.621131000000 -3.964308000000 -2.743040000000

C -6.550829000000 -0.955434000000 -0.656224000000

H -7.513222000000 -1.310866000000 -1.001111000000

N 3.950333000000 -0.347726000000 -0.911476000000

C 5.253036000000 -0.962471000000 -0.787606000000

H 5.671827000000 -0.835766000000 0.213834000000

H 5.921176000000 -0.501154000000 -1.511184000000

H 5.169828000000 -2.027095000000 -1.008660000000

C -6.054841000000 0.260010000000 -1.113423000000

H -6.628526000000 0.853127000000 -1.813992000000

**4a′ (**SRS)

P -1.116209000000 -0.105330000000 0.328442000000

O -1.523104000000 0.280686000000 1.681937000000

O 1.059752000000 -1.237499000000 1.963222000000

O -1.141939000000 -1.663277000000 -0.037068000000

O -2.071362000000 0.541513000000 -0.798144000000

O 5.917989000000 0.770592000000 -0.841230000000

N 4.882414000000 -1.270453000000 -0.558397000000

N 1.591408000000 -0.493607000000 -0.145779000000

N 3.005630000000 -1.872653000000 0.884134000000

N 3.635220000000 0.510402000000 -1.057104000000

C -2.385427000000 -2.291236000000 -0.142592000000

C 0.754165000000 1.855486000000 0.076043000000

C 0.282007000000 2.904068000000 -0.706599000000

H -0.257660000000 2.690303000000 -1.623252000000

C 1.813836000000 -1.206390000000 1.008730000000

C 4.915877000000 0.077178000000 -0.810084000000

C 1.226916000000 4.491399000000 0.833997000000

H 1.419999000000 5.516195000000 1.125354000000

C 2.700693000000 -0.595914000000 -1.067781000000

H 2.333368000000 -0.786320000000 -2.079080000000

C 0.501722000000 0.424771000000 -0.358817000000

H 0.298233000000 0.420804000000 -1.434846000000

C 0.516502000000 4.220215000000 -0.328375000000

H 0.154832000000 5.031253000000 -0.947784000000

C 3.287655000000 -3.040706000000 1.698837000000

H 4.271676000000 -2.968413000000 2.159029000000

H 2.535009000000 -3.098703000000 2.481942000000

H 3.243638000000 -3.946600000000 1.088317000000

C 3.533886000000 -1.755020000000 -0.465235000000

H 3.426318000000 -2.705048000000 -0.994884000000

C 1.452584000000 2.128840000000 1.248128000000

H 1.814375000000 1.313920000000 1.864370000000

C -2.756222000000 -2.780174000000 -1.383871000000

H -2.084352000000 -2.661856000000 -2.224129000000

C 6.007827000000 -1.967592000000 0.025668000000

H 6.922980000000 -1.589002000000 -0.423923000000

H 6.060789000000 -1.825959000000 1.108039000000

H 5.917740000000 -3.031590000000 -0.193472000000

C -3.992395000000 -3.399043000000 -1.517974000000

H -4.296161000000 -3.782154000000 -2.483840000000

C 3.405761000000 1.747309000000 -1.783868000000

H 4.313299000000 2.019323000000 -2.321002000000

H 2.597430000000 1.597947000000 -2.502084000000

H 3.128327000000 2.560457000000 -1.112143000000

C -3.207073000000 -2.409496000000 0.966493000000

H -2.881574000000 -2.013210000000 1.919378000000

C -4.840646000000 -3.514496000000 -0.422168000000

H -5.807042000000 -3.989205000000 -0.533436000000

C -4.445190000000 -3.022897000000 0.815866000000

H -5.101243000000 -3.114289000000 1.672149000000

C -3.285506000000 1.143466000000 -0.466188000000

C -4.460638000000 0.467941000000 -0.750588000000

C -3.289755000000 2.412992000000 0.086727000000

C -5.673257000000 1.088509000000 -0.474258000000

H -4.417991000000 -0.527244000000 -1.177577000000

C -4.507933000000 3.020906000000 0.360680000000

H -2.349077000000 2.905846000000 0.300645000000

C -5.699942000000 2.361951000000 0.080879000000

H -6.598417000000 0.570507000000 -0.693440000000

H -4.523792000000 4.012853000000 0.794141000000

H -6.646903000000 2.840549000000 0.294972000000

C 1.693061000000 3.443771000000 1.621493000000

H 2.244898000000 3.652617000000 2.529338000000

**4a″ (**RSS)

P 1.530170000000 -0.013468000000 -0.022122000000

O 1.493621000000 0.101478000000 -1.486167000000

O 1.373826000000 -1.505241000000 0.540364000000

O -1.322710000000 -0.378695000000 2.847583000000

O -4.642003000000 1.686582000000 -1.741893000000

O 2.876317000000 0.432860000000 0.698198000000

N -1.009057000000 0.306562000000 0.665062000000

N -2.793726000000 -0.928305000000 1.157015000000

N -2.473405000000 1.323913000000 -1.029267000000

C 0.267331000000 2.433265000000 0.694802000000

C -1.683415000000 -0.339303000000 1.684693000000

C -1.692296000000 0.177342000000 -0.606817000000

H -0.994894000000 -0.121403000000 -1.393285000000

C -2.774025000000 -0.889732000000 -0.296364000000

H -2.545333000000 -1.884692000000 -0.692516000000

C -3.782581000000 0.964414000000 -1.266706000000

C 4.103325000000 -0.049804000000 0.221600000000

C 0.276980000000 0.929542000000 0.899862000000

H 0.499926000000 0.719541000000 1.951147000000

C 0.453754000000 -2.435906000000 0.060575000000

C -0.645602000000 3.163686000000 1.455828000000

H -1.293612000000 2.642569000000 2.152678000000

C 1.092056000000 3.098818000000 -0.202978000000

H 1.793689000000 2.551250000000 -0.820059000000

C 4.820233000000 0.722096000000 -0.675264000000

H 4.414039000000 1.665595000000 -1.016641000000

C 0.084110000000 5.201394000000 0.408174000000

H 0.009166000000 6.275209000000 0.291070000000

C -0.311287000000 -3.098844000000 1.008847000000

H -0.206453000000 -2.834153000000 2.054011000000

C 0.359013000000 -2.738582000000 -1.290137000000

H 0.976983000000 -2.213733000000 -2.005034000000

C -3.529110000000 -1.940610000000 1.892825000000

H -2.929097000000 -2.270815000000 2.740163000000

H -4.480745000000 -1.555381000000 2.259340000000

H -3.719950000000 -2.791751000000 1.236815000000

C -0.739507000000 4.538320000000 1.313998000000

H -1.454923000000 5.094406000000 1.906641000000

C 4.576195000000 -1.262784000000 0.691740000000

H 3.982911000000 -1.834009000000 1.393656000000

C 0.999553000000 4.480872000000 -0.344165000000

H 1.640658000000 4.989190000000 -1.053232000000

C -1.878957000000 2.419993000000 -1.778862000000

H -0.990549000000 2.062387000000 -2.304965000000

H -2.608718000000 2.776658000000 -2.503500000000

H -1.591993000000 3.244008000000 -1.125925000000

C 5.812213000000 -1.713162000000 0.244616000000

H 6.196502000000 -2.659066000000 0.604535000000

C -1.310834000000 -4.400785000000 -0.756561000000

H -1.998920000000 -5.171951000000 -1.077854000000

C -1.190633000000 -4.089534000000 0.593320000000

H -1.783443000000 -4.617644000000 1.329656000000

C -0.537969000000 -3.722631000000 -1.691065000000

H -0.621463000000 -3.964058000000 -2.743105000000

C 6.550840000000 -0.955477000000 -0.656114000000

H 7.513241000000 -1.310929000000 -1.000959000000

C -5.253142000000 -0.962207000000 -0.787287000000

H -5.170094000000 -2.026925000000 -1.007933000000

H -5.671868000000 -0.835051000000 0.214124000000

H -5.921245000000 -0.501082000000 -1.511021000000

N -3.950341000000 -0.347707000000 -0.911451000000

C 6.055078000000 0.260169000000 -1.113033000000

H 6.628951000000 0.853419000000 -1.813335000000

**4c′ (**RSR_OH_m)

P 1.111783000000 -0.254288000000 0.357387000000

O 1.536138000000 0.096589000000 1.715173000000

O -1.089809000000 -1.391046000000 1.980808000000

O 1.107729000000 -1.804744000000 -0.038720000000

O 2.068053000000 0.400425000000 -0.763457000000

O -5.906964000000 0.642811000000 -0.878612000000

N -4.874364000000 -1.399871000000 -0.596230000000

N -1.594103000000 -0.621590000000 -0.124976000000

N -3.017067000000 -2.020305000000 0.864866000000

N -3.621354000000 0.383787000000 -1.066894000000

C -0.760604000000 1.719790000000 0.193340000000

C -0.221144000000 2.783489000000 -0.517639000000

C -1.830349000000 -1.351045000000 1.016072000000

C -4.905009000000 -0.050466000000 -0.839638000000

C -1.187805000000 4.328360000000 1.059930000000

H -1.357849000000 5.346081000000 1.391898000000

C -2.685582000000 -0.720367000000 -1.068153000000

H -2.299043000000 -0.902310000000 -2.073849000000

C -0.508892000000 0.308066000000 -0.302270000000

H -0.308545000000 0.348124000000 -1.378037000000

C -1.722235000000 3.256450000000 1.762724000000

H -2.310643000000 3.447635000000 2.651282000000

C -0.429792000000 4.087011000000 -0.080394000000

C -3.309585000000 -3.196886000000 1.663373000000

H -4.296910000000 -3.126927000000 2.116892000000

H -2.563203000000 -3.266683000000 2.451522000000

H -3.263705000000 -4.095530000000 1.042464000000

C -3.527191000000 -1.886177000000 -0.489909000000

H -3.412977000000 -2.830023000000 -1.029060000000

C -1.508893000000 1.951036000000 1.343113000000

H -1.925450000000 1.122454000000 1.901267000000

C -6.006869000000 -2.101164000000 -0.031301000000

H -6.916538000000 -1.717731000000 -0.487824000000

H -6.071914000000 -1.969136000000 1.051670000000

H -5.915271000000 -3.163290000000 -0.258819000000

C -3.375112000000 1.628650000000 -1.772849000000

H -4.285495000000 1.929354000000 -2.289291000000

H -2.582371000000 1.476963000000 -2.508169000000

H -3.071615000000 2.422694000000 -1.089853000000

C 2.338234000000 -2.453391000000 -0.167514000000

C 2.689415000000 -2.923236000000 -1.421769000000

C 3.166235000000 -2.610136000000 0.931908000000

C 3.912394000000 -3.562287000000 -1.579231000000

H 2.013095000000 -2.774179000000 -2.253507000000

C 4.391046000000 -3.243695000000 0.758170000000

H 2.855476000000 -2.227740000000 1.895297000000

C 4.767090000000 -3.716514000000 -0.493195000000

H 4.200893000000 -3.930814000000 -2.555370000000

H 5.052265000000 -3.365924000000 1.606547000000

H 5.723419000000 -4.206765000000 -0.622217000000

C 3.296703000000 0.973317000000 -0.430768000000

C 4.456503000000 0.276103000000 -0.725753000000

C 3.330110000000 2.237572000000 0.133226000000

C 5.682871000000 0.868642000000 -0.448756000000

H 4.391686000000 -0.713883000000 -1.161831000000

C 4.561729000000 2.817604000000 0.407280000000

H 2.401580000000 2.748401000000 0.356844000000

C 5.738543000000 2.136498000000 0.117050000000

H 6.596004000000 0.333330000000 -0.676638000000

H 4.600118000000 3.805163000000 0.849306000000

H 6.696202000000 2.593470000000 0.330929000000

H 0.352546000000 2.616628000000 -1.422849000000

O 0.127446000000 5.085506000000 -0.816949000000

H -0.081892000000 5.941126000000 -0.425003000000

**4e′** (RSR_OH_p)

P 1.160510000000 -0.198240000000 0.318355000000

O 1.556983000000 0.264046000000 1.651043000000

O -0.982115000000 -1.302121000000 2.012152000000

O 1.236913000000 -1.771688000000 0.031143000000

O 2.094814000000 0.422922000000 -0.840558000000

O -5.906019000000 0.261787000000 -0.974093000000

N -4.759853000000 -1.693380000000 -0.552157000000

N -1.528468000000 -0.703416000000 -0.137390000000

N -2.879939000000 -2.099854000000 0.953571000000

N -3.607436000000 0.123438000000 -1.141224000000

C -0.778025000000 1.681266000000 -0.030833000000

C -0.351029000000 2.712973000000 -0.858111000000

C -1.725768000000 -1.363532000000 1.050868000000

C -4.865828000000 -0.367807000000 -0.887294000000

C -1.337712000000 4.333015000000 0.612968000000

C -2.610848000000 -0.925026000000 -1.068422000000

H -2.213593000000 -1.153735000000 -2.060514000000

C -0.472297000000 0.243415000000 -0.394677000000

H -0.265593000000 0.192855000000 -1.468648000000

C -1.767620000000 3.306933000000 1.451104000000

H -2.321469000000 3.556575000000 2.347089000000

C -0.624780000000 4.036948000000 -0.543508000000

C -3.086861000000 -3.247994000000 1.817992000000

H -4.113617000000 -3.281947000000 2.177037000000

H -2.417189000000 -3.154640000000 2.669691000000

H -2.863001000000 -4.175318000000 1.283256000000

C -3.388003000000 -2.093146000000 -0.409308000000

H -3.216560000000 -3.065020000000 -0.879509000000

C -1.481219000000 1.991472000000 1.130835000000

H -1.813859000000 1.197305000000 1.789308000000

C -5.854770000000 -2.414997000000 0.059621000000

H -6.781568000000 -2.114717000000 -0.424050000000

H -5.932169000000 -2.210934000000 1.130609000000

H -5.703998000000 -3.483746000000 -0.093145000000

C -3.429215000000 1.329619000000 -1.929599000000

H -4.371347000000 1.569676000000 -2.419846000000

H -2.665341000000 1.157763000000 -2.691095000000

H -3.122946000000 2.172831000000 -1.309546000000

C 2.498048000000 -2.365871000000 -0.049611000000

C 2.877247000000 -2.908121000000 -1.265964000000

C 3.329173000000 -2.401673000000 1.058622000000

C 4.130337000000 -3.496998000000 -1.375740000000

H 2.198430000000 -2.854691000000 -2.107319000000

C 4.583988000000 -2.985807000000 0.931969000000

H 2.997128000000 -1.966075000000 1.991998000000

C 4.987456000000 -3.530101000000 -0.281141000000

H 4.439975000000 -3.921779000000 -2.322194000000

H 5.246264000000 -3.013033000000 1.787892000000

H 5.966481000000 -3.982325000000 -0.374049000000

C 3.294044000000 1.069314000000 -0.541844000000

C 4.484640000000 0.415950000000 -0.813332000000

C 3.268414000000 2.357822000000 -0.034957000000

C 5.682261000000 1.078370000000 -0.570791000000

H 4.465636000000 -0.594978000000 -1.203481000000

C 4.471991000000 3.007520000000 0.206092000000

H 2.316023000000 2.832700000000 0.168741000000

C 5.679028000000 2.370948000000 -0.061149000000

H 6.619366000000 0.577747000000 -0.779525000000

H 4.464311000000 4.014464000000 0.603621000000

H 6.614544000000 2.881551000000 0.127765000000

H 0.192254000000 2.484930000000 -1.768998000000

H -0.296358000000 4.835440000000 -1.198880000000

O -1.647836000000 5.606124000000 0.975028000000

H -1.297431000000 6.229684000000 0.328395000000

**4c″** (SRR_OH_m)

P -1.589217000000 -0.004407000000 -0.064341000000

O -1.545534000000 0.007381000000 -1.532519000000

O -1.609863000000 -1.464075000000 0.595574000000

O 1.262060000000 -0.498835000000 2.803331000000

O 4.659799000000 0.962676000000 -1.978885000000

O -2.867244000000 0.646493000000 0.623224000000

N 0.977852000000 0.068310000000 0.583520000000

N 2.650059000000 -1.290447000000 1.138227000000

N 2.485652000000 0.838435000000 -1.202369000000

C -0.055811000000 2.324185000000 0.497739000000

C 1.606623000000 -0.568869000000 1.637003000000

C 1.623192000000 -0.206124000000 -0.685372000000

H 0.887075000000 -0.500916000000 -1.437099000000

C 2.618370000000 -1.336429000000 -0.313740000000

H 2.305935000000 -2.331682000000 -0.646409000000

C 3.756999000000 0.354634000000 -1.429467000000

C -4.147637000000 0.256439000000 0.205767000000

C -0.226875000000 0.843978000000 0.793034000000

H -0.456962000000 0.721807000000 1.856707000000

C -0.799017000000 -2.518932000000 0.178799000000

C 0.963651000000 2.978386000000 1.182952000000

C -0.841714000000 3.023945000000 -0.409889000000

H -1.625861000000 2.529825000000 -0.967859000000

C -4.740474000000 0.916518000000 -0.856077000000

H -4.201672000000 1.702751000000 -1.368999000000

C 0.424740000000 5.030544000000 0.040387000000

H 0.619361000000 6.081380000000 -0.140294000000

C -0.087451000000 -3.184319000000 1.166076000000

H -0.144812000000 -2.832046000000 2.188797000000

C -0.759711000000 -2.928111000000 -1.146183000000

H -1.334317000000 -2.397410000000 -1.892419000000

C 3.302616000000 -2.320717000000 1.923623000000

H 2.732159000000 -2.481185000000 2.837462000000

H 4.322173000000 -2.037818000000 2.185509000000

H 3.327425000000 -3.249055000000 1.349284000000

C 1.204184000000 4.325169000000 0.955119000000

C -4.792177000000 -0.754630000000 0.896795000000

H -4.290783000000 -1.243526000000 1.721903000000

C -0.594093000000 4.374962000000 -0.630955000000

H -1.197704000000 4.921347000000 -1.344470000000

C 1.966929000000 1.915227000000 -2.032281000000

H 1.015135000000 1.607045000000 -2.470716000000

H 2.683803000000 2.116042000000 -2.826506000000

H 1.807676000000 2.825904000000 -1.454672000000

C -6.076537000000 -1.113038000000 0.507235000000

H -6.594604000000 -1.901311000000 1.038419000000

C 0.747818000000 -4.705045000000 -0.507446000000

H 1.351131000000 -5.562218000000 -0.777307000000

C 0.682445000000 -4.285648000000 0.816835000000

H 1.232403000000 -4.814970000000 1.584942000000

C 0.028982000000 -4.023451000000 -1.481870000000

H 0.069165000000 -4.348831000000 -2.513688000000

C -6.692669000000 -0.465519000000 -0.557076000000

H -7.693377000000 -0.748761000000 -0.856598000000

N 3.827569000000 -0.930982000000 -0.966879000000

C 5.070937000000 -1.658152000000 -0.848881000000

H 5.530919000000 -1.525338000000 0.133172000000

H 5.754890000000 -1.292683000000 -1.611415000000

H 4.883485000000 -2.719361000000 -1.016059000000

C -6.024714000000 0.547258000000 -1.235683000000

H -6.502711000000 1.054183000000 -2.064174000000

H 1.588337000000 2.441756000000 1.888694000000

O 2.217026000000 4.907098000000 1.651874000000

H 2.300454000000 5.834493000000 1.401967000000

**4e″** (SRR_OH_p)

P -1.531132000000 -0.203182000000 -0.022468000000

O -1.496540000000 -0.099995000000 -1.487603000000

O -1.377865000000 -1.691404000000 0.551192000000

O 1.324096000000 -0.552350000000 2.847234000000

O 4.640241000000 1.467188000000 -1.765461000000

O -2.876656000000 0.250792000000 0.694809000000

N 1.008633000000 0.113296000000 0.658719000000

N 2.792562000000 -1.119047000000 1.160288000000

N 2.472149000000 1.113277000000 -1.047056000000

C -0.264215000000 2.244164000000 0.664431000000

C 1.682902000000 -0.523686000000 1.683249000000

C 1.690475000000 -0.028422000000 -0.612358000000

H 0.991976000000 -0.333887000000 -1.395243000000

C 2.771473000000 -1.093620000000 -0.293181000000

H 2.541400000000 -2.091789000000 -0.680309000000

C 3.780655000000 0.750415000000 -1.282189000000

C -4.104234000000 -0.232399000000 0.220543000000

C -0.275139000000 0.745067000000 0.889282000000

H -0.497222000000 0.542782000000 1.942140000000

C -0.460325000000 -2.627800000000 0.078110000000

C 0.644408000000 2.989355000000 1.413997000000

C -1.081959000000 2.906896000000 -0.243318000000

H -1.784080000000 2.357640000000 -0.858551000000

C -4.819721000000 0.536089000000 -0.680393000000

H -4.412015000000 1.477316000000 -1.026222000000

C -0.078076000000 5.010909000000 0.342564000000

C 0.303618000000 -3.285778000000 1.030814000000

H 0.199930000000 -3.013342000000 2.074093000000

C -0.367004000000 -2.940888000000 -1.270362000000

H -0.984142000000 -2.419939000000 -1.988832000000

C 3.525121000000 -2.127359000000 1.904303000000

H 2.923491000000 -2.450647000000 2.753158000000

H 4.476984000000 -1.741333000000 2.269454000000

H 3.715502000000 -2.983418000000 1.254582000000

C 0.745528000000 4.360421000000 1.259375000000

C -4.579262000000 -1.442349000000 0.696424000000

H -3.987161000000 -2.011023000000 1.401406000000

C -0.993782000000 4.282921000000 -0.405675000000

H -1.623758000000 4.799906000000 -1.118058000000

C 1.877303000000 2.201742000000 -1.807221000000

H 0.983758000000 1.840633000000 -2.322014000000

H 2.603347000000 2.546266000000 -2.541477000000

H 1.597361000000 3.035219000000 -1.163390000000

C -5.815765000000 -1.893058000000 0.250972000000

H -6.201634000000 -2.836640000000 0.615301000000

C 1.299096000000 -4.603170000000 -0.725457000000

H 1.985027000000 -5.378477000000 -1.041324000000

C 1.180379000000 -4.281588000000 0.622126000000

H 1.772268000000 -4.805671000000 1.362088000000

C 0.527311000000 -3.930065000000 -1.664504000000

H 0.609466000000 -4.179609000000 -2.714760000000

C -6.552869000000 -1.138735000000 -0.653819000000

H -7.515609000000 -1.494478000000 -0.997412000000

N 3.947816000000 -0.558273000000 -0.914251000000

C 5.250204000000 -1.172234000000 -0.783936000000

H 5.667102000000 -1.038758000000 0.217434000000

H 5.919640000000 -0.715783000000 -1.509392000000

H 5.167438000000 -2.238367000000 -0.997856000000

C -6.055060000000 0.073909000000 -1.116472000000

H -6.627659000000 0.664550000000 -1.820014000000

H 1.293878000000 2.484219000000 2.121097000000

H 1.459832000000 4.928074000000 1.844542000000

O -0.025124000000 6.354178000000 0.140607000000

H 0.652152000000 6.747819000000 0.702634000000

**4c′** (SRS_OH_m)

P -1.111783000000 -0.254280000000 0.357399000000

O -1.536127000000 0.096610000000 1.715186000000

O 1.089750000000 -1.391160000000 1.980734000000

O -1.107796000000 -1.804727000000 -0.038723000000

O -2.068047000000 0.400455000000 -0.763439000000

O 5.906900000000 0.642859000000 -0.878579000000

N 4.874362000000 -1.399850000000 -0.596184000000

N 1.594072000000 -0.621688000000 -0.125035000000

N 3.017046000000 -2.020374000000 0.864833000000

N 3.621307000000 0.383752000000 -1.066945000000

C -2.338319000000 -2.453334000000 -0.167502000000

C 0.760676000000 1.719726000000 0.193336000000

C 0.221209000000 2.783449000000 -0.517591000000

C 1.830312000000 -1.351142000000 1.016016000000

C 4.904965000000 -0.050449000000 -0.839612000000

C 1.187978000000 4.328269000000 1.059954000000

H 1.358060000000 5.345978000000 1.391941000000

C 2.685571000000 -0.720432000000 -1.068195000000

H 2.299051000000 -0.902401000000 -2.073894000000

C 0.508897000000 0.308018000000 -0.302289000000

H 0.308523000000 0.348091000000 -1.378050000000

C 0.429905000000 4.086963000000 -0.080337000000

C 3.309582000000 -3.196950000000 1.663340000000

H 4.296917000000 -3.126986000000 2.116836000000

H 2.563217000000 -3.266740000000 2.451506000000

H 3.263688000000 -4.095599000000 1.042439000000

C 3.527209000000 -1.886213000000 -0.489923000000

H 3.413053000000 -2.830059000000 -1.029084000000

C 1.509024000000 1.950934000000 1.343083000000

C -2.689511000000 -2.923198000000 -1.421747000000

H -2.013180000000 -2.774186000000 -2.253484000000

C 6.006880000000 -2.101102000000 -0.031232000000

H 6.916546000000 -1.717653000000 -0.487749000000

H 6.071908000000 -1.969059000000 1.051738000000

H 5.915313000000 -3.163234000000 -0.258737000000

C -3.912512000000 -3.562209000000 -1.579201000000

H -4.201017000000 -3.930751000000 -2.555332000000

C 3.375076000000 1.628573000000 -1.772984000000

H 4.285448000000 1.929211000000 -2.289484000000

H 2.582315000000 1.476845000000 -2.508273000000

H 3.071623000000 2.422681000000 -1.090045000000

C -3.166336000000 -2.610018000000 0.931917000000

H -2.855573000000 -2.227609000000 1.895300000000

C -4.767220000000 -3.716379000000 -0.493166000000

H -5.723564000000 -4.206602000000 -0.622181000000

C -4.391167000000 -3.243540000000 0.758187000000

H -5.052396000000 -3.365723000000 1.606564000000

C -3.296682000000 0.973371000000 -0.430757000000

C -4.456487000000 0.276191000000 -0.725801000000

C -3.330079000000 2.237602000000 0.133292000000

C -5.682853000000 0.868740000000 -0.448814000000

H -4.391671000000 -0.713782000000 -1.161913000000

C -4.561695000000 2.817645000000 0.407335000000

H -2.401545000000 2.748404000000 0.356956000000

C -5.738514000000 2.136574000000 0.117043000000

H -6.595988000000 0.333452000000 -0.676738000000

H -4.600079000000 3.805186000000 0.849402000000

H -6.696170000000 2.593556000000 0.330917000000

C 1.722414000000 3.256333000000 1.762707000000

H 2.310867000000 3.447493000000 2.651242000000

H 1.925590000000 1.122337000000 1.901206000000

H -0.352529000000 2.616622000000 -1.422777000000

O -0.127330000000 5.085490000000 -0.816858000000

H 0.082074000000 5.941090000000 -0.424908000000

**4e′** (SRS_OH_p)

P -1.160589000000 -0.198546000000 0.318263000000

O -1.557106000000 0.263541000000 1.651005000000

O 0.982022000000 -1.302612000000 2.011995000000

O -1.236973000000 -1.771950000000 0.030747000000

O -2.094948000000 0.422688000000 -0.840553000000

O 5.906015000000 0.262151000000 -0.973695000000

N 4.759963000000 -1.693162000000 -0.552154000000

N 1.528441000000 -0.703543000000 -0.137432000000

N 2.880024000000 -2.099954000000 0.953441000000

N 3.607440000000 0.123689000000 -1.140934000000

C -2.498178000000 -2.366041000000 -0.049907000000

C 0.777862000000 1.681087000000 -0.030801000000

C 0.350991000000 2.712793000000 -0.858172000000

C 1.725751000000 -1.363782000000 1.050758000000

C 4.865865000000 -0.367530000000 -0.887054000000

C 1.337626000000 4.332829000000 0.612948000000

C 2.610942000000 -0.924878000000 -1.068405000000

H 2.213787000000 -1.153449000000 -2.060568000000

C 0.472206000000 0.243230000000 -0.394682000000

H 0.265520000000 0.192719000000 -1.468660000000

C 0.624791000000 4.036751000000 -0.543606000000

C 3.086966000000 -3.248236000000 1.817676000000

H 4.113682000000 -3.282141000000 2.176841000000

H 2.417182000000 -3.155114000000 2.669312000000

H 2.863273000000 -4.175483000000 1.282736000000

C 3.388140000000 -2.093036000000 -0.409425000000

H 3.216770000000 -3.064859000000 -0.879759000000

C 1.480897000000 1.991300000000 1.130934000000

C -2.877717000000 -2.907817000000 -1.266364000000

H -2.199104000000 -2.854121000000 -2.107866000000

C 5.854917000000 -2.414842000000 0.059482000000

H 6.781693000000 -2.114493000000 -0.424188000000

H 5.932358000000 -2.210923000000 1.130494000000

H 5.704148000000 -3.483572000000 -0.093416000000

C -4.130872000000 -3.496577000000 -1.376031000000

H -4.440793000000 -3.920983000000 -2.322560000000

C 3.429234000000 1.329941000000 -1.929211000000

H 4.371379000000 1.570046000000 -2.419411000000

H 2.665387000000 1.158145000000 -2.690748000000

H 3.122946000000 2.173097000000 -1.309094000000

C -3.329017000000 -2.402179000000 1.058525000000

H -2.996700000000 -1.966936000000 1.991969000000

C -4.987709000000 -3.530028000000 -0.281220000000

H -5.966783000000 -3.982163000000 -0.374041000000

C -4.583902000000 -2.986194000000 0.931984000000

H -5.245966000000 -3.013687000000 1.788064000000

C -3.293928000000 1.069475000000 -0.541698000000

C -4.484723000000 0.416328000000 -0.812843000000

C -3.267895000000 2.358055000000 -0.035027000000

C -5.682151000000 1.079057000000 -0.570203000000

H -4.465999000000 -0.594679000000 -1.202808000000

C -4.471288000000 3.008061000000 0.206129000000

H -2.315361000000 2.832760000000 0.168419000000

C -5.678520000000 2.371725000000 -0.060789000000

H -6.619412000000 0.578613000000 -0.778664000000

H -4.463302000000 4.015069000000 0.603491000000

H -6.613878000000 2.882584000000 0.128218000000

C 1.767331000000 3.306768000000 1.451203000000

H 2.321081000000 3.556380000000 2.347253000000

H 1.813382000000 1.197145000000 1.789496000000

H -0.192211000000 2.484731000000 -1.769096000000

H 0.296462000000 4.835234000000 -1.199027000000

O 1.647842000000 5.605932000000 0.974976000000

H 1.297699000000 6.229489000000 0.328206000000

**4c″** (RSS_OH_m)

P 1.589251000000 -0.004578000000 -0.064325000000

O 1.545704000000 0.007296000000 -1.532505000000

O 1.609738000000 -1.464292000000 0.595491000000

O -1.262027000000 -0.498701000000 2.803399000000

O -4.659758000000 0.962961000000 -1.978982000000

O 2.867290000000 0.646145000000 0.623388000000

N -0.977898000000 0.068416000000 0.583574000000

N -2.650211000000 -1.290190000000 1.138372000000

N -2.485660000000 0.838609000000 -1.202336000000

C 0.056082000000 2.324129000000 0.497740000000

C -1.606644000000 -0.568742000000 1.637095000000

C -1.623290000000 -0.206009000000 -0.685290000000

H -0.887220000000 -0.500913000000 -1.437020000000

C -2.618576000000 -1.336199000000 -0.313581000000

H -2.306264000000 -2.331496000000 -0.646242000000

C -3.757046000000 0.354906000000 -1.429426000000

C 4.147673000000 0.256148000000 0.205836000000

C 0.226949000000 0.843893000000 0.793037000000

H 0.457051000000 0.721697000000 1.856706000000

C 0.798765000000 -2.519032000000 0.178685000000

C -0.963268000000 2.978456000000 1.182971000000

C 0.842002000000 3.023773000000 -0.409973000000

H 1.626048000000 2.529554000000 -0.967996000000

C 4.740518000000 0.916345000000 -0.855929000000

H 4.201740000000 1.702656000000 -1.368756000000

C -0.424256000000 5.030504000000 0.040279000000

H -0.618787000000 6.081350000000 -0.140453000000

C 0.087290000000 -3.184496000000 1.165980000000

H 0.144848000000 -2.832364000000 2.188736000000

C 0.759275000000 -2.928077000000 -1.146335000000

H 1.333834000000 -2.397348000000 -1.892588000000

C -3.302878000000 -2.320387000000 1.923767000000

H -2.732873000000 -2.480293000000 2.837985000000

H -4.322680000000 -2.037723000000 2.184959000000

H -3.327073000000 -3.248955000000 1.349774000000

C -1.203707000000 4.325255000000 0.955092000000

C 4.792185000000 -0.755039000000 0.896720000000

H 4.290764000000 -1.244026000000 1.721759000000

C 0.594478000000 4.374793000000 -0.631096000000

H 1.198092000000 4.921094000000 -1.344674000000

C -1.966817000000 1.915334000000 -2.032245000000

H -1.015164000000 1.606969000000 -2.470867000000

H -2.683785000000 2.116373000000 -2.826330000000

H -1.807269000000 2.825934000000 -1.454595000000

C 6.076527000000 -1.113442000000 0.507104000000

H 6.594572000000 -1.901804000000 1.038176000000

C -0.748288000000 -4.704985000000 -0.507601000000

H -1.351677000000 -5.562096000000 -0.777488000000

C -0.682710000000 -4.285742000000 0.816718000000

H -1.232589000000 -4.815122000000 1.584844000000

C -0.029525000000 -4.023333000000 -1.482038000000

H -0.069845000000 -4.348613000000 -2.513883000000

C 6.692669000000 -0.465802000000 -0.557128000000

H 7.693364000000 -0.749042000000 -0.856697000000

C -5.071144000000 -1.657779000000 -0.848857000000

H -4.883700000000 -2.718987000000 -1.016049000000

H -5.531231000000 -1.524992000000 0.133149000000

H -5.755017000000 -1.292279000000 -1.611449000000

N -3.827755000000 -0.930634000000 -0.966699000000

C 6.024741000000 0.547086000000 -1.235595000000

H 6.502747000000 1.054096000000 -2.064030000000

H -1.587960000000 2.441926000000 1.888780000000

O -2.216475000000 4.907293000000 1.651863000000

H -2.299799000000 5.834693000000 1.401941000000

**4e″** (RSS_OH_p)

P 1.530421000000 -0.206802000000 -0.022060000000

O 1.496152000000 -0.104536000000 -1.487269000000

O 1.374091000000 -1.694324000000 0.552568000000

O -1.325747000000 -0.549344000000 2.846629000000

O -4.641386000000 1.473122000000 -1.764253000000

O 2.876813000000 0.244842000000 0.695078000000

N -1.008919000000 0.116230000000 0.658227000000

N -2.794704000000 -1.113627000000 1.159288000000

N -2.473386000000 1.118835000000 -1.045366000000

C 0.269921000000 2.243773000000 0.664247000000

C -1.684280000000 -0.520023000000 1.682579000000

C -1.691464000000 -0.023607000000 -0.612666000000

H -0.993467000000 -0.328320000000 -1.396321000000

C -2.772704000000 -1.088722000000 -0.294240000000

H -2.542483000000 -2.087003000000 -0.680972000000

C -3.781670000000 0.755898000000 -1.281907000000

C 4.103611000000 -0.239261000000 0.219827000000

C 0.276515000000 0.744704000000 0.889037000000

H 0.497648000000 0.541918000000 1.941989000000

C 0.454971000000 -2.629275000000 0.079682000000

C -0.634007000000 2.991846000000 1.416671000000

C 1.086653000000 2.903789000000 -0.246367000000

H 1.784889000000 2.352112000000 -0.863871000000

C 4.819170000000 0.528718000000 -0.681474000000

H 4.412055000000 1.470333000000 -1.026943000000

C 0.090899000000 5.010914000000 0.342202000000

C -0.310366000000 -3.285447000000 1.032524000000

H -0.206544000000 -3.012657000000 2.075697000000

C 0.361446000000 -2.942865000000 -1.268663000000

H 0.979680000000 -2.423395000000 -1.987256000000

C -3.529433000000 -2.120381000000 1.903244000000

H -2.928956000000 -2.444145000000 2.752732000000

H -4.480952000000 -1.732631000000 2.267468000000

H -3.720687000000 -2.976472000000 1.253823000000

C -0.731672000000 4.363130000000 1.261891000000

C 4.577915000000 -1.449677000000 0.695255000000

H 3.985770000000 -2.017896000000 1.400575000000

C 1.002083000000 4.280054000000 -0.408749000000

H 1.631282000000 4.794975000000 -1.123304000000

C -1.878798000000 2.209080000000 -1.803073000000

H -0.989934000000 1.847697000000 -2.325835000000

H -2.608338000000 2.560315000000 -2.530601000000

H -1.591852000000 3.037834000000 -1.156284000000

C 5.813770000000 -1.901383000000 0.249014000000

H 6.199084000000 -2.845317000000 0.613016000000

C -1.307587000000 -4.602071000000 -0.723337000000

H -1.994733000000 -5.376387000000 -1.039002000000

C -1.188690000000 -4.279993000000 0.624112000000

H -1.781642000000 -4.802717000000 1.364187000000

C -0.534424000000 -3.930737000000 -1.662519000000

H -0.616725000000 -4.180683000000 -2.712668000000

C 6.550945000000 -1.147561000000 -0.656138000000

H 7.513196000000 -1.504043000000 -1.000345000000

C -5.250991000000 -1.167684000000 -0.787034000000

H -5.167664000000 -2.233847000000 -1.000554000000

H -5.669015000000 -1.034044000000 0.213843000000

H -5.919789000000 -0.711634000000 -1.513326000000

N -3.948573000000 -0.553468000000 -0.916185000000

C 6.053850000000 0.065540000000 -1.118364000000

H 6.626501000000 0.655770000000 -1.822207000000

H -1.282563000000 2.488766000000 2.126073000000

H -1.442318000000 4.933123000000 1.849234000000

O 0.041254000000 6.354233000000 0.139935000000

H -0.633067000000 6.750096000000 0.703967000000

**4f′** (RSR_NO_2__o)

P 1.009829000000 -0.432068000000 0.533390000000

O 1.348464000000 -0.198152000000 1.937994000000

O -1.230334000000 -1.643542000000 1.972288000000

O 1.063017000000 -1.935982000000 0.000565000000

O 1.984556000000 0.370358000000 -0.470675000000

O -5.889499000000 0.926420000000 -0.730848000000

N -5.021198000000 -1.201541000000 -0.538118000000

N -1.697266000000 -0.706852000000 -0.070404000000

N -3.201015000000 -2.072800000000 0.837448000000

N -3.635431000000 0.494839000000 -0.955130000000

C -0.786491000000 1.582097000000 0.328684000000

C -0.202242000000 2.719558000000 -0.227397000000

C -1.975566000000 -1.495470000000 1.022235000000

C -4.947666000000 0.153012000000 -0.728154000000

C -1.136612000000 4.140225000000 1.448775000000

H -1.279918000000 5.124731000000 1.873265000000

C -2.791126000000 -0.677948000000 -1.017435000000

H -2.416811000000 -0.839113000000 -2.030877000000

C -0.566809000000 0.170188000000 -0.195550000000

H -0.320300000000 0.207518000000 -1.256751000000

C -1.731139000000 3.026698000000 2.028354000000

H -2.339025000000 3.136868000000 2.916869000000

C -0.352096000000 3.985108000000 0.318962000000

H 0.126548000000 4.829511000000 -0.157809000000

C -3.581028000000 -3.264629000000 1.573509000000

H -4.574069000000 -3.155108000000 2.006215000000

H -2.859873000000 -3.409651000000 2.374617000000

H -3.570047000000 -4.137349000000 0.915532000000

C -3.719921000000 -1.806022000000 -0.494543000000

H -3.696443000000 -2.718646000000 -1.095012000000

C -1.541802000000 1.765647000000 1.482655000000

H -1.988332000000 0.902570000000 1.959896000000

C -6.211607000000 -1.847412000000 -0.029648000000

H -7.082698000000 -1.376244000000 -0.479695000000

H -6.288545000000 -1.768440000000 1.057570000000

H -6.190995000000 -2.900020000000 -0.312542000000

C -3.312974000000 1.738211000000 -1.636146000000

H -4.121109000000 2.010098000000 -2.315042000000

H -2.394406000000 1.601629000000 -2.208351000000

H -3.159976000000 2.549588000000 -0.922682000000

C 2.315587000000 -2.515183000000 -0.228443000000

C 2.621176000000 -2.883911000000 -1.527112000000

C 3.204587000000 -2.702650000000 0.817136000000

C 3.862469000000 -3.449205000000 -1.787110000000

H 1.895693000000 -2.716993000000 -2.312781000000

C 4.446861000000 -3.260764000000 0.540536000000

H 2.927496000000 -2.401682000000 1.819302000000

C 4.778867000000 -3.631234000000 -0.756773000000

H 4.115683000000 -3.739257000000 -2.798908000000

H 5.156708000000 -3.404473000000 1.345095000000

H 5.748570000000 -4.065076000000 -0.964130000000

C 3.235155000000 0.846210000000 -0.064830000000

C 4.364729000000 0.247167000000 -0.594444000000

C 3.315920000000 1.923990000000 0.801009000000

C 5.613245000000 0.747485000000 -0.245611000000

H 4.259585000000 -0.598718000000 -1.263495000000

C 4.569507000000 2.411324000000 1.145309000000

H 2.409283000000 2.365482000000 1.196744000000

C 5.718229000000 1.826267000000 0.623698000000

H 6.504476000000 0.288560000000 -0.654668000000

H 4.646520000000 3.252115000000 1.822900000000

H 6.692751000000 2.211441000000 0.894861000000

N 0.586115000000 2.657626000000 -1.471644000000

O 0.166880000000 1.972019000000 -2.385586000000

O 1.594146000000 3.326407000000 -1.525240000000

**4d′** (RSR_NO_2__m)

P 1.099470000000 -0.576929000000 0.398408000000

O 1.486895000000 -0.281181000000 1.779547000000

O -1.110164000000 -1.793090000000 1.915625000000

O 1.117121000000 -2.103132000000 -0.071596000000

O 2.059108000000 0.150454000000 -0.672242000000

O -5.887042000000 0.501687000000 -0.788013000000

N -4.909021000000 -1.578752000000 -0.614855000000

N -1.601641000000 -0.925226000000 -0.153515000000

N -3.051998000000 -2.332449000000 0.778814000000

N -3.610343000000 0.193341000000 -1.003133000000

C -0.776733000000 1.392342000000 0.293643000000

C -0.241444000000 2.498914000000 -0.350002000000

C -1.853532000000 -1.699892000000 0.956763000000

C -4.905596000000 -0.220349000000 -0.788872000000

C -1.265294000000 3.954525000000 1.305142000000

H -1.443810000000 4.954089000000 1.673813000000

C -2.708774000000 -0.937212000000 -1.084975000000

H -2.343151000000 -1.074922000000 -2.105015000000

C -0.512921000000 0.007512000000 -0.267869000000

H -0.292041000000 0.103951000000 -1.335568000000

C -1.789238000000 2.838129000000 1.941374000000

H -2.391489000000 2.962591000000 2.831137000000

C -0.495178000000 3.755437000000 0.173659000000

C -3.391015000000 -3.520793000000 1.539708000000

H -4.377140000000 -3.427139000000 1.991664000000

H -2.650737000000 -3.640180000000 2.327620000000

H -3.374733000000 -4.401568000000 0.892718000000

C -3.577061000000 -2.110004000000 -0.558734000000

H -3.499474000000 -3.025376000000 -1.150570000000

C -1.541963000000 1.566261000000 1.444033000000

H -1.954481000000 0.701974000000 1.949969000000

C -6.066053000000 -2.288817000000 -0.114052000000

H -6.958597000000 -1.867886000000 -0.571681000000

H -6.155897000000 -2.211953000000 0.972254000000

H -5.984057000000 -3.339072000000 -0.394090000000

C -3.356395000000 1.457115000000 -1.675581000000

H -4.225246000000 1.733752000000 -2.271678000000

H -2.494445000000 1.346093000000 -2.335124000000

H -3.149472000000 2.254040000000 -0.959974000000

C 2.356081000000 -2.729075000000 -0.237328000000

C 2.700588000000 -3.141242000000 -1.513277000000

C 3.195720000000 -2.919664000000 0.847629000000

C 3.929966000000 -3.756977000000 -1.708157000000

H 2.014228000000 -2.967293000000 -2.331840000000

C 4.426972000000 -3.528937000000 0.636270000000

H 2.889178000000 -2.581927000000 1.828994000000

C 4.796796000000 -3.944711000000 -0.636990000000

H 4.213351000000 -4.081608000000 -2.701186000000

H 5.098063000000 -3.677381000000 1.472561000000

H 5.757772000000 -4.417318000000 -0.794364000000

C 3.286553000000 0.705124000000 -0.302883000000

C 4.448488000000 0.036872000000 -0.650498000000

C 3.315620000000 1.927281000000 0.347343000000

C 5.672564000000 0.616140000000 -0.338232000000

H 4.388059000000 -0.920012000000 -1.155499000000

C 4.544814000000 2.494895000000 0.655194000000

H 2.386801000000 2.416103000000 0.613615000000

C 5.723741000000 1.842364000000 0.313459000000

H 6.587489000000 0.102832000000 -0.605865000000

H 4.579001000000 3.449176000000 1.165283000000

H 6.679711000000 2.289043000000 0.554593000000

H 0.348323000000 2.396595000000 -1.252287000000

N 0.073701000000 4.925263000000 -0.512096000000

O -0.220409000000 6.027342000000 -0.095676000000

O 0.811442000000 4.726750000000 -1.456734000000

**4b′** (RSR_NO_2__p)

P 1.282354000000 -0.253982000000 0.343025000000

O 1.568983000000 0.378698000000 1.632288000000

O -0.763910000000 -1.491374000000 2.090566000000

O 1.554589000000 -1.822477000000 0.209034000000

O 2.142842000000 0.380629000000 -0.860985000000

O -5.693221000000 -0.976923000000 -1.260318000000

N -4.305289000000 -2.675838000000 -0.552932000000

N -1.292251000000 -1.174990000000 -0.120595000000

N -2.454306000000 -2.677414000000 1.042628000000

N -3.392051000000 -0.788564000000 -1.312657000000

C -0.904464000000 1.297928000000 -0.135998000000

C -0.506621000000 2.350216000000 -0.958182000000

C -1.439796000000 -1.759888000000 1.115151000000

C -4.581466000000 -1.430581000000 -1.053609000000

C -1.811331000000 3.842230000000 0.352509000000

C -2.263674000000 -1.660265000000 -1.074905000000

H -1.771300000000 -1.926081000000 -2.013514000000

C -0.386845000000 -0.098950000000 -0.419206000000

H -0.156084000000 -0.171170000000 -1.486581000000

C -2.222441000000 2.816239000000 1.187364000000

H -2.889430000000 3.021389000000 2.012019000000

C -0.957840000000 3.637500000000 -0.717593000000

C -2.540615000000 -3.757260000000 2.009291000000

H -3.569452000000 -3.905746000000 2.331709000000

H -1.935719000000 -3.487278000000 2.871708000000

H -2.161144000000 -4.687835000000 1.578605000000

C -2.898992000000 -2.862810000000 -0.329434000000

H -2.573603000000 -3.838198000000 -0.700047000000

C -1.756785000000 1.536361000000 0.938411000000

H -2.061530000000 0.722068000000 1.582780000000

C -5.318804000000 -3.495727000000 0.074190000000

H -6.252483000000 -3.369441000000 -0.469125000000

H -5.478454000000 -3.220351000000 1.119656000000

H -5.014049000000 -4.541047000000 0.020223000000

C -3.332677000000 0.324304000000 -2.242168000000

H -4.295904000000 0.412193000000 -2.741648000000

H -2.557647000000 0.138110000000 -2.989435000000

H -3.114084000000 1.262825000000 -1.731913000000

C 2.877950000000 -2.266669000000 0.138623000000

C 3.281883000000 -2.891957000000 -1.028497000000

C 3.738267000000 -2.081118000000 1.208061000000

C 4.593294000000 -3.336907000000 -1.129750000000

H 2.574884000000 -3.012517000000 -1.839062000000

C 5.050505000000 -2.523450000000 1.089306000000

H 3.383185000000 -1.589237000000 2.104508000000

C 5.480273000000 -3.147245000000 -0.075506000000

H 4.922905000000 -3.825169000000 -2.038000000000

H 5.737894000000 -2.378410000000 1.912851000000

H 6.503551000000 -3.489157000000 -0.161055000000

C 3.287384000000 1.147210000000 -0.628335000000

C 4.520530000000 0.594965000000 -0.929908000000

C 3.162538000000 2.444990000000 -0.162181000000

C 5.660807000000 1.369965000000 -0.759021000000

H 4.577481000000 -0.425571000000 -1.288935000000

C 4.310402000000 3.208143000000 0.007617000000

H 2.181351000000 2.840531000000 0.069073000000

C 5.558823000000 2.674170000000 -0.290198000000

H 6.630787000000 0.948708000000 -0.991508000000

H 4.225994000000 4.223095000000 0.374601000000

H 6.450347000000 3.273317000000 -0.156705000000

H 0.148110000000 2.161491000000 -1.801184000000

H -0.666979000000 4.464369000000 -1.349001000000

N -2.304006000000 5.202447000000 0.610487000000

O -1.919026000000 6.095002000000 -0.118421000000

O -3.070313000000 5.364073000000 1.539072000000

**4f″** (SRR_NO_2__o)

P 1.649001000000 -0.239788000000 -0.059513000000

O 1.630891000000 -0.333563000000 -1.525281000000

O 1.851299000000 1.234938000000 0.520723000000

O -0.808464000000 1.030249000000 2.667576000000

O -4.468805000000 -0.746714000000 -1.987477000000

O 2.800519000000 -1.025811000000 0.705780000000

N -0.917750000000 0.104026000000 0.548089000000

N -2.204700000000 1.849177000000 1.029672000000

N -2.252997000000 -0.483454000000 -1.423385000000

C -0.269503000000 -2.272453000000 0.367359000000

C -1.273241000000 1.005161000000 1.544401000000

C -1.446876000000 0.499090000000 -0.758253000000

H -0.646143000000 0.848540000000 -1.413280000000

C -2.457269000000 1.611373000000 -0.367376000000

H -2.323795000000 2.542094000000 -0.929852000000

C -3.582791000000 -0.138285000000 -1.414087000000

C 4.140167000000 -0.761625000000 0.379533000000

C 0.138905000000 -0.858464000000 0.769085000000

H 0.348463000000 -0.823325000000 1.842921000000

C 1.342019000000 2.392011000000 -0.075212000000

C -1.523137000000 -2.781130000000 0.706122000000

C 0.539793000000 -3.105926000000 -0.398425000000

C 4.730193000000 -1.451790000000 -0.664369000000

H 4.148493000000 -2.161222000000 -1.238500000000

C -1.147087000000 -4.828153000000 -0.455434000000

H -1.485730000000 -5.804263000000 -0.775247000000

C 0.656817000000 3.269016000000 0.750762000000

H 0.523407000000 3.018778000000 1.795773000000

C 1.562640000000 2.676129000000 -1.414725000000

H 2.120825000000 1.985131000000 -2.029745000000

C -2.698147000000 3.007276000000 1.742516000000

H -2.003275000000 3.243083000000 2.547288000000

H -3.686491000000 2.829959000000 2.169106000000

H -2.751797000000 3.853192000000 1.055123000000

C -1.984795000000 -4.016738000000 0.296424000000

H -2.978590000000 -4.335949000000 0.580949000000

C 4.836709000000 0.157911000000 1.143621000000

H 4.332329000000 0.675040000000 1.949514000000

C 0.116382000000 -4.371320000000 -0.791461000000

H 0.778123000000 -4.989956000000 -1.383417000000

C -1.732141000000 -1.426512000000 -2.398574000000

H -0.658985000000 -1.262810000000 -2.493992000000

H -2.210680000000 -1.270556000000 -3.365790000000

H -1.901832000000 -2.456626000000 -2.080325000000

C 6.174642000000 0.388177000000 0.850337000000

H 6.734987000000 1.102403000000 1.439845000000

C 0.337546000000 4.738201000000 -1.133340000000

H -0.059049000000 5.655321000000 -1.549393000000

C 0.154273000000 4.446728000000 0.213188000000

H -0.380049000000 5.137772000000 0.853329000000

C 1.044430000000 3.854611000000 -1.939203000000

H 1.205445000000 4.082079000000 -2.985293000000

C 6.790657000000 -0.293426000000 -0.192786000000

H 7.833553000000 -0.110072000000 -0.416966000000

N -3.732588000000 1.005466000000 -0.669351000000

C -4.951935000000 1.782905000000 -0.681075000000

H -5.267744000000 2.022781000000 0.334317000000

H -5.727377000000 1.191761000000 -1.162852000000

H -4.808426000000 2.712889000000 -1.238736000000

C 6.069071000000 -1.210835000000 -0.947114000000

H 6.546936000000 -1.742666000000 -1.759905000000

H 1.514010000000 -2.767966000000 -0.722902000000

N -2.434789000000 -2.018567000000 1.572666000000

O -3.577066000000 -1.863113000000 1.194454000000

O -1.995347000000 -1.626823000000 2.633652000000

**4d″** (SRR_NO_2__m)

P 1.750118000000 -0.328716000000 -0.133299000000

O 1.722055000000 -0.266684000000 -1.600079000000

O 2.246895000000 1.003268000000 0.598330000000

O -0.923365000000 0.915533000000 2.723832000000

O -4.352661000000 0.744802000000 -2.239734000000

O 2.713999000000 -1.405539000000 0.527793000000

N -0.707093000000 0.440875000000 0.476057000000

N -1.928438000000 2.197067000000 1.088837000000

N -2.253697000000 0.242886000000 -1.421767000000

C -0.506453000000 -2.017459000000 0.286694000000

C -1.173140000000 1.165868000000 1.558595000000

C -1.177199000000 0.974804000000 -0.788673000000

H -0.347228000000 1.111580000000 -1.484102000000

C -1.847609000000 2.306296000000 -0.356970000000

H -1.273281000000 3.197896000000 -0.627708000000

C -3.349514000000 1.055221000000 -1.620928000000

C 4.083835000000 -1.389177000000 0.222155000000

C 0.156611000000 -0.702912000000 0.664133000000

H 0.378572000000 -0.719100000000 1.736651000000

C 1.804370000000 2.285511000000 0.268831000000

C -1.769092000000 -2.251785000000 0.821353000000

C 0.066323000000 -2.971111000000 -0.546818000000

H 1.042519000000 -2.804786000000 -0.984769000000

C 4.534627000000 -2.047837000000 -0.907825000000

H 3.827818000000 -2.540020000000 -1.563151000000

C -1.871696000000 -4.387457000000 -0.332048000000

H -2.421591000000 -5.288190000000 -0.562319000000

C 1.318044000000 3.063200000000 1.308679000000

H 1.256554000000 2.640901000000 2.304298000000

C 1.900455000000 2.775276000000 -1.025025000000

H 2.294278000000 2.146960000000 -1.812074000000

C -2.264788000000 3.332384000000 1.928277000000

H -1.673987000000 3.282820000000 2.841916000000

H -3.323333000000 3.338857000000 2.187679000000

H -2.021889000000 4.254282000000 1.396108000000

C -2.420780000000 -3.425757000000 0.502247000000

C 4.943764000000 -0.743656000000 1.092783000000

H 4.544434000000 -0.242084000000 1.964574000000

C -0.612784000000 -4.147365000000 -0.853260000000

H -0.157438000000 -4.874556000000 -1.511788000000

C -1.995851000000 -0.850522000000 -2.348701000000

H -0.923484000000 -0.904101000000 -2.542420000000

H -2.522574000000 -0.666181000000 -3.284364000000

H -2.325061000000 -1.807078000000 -1.940280000000

C 6.305332000000 -0.763876000000 0.819655000000

H 6.991959000000 -0.266490000000 1.492616000000

C 0.984123000000 4.871368000000 -0.249784000000

H 0.662846000000 5.884512000000 -0.454165000000

C 0.911951000000 4.363604000000 1.043015000000

H 0.536546000000 4.979660000000 1.850523000000

C 1.476279000000 4.075546000000 -1.276927000000

H 1.542147000000 4.467380000000 -2.283928000000

C 6.783549000000 -1.418845000000 -0.309145000000

H 7.845530000000 -1.432032000000 -0.517488000000

N -3.105854000000 2.272079000000 -1.045301000000

C -4.121044000000 3.287714000000 -0.883476000000

H -4.610032000000 3.222261000000 0.091369000000

H -4.867280000000 3.151827000000 -1.662896000000

H -3.666578000000 4.273231000000 -0.991641000000

C 5.899275000000 -2.058589000000 -1.169375000000

H 6.269068000000 -2.569663000000 -2.048989000000

H -2.243464000000 -1.526128000000 1.469064000000

N -3.758280000000 -3.660921000000 1.069024000000

O -4.231540000000 -2.803711000000 1.787387000000

O -4.318484000000 -4.701117000000 0.787977000000

**4b″** (SRR_NO_2__p)

P -1.581192000000 0.463277000000 -0.046831000000

O -1.448728000000 0.519012000000 -1.508418000000

O -2.376933000000 -0.801364000000 0.521393000000

O 0.569565000000 -1.515337000000 2.834788000000

O 4.336717000000 -1.591062000000 -1.886843000000

O -2.372569000000 1.649440000000 0.654453000000

N 0.625085000000 -0.822517000000 0.635578000000

N 1.442235000000 -2.827358000000 1.147275000000

N 2.310505000000 -0.769374000000 -1.148866000000

C 0.916232000000 1.634578000000 0.669862000000

C 0.858341000000 -1.712360000000 1.668455000000

C 1.075787000000 -1.332158000000 -0.646235000000

H 0.285717000000 -1.249725000000 -1.394831000000

C 1.455931000000 -2.798066000000 -0.304515000000

H 0.751472000000 -3.539663000000 -0.695125000000

C 3.251016000000 -1.754877000000 -1.357500000000

C -3.665640000000 1.972893000000 0.214331000000

C -0.013795000000 0.450749000000 0.879501000000

H -0.309851000000 0.425178000000 1.934073000000

C -2.172447000000 -2.111833000000 0.085306000000

C 2.161179000000 1.576390000000 1.297655000000

C 0.586385000000 2.744224000000 -0.100600000000

H -0.367096000000 2.808081000000 -0.607529000000

C -3.817383000000 2.859892000000 -0.836497000000

H -2.944039000000 3.268672000000 -1.327804000000

C 2.715066000000 3.695651000000 0.377974000000

C -1.919413000000 -3.062983000000 1.062235000000

H -1.845592000000 -2.754431000000 2.097972000000

C -2.278760000000 -2.453494000000 -1.254309000000

H -2.486479000000 -1.690590000000 -1.992056000000

C 1.494508000000 -4.074103000000 1.887308000000

H 0.920630000000 -3.959218000000 2.805586000000

H 2.519216000000 -4.345822000000 2.139699000000

H 1.051380000000 -4.870108000000 1.284694000000

C 3.071864000000 2.606332000000 1.158874000000

C -4.747729000000 1.422317000000 0.877659000000

H -4.580692000000 0.731514000000 1.693706000000

C 1.489443000000 3.787044000000 -0.253147000000

H 1.249436000000 4.651047000000 -0.855609000000

C 2.322704000000 0.422221000000 -1.986201000000

H 1.292859000000 0.698711000000 -2.217472000000

H 2.857625000000 0.212498000000 -2.911985000000

H 2.804581000000 1.260246000000 -1.480667000000

C -6.027480000000 1.779096000000 0.471592000000

H -6.885126000000 1.358810000000 0.980963000000

C -1.852714000000 -4.754150000000 -0.653772000000

H -1.729097000000 -5.789264000000 -0.944556000000

C -1.765249000000 -4.390117000000 0.685508000000

H -1.577643000000 -5.140240000000 1.443651000000

C -2.106992000000 -3.785233000000 -1.616598000000

H -2.183047000000 -4.063020000000 -2.660178000000

C -6.206264000000 2.668841000000 -0.581094000000

H -7.205483000000 2.944495000000 -0.892424000000

N 2.748212000000 -2.945087000000 -0.909223000000

C 3.538582000000 -4.150267000000 -0.804909000000

H 3.984948000000 -4.261592000000 0.185825000000

H 4.332477000000 -4.103767000000 -1.547033000000

H 2.906281000000 -5.014466000000 -1.012002000000

C -5.102892000000 3.207185000000 -1.232385000000

H -5.239114000000 3.902618000000 -2.050499000000

H 2.421761000000 0.709302000000 1.892735000000

H 4.038930000000 2.571896000000 1.639054000000

N 3.677275000000 4.794048000000 0.215158000000

O 4.744834000000 4.703727000000 0.787853000000

O 3.355731000000 5.734968000000 -0.482745000000

**4f′** (SRS_NO_2__o)

P -1.009679000000 -0.432105000000 0.533842000000

O -1.348611000000 -0.198436000000 1.938402000000

O 1.230756000000 -1.642825000000 1.972705000000

O -1.062414000000 -1.936124000000 0.001162000000

O -1.984539000000 0.369948000000 -0.470328000000

O 5.889549000000 0.926387000000 -0.731078000000

N 5.021146000000 -1.201581000000 -0.538641000000

N 1.697460000000 -0.706251000000 -0.070045000000

N 3.201119000000 -2.072525000000 0.837442000000

N 3.635489000000 0.494935000000 -0.955504000000

C -2.314802000000 -2.515419000000 -0.228311000000

C 0.786144000000 1.582504000000 0.328649000000

C 0.201692000000 2.719739000000 -0.227717000000

C 1.975811000000 -1.494940000000 1.022477000000

C 4.947676000000 0.153040000000 -0.728537000000

C 1.135576000000 4.140879000000 1.448341000000

H 1.278618000000 5.125480000000 1.872698000000

C 2.790999000000 -0.677725000000 -1.017452000000

H 2.416328000000 -0.838926000000 -2.030752000000

C 0.566775000000 0.170424000000 -0.195307000000

H 0.320090000000 0.207443000000 -1.256470000000

C 0.351204000000 3.985401000000 0.318481000000

H -0.127579000000 4.829618000000 -0.158491000000

C 3.581026000000 -3.264356000000 1.573568000000

H 4.573703000000 -3.154633000000 2.007064000000

H 2.859306000000 -3.409815000000 2.374091000000

H 3.570823000000 -4.136924000000 0.915383000000

C 3.719769000000 -1.805884000000 -0.494683000000

H 3.696040000000 -2.718543000000 -1.095080000000

C 1.541294000000 1.766390000000 1.482651000000

C -2.620051000000 -2.883996000000 -1.527124000000

H -1.894408000000 -2.716847000000 -2.312598000000

C 6.211123000000 -1.847145000000 -0.028720000000

H 7.082616000000 -1.376649000000 -0.478678000000

H 6.287329000000 -1.767053000000 1.058473000000

H 6.190485000000 -2.900045000000 -0.310521000000

C -3.861188000000 -3.449417000000 -1.787510000000

H -4.114117000000 -3.739359000000 -2.799412000000

C 3.312888000000 1.738429000000 -1.636214000000

H 4.121085000000 2.010630000000 -2.314904000000

H 2.394413000000 1.601835000000 -2.208565000000

H 3.159640000000 2.549593000000 -0.922556000000

C -3.204072000000 -2.703174000000 0.816992000000

H -2.927292000000 -2.402305000000 1.819270000000

C -4.777827000000 -3.631748000000 -0.757433000000

H -5.747392000000 -4.065744000000 -0.965117000000

C -4.446227000000 -3.261410000000 0.540003000000

H -5.156255000000 -3.405316000000 1.344365000000

C -3.235147000000 0.845827000000 -0.064591000000

C -4.364660000000 0.246720000000 -0.594301000000

C -3.316034000000 1.923635000000 0.801177000000

C -5.613222000000 0.746983000000 -0.245599000000

H -4.259353000000 -0.599141000000 -1.263361000000

C -4.569687000000 2.410932000000 1.145344000000

H -2.409458000000 2.365218000000 1.196948000000

C -5.718328000000 1.825793000000 0.623681000000

H -6.504407000000 0.288020000000 -0.654713000000

H -4.646768000000 3.251759000000 1.822882000000

H -6.692901000000 2.210912000000 0.894733000000

C 1.730290000000 3.027582000000 2.028162000000

H 2.338057000000 3.138055000000 2.916721000000

H 1.987940000000 0.903473000000 1.960084000000

N -0.586578000000 2.657430000000 -1.471947000000

O -1.594854000000 3.325816000000 -1.525631000000

O -0.167131000000 1.971785000000 -2.385785000000

**4d′** (SRS_NO_2__m)

P -1.099478000000 -0.576920000000 0.398265000000

O -1.486884000000 -0.281230000000 1.779422000000

O 1.110069000000 -1.793257000000 1.915475000000

O -1.117218000000 -2.103113000000 -0.071813000000

O -2.059070000000 0.150590000000 -0.672327000000

O 5.887073000000 0.501690000000 -0.787770000000

N 4.909076000000 -1.578768000000 -0.614759000000

N 1.601648000000 -0.925312000000 -0.153611000000

N 3.051967000000 -2.332555000000 0.778750000000

N 3.610396000000 0.193316000000 -1.003067000000

C -2.356234000000 -2.728989000000 -0.237420000000

C 0.776798000000 1.392269000000 0.293577000000

C 0.241526000000 2.498873000000 -0.350060000000

C 1.853475000000 -1.700020000000 0.956646000000

C 4.905638000000 -0.220365000000 -0.788729000000

C 1.265392000000 3.954437000000 1.305132000000

H 1.443899000000 4.954001000000 1.673805000000

C 2.708844000000 -0.937248000000 -1.085003000000

H 2.343289000000 -1.074925000000 -2.105070000000

C 0.512952000000 0.007462000000 -0.267975000000

H 0.292114000000 0.103939000000 -1.335679000000

C 0.495272000000 3.755359000000 0.173642000000

C 3.390931000000 -3.520945000000 1.539589000000

H 4.377127000000 -3.427412000000 1.991414000000

H 2.650747000000 -3.640232000000 2.327604000000

H 3.374454000000 -4.401723000000 0.892606000000

C 3.577114000000 -2.110049000000 -0.558746000000

H 3.499593000000 -3.025396000000 -1.150632000000

C 1.542015000000 1.566162000000 1.443960000000

C -2.700866000000 -3.141189000000 -1.513326000000

H -2.014561000000 -2.967311000000 -2.331950000000

C 6.066083000000 -2.288821000000 -0.113880000000

H 6.958650000000 -1.867961000000 -0.571530000000

H 6.155915000000 -2.211868000000 0.972421000000

H 5.984059000000 -3.339097000000 -0.393830000000

C -3.930294000000 -3.756868000000 -1.708079000000

H -4.213777000000 -4.081531000000 -2.701069000000

C 3.356490000000 1.457115000000 -1.675476000000

H 4.225355000000 1.733743000000 -2.271561000000

H 2.494544000000 1.346139000000 -2.335030000000

H 3.149591000000 2.254028000000 -0.959850000000

C -3.195792000000 -2.919491000000 0.847615000000

H -2.889148000000 -2.581730000000 1.828939000000

C -4.797045000000 -3.944509000000 -0.636834000000

H -5.758059000000 -4.417070000000 -0.794111000000

C -4.427095000000 -3.528703000000 0.636379000000

H -5.098126000000 -3.677081000000 1.472730000000

C -3.286522000000 0.705224000000 -0.302895000000

C -4.448472000000 0.036936000000 -0.650387000000

C -3.315558000000 1.927393000000 0.347306000000

C -5.672529000000 0.616184000000 -0.338017000000

H -4.388079000000 -0.919949000000 -1.155389000000

C -4.544737000000 2.494989000000 0.655262000000

H -2.386729000000 2.416246000000 0.613479000000

C -5.723677000000 1.842423000000 0.313654000000

H -6.587467000000 0.102850000000 -0.605556000000

H -4.578892000000 3.449279000000 1.165334000000

H -6.679639000000 2.289076000000 0.554869000000

C 1.789313000000 2.838027000000 1.941336000000

H 2.391556000000 2.962426000000 2.831114000000

H 1.954521000000 0.701866000000 1.949892000000

H -0.348256000000 2.396578000000 -1.252338000000

N -0.073608000000 4.925227000000 -0.512083000000

O 0.220434000000 6.027282000000 -0.095561000000

O -0.811279000000 4.726742000000 -1.456777000000

**4b′** (SRS_NO_2__p)

P -1.282359000000 -0.253954000000 0.343035000000

O -1.568981000000 0.378758000000 1.632283000000

O 0.763909000000 -1.491365000000 2.090575000000

O -1.554614000000 -1.822447000000 0.209078000000

O -2.142846000000 0.380635000000 -0.860988000000

O 5.693185000000 -0.976963000000 -1.260357000000

N 4.305255000000 -2.675867000000 -0.552940000000

N 1.292229000000 -1.175007000000 -0.120596000000

N 2.454281000000 -2.677432000000 1.042629000000

N 3.392016000000 -0.788604000000 -1.312684000000

C -2.877980000000 -2.266621000000 0.138661000000

C 0.904492000000 1.297917000000 -0.135988000000

C 0.506624000000 2.350226000000 -0.958133000000

C 1.439782000000 -1.759895000000 1.115155000000

C 4.581432000000 -1.430616000000 -1.053632000000

C 1.811415000000 3.842203000000 0.352529000000

C 2.263638000000 -1.660297000000 -1.074912000000

H 1.771251000000 -1.926120000000 -2.013512000000

C 0.386843000000 -0.098949000000 -0.419199000000

H 0.156080000000 -0.171158000000 -1.486575000000

C 0.957873000000 3.637501000000 -0.717543000000

C 2.540591000000 -3.757272000000 2.009299000000

H 3.569427000000 -3.905751000000 2.331724000000

H 1.935688000000 -3.487289000000 2.871711000000

H 2.161127000000 -4.687850000000 1.578616000000

C 2.898960000000 -2.862838000000 -0.329435000000

H 2.573568000000 -3.838228000000 -0.700039000000

C 1.756866000000 1.536323000000 0.938386000000

C -3.281900000000 -2.891958000000 -1.028438000000

H -2.574887000000 -3.012564000000 -1.838984000000

C 5.318775000000 -3.495764000000 0.074164000000

H 6.252452000000 -3.369458000000 -0.469148000000

H 5.478422000000 -3.220411000000 1.119636000000

H 5.014027000000 -4.541085000000 0.020174000000

C -4.593314000000 -3.336896000000 -1.129695000000

H -4.922914000000 -3.825197000000 -2.037928000000

C 3.332639000000 0.324262000000 -2.242199000000

H 4.295857000000 0.412136000000 -2.741700000000

H 2.557594000000 0.138072000000 -2.989451000000

H 3.114065000000 1.262787000000 -1.731942000000

C -3.738314000000 -2.081009000000 1.208074000000

H -3.383245000000 -1.589091000000 2.104506000000

C -5.480311000000 -3.147173000000 -0.075476000000

H -6.503591000000 -3.489076000000 -0.161028000000

C -5.050556000000 -2.523329000000 1.089314000000

H -5.737958000000 -2.378240000000 1.912840000000

C -3.287388000000 1.147219000000 -0.628354000000

C -4.520530000000 0.594977000000 -0.929955000000

C -3.162551000000 2.444994000000 -0.162184000000

C -5.660809000000 1.369976000000 -0.759084000000

H -4.577473000000 -0.425558000000 -1.288985000000

C -4.310418000000 3.208146000000 0.007599000000

H -2.181368000000 2.840533000000 0.069092000000

C -5.558833000000 2.674177000000 -0.290247000000

H -6.630784000000 0.948723000000 -0.991596000000

H -4.226016000000 4.223094000000 0.374593000000

H -6.450361000000 3.273323000000 -0.156768000000

C 2.222550000000 2.816189000000 1.187342000000

H 2.889580000000 3.021312000000 2.011972000000

H 2.061640000000 0.722016000000 1.582723000000

H -0.148151000000 2.161528000000 -1.801106000000

H 0.666998000000 4.464379000000 -1.348930000000

N 2.304112000000 5.202413000000 0.610487000000

O 3.070413000000 5.364030000000 1.539086000000

O 1.919140000000 6.094975000000 -0.118425000000

**4f″** (RSS_NO_2__o)

P -1.648949000000 -0.239834000000 -0.059694000000

O -1.630738000000 -0.333571000000 -1.525463000000

O -1.851265000000 1.234870000000 0.520575000000

O 0.807997000000 1.030257000000 2.667560000000

O 4.468972000000 -0.746499000000 -1.987367000000

O -2.800515000000 -1.025894000000 0.705499000000

N 0.917769000000 0.103902000000 0.548167000000

N 2.204600000000 1.849118000000 1.029902000000

N 2.253154000000 -0.483426000000 -1.423282000000

C 0.269469000000 -2.272552000000 0.367316000000

C 1.272994000000 1.005143000000 1.544483000000

C 1.446955000000 0.499035000000 -0.758128000000

H 0.646247000000 0.848481000000 -1.413187000000

C 2.457278000000 1.611337000000 -0.367142000000

H 2.323800000000 2.542057000000 -0.929622000000

C 3.582913000000 -0.138174000000 -1.413944000000

C -4.140155000000 -0.761601000000 0.379285000000

C -0.138942000000 -0.858555000000 0.769026000000

H -0.348616000000 -0.823448000000 1.842839000000

C -1.341882000000 2.391965000000 -0.075228000000

C 1.523055000000 -2.781272000000 0.706207000000

C -0.539767000000 -3.105991000000 -0.398568000000

H -1.513938000000 -2.768000000000 -0.723150000000

C -4.730245000000 -1.451761000000 -0.664583000000

H -4.148599000000 -2.161253000000 -1.238696000000

C 1.147074000000 -4.828261000000 -0.455437000000

H 1.485727000000 -5.804373000000 -0.775231000000

C -0.656819000000 3.268932000000 0.750907000000

H -0.523545000000 3.018626000000 1.795919000000

C -1.562286000000 2.676153000000 -1.414761000000

H -2.120366000000 1.985185000000 -2.029907000000

C 2.697460000000 3.007599000000 1.742535000000

H 2.003172000000 3.242239000000 2.548143000000

H 3.686467000000 2.831297000000 2.167993000000

H 2.749312000000 3.853852000000 1.055410000000

C 1.984720000000 -4.016883000000 0.296529000000

H 2.978469000000 -4.336142000000 0.581157000000

C -4.836620000000 0.158002000000 1.143360000000

H -4.332201000000 0.675121000000 1.949235000000

C -0.116347000000 -4.371387000000 -0.791590000000

H -0.778043000000 -4.989990000000 -1.383631000000

C 1.732384000000 -1.426524000000 -2.398476000000

H 0.659245000000 -1.262783000000 -2.494042000000

H 2.211058000000 -1.270619000000 -3.365631000000

H 1.901995000000 -2.456628000000 -2.080160000000

C -6.174543000000 0.388347000000 0.850089000000

H -6.734831000000 1.102627000000 1.439585000000

C -0.337306000000 4.738250000000 -1.133053000000

H 0.059314000000 5.655412000000 -1.548988000000

C -0.154241000000 4.446700000000 0.213486000000

H 0.379908000000 5.137744000000 0.853771000000

C -1.044018000000 3.854678000000 -1.939085000000

H -1.204866000000 4.082204000000 -2.985188000000

C -6.790624000000 -0.293250000000 -0.192999000000

H -7.833512000000 -0.109834000000 -0.417164000000

C 4.951848000000 1.783213000000 -0.681299000000

H 4.808327000000 2.712549000000 -1.240047000000

H 5.267312000000 2.024242000000 0.333916000000

H 5.727544000000 1.191673000000 -1.162181000000

N 3.732668000000 1.005483000000 -0.668997000000

C -6.069113000000 -1.210729000000 -0.947314000000

H -6.547026000000 -1.742553000000 -1.760080000000

N 2.434658000000 -2.018758000000 1.572844000000

O 1.995162000000 -1.627007000000 2.633800000000

O 3.576944000000 -1.863270000000 1.194673000000

**4d″** (RSS_NO_2__m)

P -1.750038000000 -0.328896000000 -0.133633000000

O -1.721922000000 -0.266860000000 -1.600413000000

O -2.247125000000 1.002970000000 0.597970000000

O 0.922448000000 0.915997000000 2.723738000000

O 4.353096000000 0.745123000000 -2.239344000000

O -2.713767000000 -1.405899000000 0.527379000000

N 0.707056000000 0.440939000000 0.475970000000

N 1.927949000000 2.197376000000 1.088852000000

N 2.254080000000 0.242922000000 -1.421600000000

C 0.506659000000 -2.017406000000 0.286347000000

C 1.172617000000 1.166176000000 1.558550000000

C 1.177421000000 0.974715000000 -0.788713000000

H 0.347545000000 1.111358000000 -1.484310000000

C 1.847579000000 2.306348000000 -0.356986000000

H 1.273162000000 3.197794000000 -0.628051000000

C 3.349814000000 1.055424000000 -1.620696000000

C -4.083660000000 -1.389523000000 0.222011000000

C -0.156527000000 -0.702968000000 0.663875000000

H -0.378497000000 -0.719353000000 1.736391000000

C -1.804693000000 2.285304000000 0.268747000000

C 1.769242000000 -2.251694000000 0.821119000000

C -0.066018000000 -2.971106000000 -0.547196000000

H -1.042137000000 -2.804765000000 -0.985302000000

C -4.534617000000 -2.047765000000 -0.908141000000

H -3.827898000000 -2.539655000000 -1.563782000000

C 1.871988000000 -4.387422000000 -0.332178000000

H 2.421938000000 -5.288159000000 -0.562315000000

C -1.319126000000 3.063014000000 1.308943000000

H -1.258243000000 2.640644000000 2.304554000000

C -1.900151000000 2.775217000000 -1.025115000000

H -2.293480000000 2.146964000000 -1.812460000000

C 2.264014000000 3.332856000000 1.928165000000

H 1.673608000000 3.282863000000 2.842031000000

H 3.322658000000 3.339876000000 2.187146000000

H 2.020403000000 4.254659000000 1.396153000000

C 2.420989000000 -3.425656000000 0.502104000000

C -4.943471000000 -0.744385000000 1.093051000000

H -4.543997000000 -0.243153000000 1.964969000000

C 0.613135000000 -4.147367000000 -0.853521000000

H 0.157842000000 -4.874598000000 -1.512041000000

C 1.996600000000 -0.850647000000 -2.348433000000

H 0.924373000000 -0.903914000000 -2.543033000000

H 2.524170000000 -0.666695000000 -3.283697000000

H 2.325250000000 -1.807149000000 -1.939483000000

C -6.305073000000 -0.764566000000 0.820149000000

H -6.991619000000 -0.267490000000 1.493420000000

C -0.984590000000 4.871390000000 -0.249143000000

H -0.663322000000 5.884595000000 -0.453236000000

C -0.913043000000 4.363490000000 1.043625000000

H -0.538221000000 4.979524000000 1.851422000000

C -1.476088000000 4.075590000000 -1.276628000000

H -1.541458000000 4.467483000000 -2.283638000000

C -6.783461000000 -1.419121000000 -0.308831000000

H -7.845481000000 -1.432255000000 -0.516979000000

C 4.120960000000 3.288069000000 -0.883189000000

H 3.666289000000 4.273465000000 -0.991620000000

H 4.609811000000 3.222921000000 0.091742000000

H 4.867349000000 3.152165000000 -1.662461000000

N 3.106018000000 2.272179000000 -1.044944000000

C -5.899321000000 -2.058492000000 -1.169458000000

H -6.269234000000 -2.569232000000 -2.049214000000

H 2.243618000000 -1.525984000000 1.468776000000

N 3.758443000000 -3.660732000000 1.069009000000

O 4.231543000000 -2.803467000000 1.787412000000

O 4.318759000000 -4.700876000000 0.787978000000

**4b″** (RSS_NO_2__p)

P -1.581344000000 -0.462742000000 -0.046724000000

O -1.448655000000 -0.518491000000 -1.508300000000

O -2.376845000000 0.802086000000 0.521397000000

O 0.570112000000 1.515256000000 2.834917000000

O 4.336919000000 1.589053000000 -1.887211000000

O -2.373058000000 -1.648762000000 0.654373000000

N 0.625225000000 0.822480000000 0.635674000000

N 1.443200000000 2.826975000000 1.147406000000

N 2.310299000000 0.768555000000 -1.149079000000

C 0.915742000000 -1.634713000000 0.669987000000

C 0.858902000000 1.712191000000 1.668573000000

C 1.076021000000 1.332011000000 -0.646168000000

H 0.285780000000 1.250122000000 -1.394635000000

C 1.457057000000 2.797676000000 -0.304361000000

H 0.753114000000 3.539725000000 -0.695047000000

C 3.251426000000 1.753461000000 -1.357607000000

C -3.666284000000 -1.971729000000 0.214386000000

C -0.013970000000 -0.450630000000 0.879653000000

H -0.309904000000 -0.424953000000 1.934256000000

C -2.171930000000 2.112473000000 0.085217000000

C 2.160887000000 -1.576704000000 1.297403000000

C 0.585413000000 -2.744439000000 -0.100161000000

H -0.368231000000 -2.808176000000 -0.606786000000

C -3.818334000000 -2.859026000000 -0.836150000000

H -2.945118000000 -3.268247000000 -1.327325000000

C 2.713997000000 -3.696280000000 0.377963000000

C -1.918426000000 3.063581000000 1.062062000000

H -1.844637000000 2.755068000000 2.097813000000

C -2.278231000000 2.454069000000 -1.254412000000

H -2.486312000000 1.691185000000 -1.992079000000

C 1.496066000000 4.073700000000 1.887417000000

H 0.922053000000 3.959159000000 2.805654000000

H 2.520897000000 4.344899000000 2.139878000000

H 1.053385000000 4.869908000000 1.284743000000

C 3.071295000000 -2.606879000000 1.158529000000

C -4.748184000000 -1.420462000000 0.877448000000

H -4.580934000000 -0.729431000000 1.693259000000

C 1.488164000000 -3.787511000000 -0.252762000000

H 1.247748000000 -4.651587000000 -0.854958000000

C 2.321699000000 -0.422964000000 -1.986522000000

H 1.291617000000 -0.699233000000 -2.216987000000

H 2.855961000000 -0.213319000000 -2.912711000000

H 2.803802000000 -1.261106000000 -1.481401000000

C -6.028050000000 -1.776909000000 0.471445000000

H -6.885567000000 -1.356171000000 0.980658000000

C -1.851140000000 4.754587000000 -0.654078000000

H -1.727075000000 5.789624000000 -0.944946000000

C -1.763721000000 4.390620000000 0.685223000000

H -1.575714000000 5.140720000000 1.443292000000

C -2.105931000000 3.785711000000 -1.616811000000

H -2.181963000000 4.063458000000 -2.660404000000

C -6.207134000000 -2.666975000000 -0.580920000000

H -7.206452000000 -2.942374000000 -0.892162000000

C 3.540461000000 4.148697000000 -0.804956000000

H 2.908680000000 5.013167000000 -1.012510000000

H 3.986708000000 4.260161000000 0.185811000000

H 4.334458000000 4.101453000000 -1.546923000000

N 2.749510000000 2.943880000000 -0.908920000000

C -5.103949000000 -3.205973000000 -1.231986000000

H -5.240396000000 -3.901612000000 -2.049886000000

H 2.421864000000 -0.709557000000 1.892225000000

H 4.038522000000 -2.572592000000 1.638391000000

N 3.675878000000 -4.794974000000 0.215135000000

O 4.743765000000 -4.704650000000 0.787235000000

O 3.353763000000 -5.736123000000 -0.482202000000
